# Metal‐Free, Chemoselective Reduction of Aromatic Nitro Compounds in Water at Room Temperature

**DOI:** 10.1002/open.70219

**Published:** 2026-05-14

**Authors:** Maria Batzaki, Thomas S. A. Heugebaert, Christian V. Stevens

**Affiliations:** ^1^ Department of Green Chemistry and Technology Faculty of Bioscience Engineering Campus Coupure Links Ghent University Gent Belgium

**Keywords:** aqueous medium reactions, chemoselective reduction of nitroarenes, green and sustainable chemistry, metal‐free reduction methods, room temperature organic synthesis

## Abstract

A metal‐free, rapid, and chemoselective protocol for the nitro reduction of aromatic compounds is reported using tetrahydroxydiboron as reducing agent and 4,4′‐bipyridine as organocatalyst. This method provides the transformation to aromatic amines with high functional group tolerance in good to excellent yields, under mild conditions (ambient temperature) and water as solvent.

## Graphical abstract

A metal‐free, rapid, and chemoselective nitro reduction of aromatic compounds is developed using tetrahydroxydiboron as reducing agent and 4,4^′^‐bipyridine as organocatalyst. This green method provides an efficient entry to aromatic amines with high functional group tolerance in excellent yields under mild conditions using water as solvent.

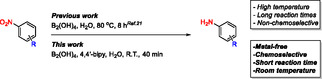

## 1 Introduction

**SCHEME 1:**
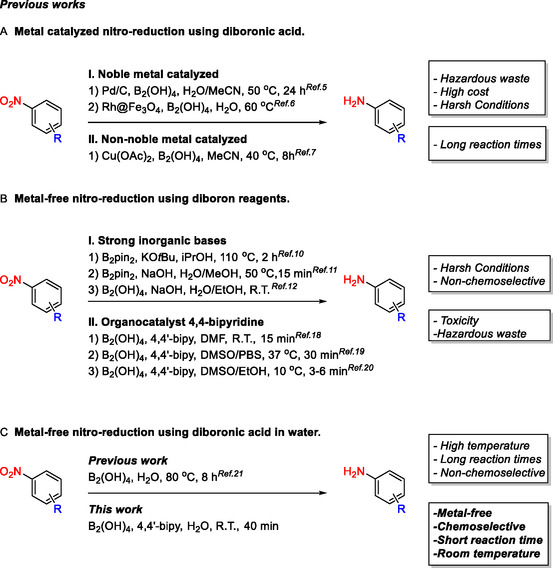
Different methods for reduction of aromatic nitro compounds.

The chemo‐selective reduction of aromatic nitro compounds is fundamental for the preparation of the corresponding amines, which are crucial building blocks for pharmaceuticals, agrochemicals, and advanced materials. Traditional reduction methods, such as catalytic hydrogenation (H_2_ on Pd/C, Pt/C, Raney Ni, Ru/C), classical metal–proton methods (Fe/HCl, Zn/NH_4_Cl, In/HCl, SnCl_2_/HCl), and transfer‐hydrogenation (formate, iPrOH, hydrosilanes) most often lack chemo‐selectivity, generate large amounts of hazardous waste and require harsh conditions and/or the use of precious metal catalysts. Even comparatively mild systems such as Zn/NH_4_Cl in water, remain problematic due selectivity issues and the use of stoichiometric amounts of metals, which result in metal‐containing waste streams. Indium‐based methods are further limited by the relatively high cost and its limited availability. The use of noble or non‐noble metal catalysts (Pd/Rh/Cu) combined with tetrahydroxydiboron offers a higher catalytic activity and improved selectivity; nevertheless, these methods require long reaction times (Scheme 1A). Therefore, the development of cost‐effective, safer and more sustainable strategies for the preparation of aromatic amines remains crucial for improving the processes used in the industry [1, 2, 3, 4, 5, 6, 7].

Metal‐free reductions of nitro groups are particularly attractive due to the avoidance of metal contamination, simplified waste handling, and reduced safety concerns associated with pressurized hydrogenation. Various metal‐free reduction methods for nitro groups have been developed using hydriodic acid, sodium dithionite, dihydroanthracene, hydrazine with carbon‐based catalysts, (2‐pyridyl)phenyl methanol derivatives, thiols, D‐glucose or natural alkaloids, and trichlorosilane. However, these approaches present several limitations, such as harsh reaction conditions (HI, DHA), safety risks upon scale‐up (Na_2_S_2_O_4_, hydrazine), the need for large reagent excess and formation of significant byproducts (DHA, alcohol‐based reductants), limited substrate scope and side reactions (thiols), high temperatures and purification challenges (glucose, alkaloids), or the requirement for careful handling of reactive reagents (HSiCl_3_) [8].

Diboron compounds, introduced in 1925, represent a versatile class of reagents widely applied in the synthesis of natural products, pharmaceutical intermediates, and biologically active molecules, and are considered attractive for green chemistry due to their low toxicity and favorable environmental profile [9]. In 2016, the first metal‐free and high‐yielding method for the nitro reduction of nitroarenes was reported using Bis(pinacolato)diboronate (B_2_pin_2_) activated by KO*t*‐Bu in isopropanol. Although, this method is selective towards reducible groups such as cyano, ester, and alkynyl groups, the nitro reduction of 4‐nitrobenzaldehyde was unsuccessful [10]. Another method was developed using strong basic conditions (NaOH) and B_2_pin_2_ in aqueous methanol at 50°C. This approach shows chemoselectivity towards nitroquinolines or nitropyridines, however 8‐aminoquinoline was obtained in moderate yield. Aniline was obtained in low yield, even after using longer reaction times. Furthermore, the reduction of 4‐nitrobenzaldehyde failed, yielding the corresponding alcohol, indicating that the aldehyde group is not tolerated under such harsh conditions [11]. Additionally, a DNA compatible method for nitro reduction using strong basic conditions (NaOH) and B_2_(OH)_4_ in aqueous ethanol was developed (Scheme 1B). Nonetheless, these harsh basic conditions also resulted in limited tolerance of sensitive functional groups such as aldehydes and nitriles [12]. Although, these methods proceed without organic ligands and employ greener solvents (according to literature) [13, 14, 15, 16, 17], a major limitation is their reduced chemoselectivity.

The use of 4,4′‐bipyridine as a catalyst in combination with B_2_(OH)_4_ as a reducing agent was introduced for a rapid and highly chemoselective reduction of nitroarenes, providing excellent yields in dimethyl formamide (DMF) at ambient temperature [18]. Nevertheless, the use of DMF raises concerns due to its toxicity and environmental impact [13, 14, 15, 16, 17]. Moreover, prodrug activation via biocompatible aromatic nitro reduction at low micromolar concentrations was achieved using 4,4′‐bipyridine as mediator and B_2_(OH)_4_ as a reducing agent in a phosphate buffered saline PBS/DMSO 9:1 solution. NMR studies revealed the significance of water in the formation of a complex between the organocatalyst and the reducing agent [19]. Apart from the limited substrate scope, a drawback of this method is the requirement for relatively high equivalents of the reducing agent B_2_(OH)_4_ (20 equivalents) and the mediator 4,4′‐bipyridine (5 equivalents), which may limit both its efficiency and sustainability. Finally, a safer continuous flow process was developed using 4,4′‐bipyridine again as a catalyst and B_2_(OH)_4_ as a reductant. The thermal stability of B_2_(OH)_4_ and the chemoselectivity of the reaction in DMSO were enhanced by using EtOH as a protic cosolvent. This method allows safer scaleup and demonstrates selectivity towards some reducible or sensitive groups; however, nitroquinolines and nitropyridines were not tested [20]. Although these methods are generally chemoselective due to the organocatalyst 4,4′‐bipyridine, their reliance on organic solvents such as DMF and DMSO represents a significant limitation from a green chemistry perspective (Scheme 1B) [13, 14, 15, 16, 17].

A metal‐free protocol for nitro reduction in water, using (B_2_(OH)_4_) as a reducing agent, represented a significant innovation published by Chen et al. In this reaction, water serves as a hydrogen source and as a solvent, and the reaction is carried out at 80°C (Scheme 1C). The reaction exhibits high chemoselectivity for nitroarenes containing reducible groups such as cyano and carbonyl moieties, providing moderate to high yields. Remarkably, overreduction occurs in the case of nitroquinolines and nitropyridines. Nitroquinolines, with a nitro group at the 5‐ or 8‐ position, are fully over‐reduced to the corresponding amino‐substituted 1,2,3,4‐tetrahydroquinolines, whereas 6‐aminoquinoline, 2‐methyl‐8‐nitroquinoline and 3‐amino‐2‐chloropyridine are obtained in moderate yields [21]. Similar conditions, tetrahydroxydiboron in water at 80°C, are used for the hydrogenation of N‐heterocycles, including quinolines which explains the full over‐reduction of nitroquinolines under these conditions [22]. Despite the use of water, which is abundant, inexpensive, and green [13, 14, 15, 16, 17] due to its nontoxicity, renewability, safety, and ease of handling and treatment, the reaction exhibits poor chemoselectivity.

Although several methods have been developed in recent years, limitations remain due to the high cost of precious metal catalysts, and the harsh conditions often required, such as high temperatures, strong bases or long reaction times. Many methods also suffer from poor chemoselectivity, over‐reduction of N‐heterocycles, or incompatibility with sensitive functional groups such as aldehydes, nitriles, or alkynes. Moreover, some protocols require high equivalents of reducing agents or organocatalysts, leading to reduced efficiency and sustainability. In addition, most reactions carried out in water or aqueous alcohols without 4,4′‐bipyridine encounter selectivity issues, highlighting the crucial role of the organocatalyst in achieving chemoselectivity. In our study, the aromatic nitro reduction is rapid and highly chemoselective, proceeding at room temperature in pure water using only 4,4′‐bipyridine as the organocatalyst and B_2_(OH)_4_ as the reductant. The formation of a complex between 4,4′‐bipyridine and the diboronic acid, stabilized by water, enhances selectivity by controlling the reactivity of the reducing agent. Furthermore, the method is operationally simple and employs nontoxic, widely available reagents in water, offering a milder and potentially greener alternative to conventional protocols, while avoiding precious metals and harsh reaction conditions (Scheme 1C).

## 2 Results and Discussion

6‐nitroquinoline **1a** was selected as model substrate for the screening of the reaction conditions due to its structural similarity to previously reported nitroquinolines that showed poor chemoselectivity under similar conditions [21]. Moreover, the reduction of nitroarenes performed in DMF proved to be the most efficient and selective among the conditions reported in the literature, affording 6‐aminoquinoline in high isolated yield [18]. This also made it an appropriate model substrate for assessing selectivity and efficiency under greener solvent and thermal conditions. To improve the environmental aspects of the process, a series of greener solvents, 2‐methyltetrahydrofuran (2‐MeTHF), methyl tert‐butyl ether (MTBE), dimethyl carbonate (DMC), ethanol (EtOH), methanol (MeOH), ethyl acetate (EtOAc), and dry dimethyl sulfoxide (DMSO), were evaluated as alternatives. Among these, dry DMSO afforded the highest yield under mild conditions (RT), identifying it as the most suitable green organic solvent for this transformation (Table 1, entry 1–8).

**TABLE 1:** Green solvent screening and optimization of reaction conditions for the reduction of 6‐nitroquinoline.

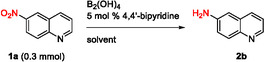						
Entry	Solvent	C (M)	B_2_(OH)_4_ (eq.)	t, min	T, (°C)	Conversion (%)^a^
1	DMF	0.15	3	5	RT	95^b^
2	MTBE	0.06	3	30	55	n.r.
3	EtOAc	0.06	3	40	60	4
4	DMC	0.06	3	60	90	6
5	EtOH	0.06	3	40	60	10
6	MeOH	0.06	3	40	40	12
7	2‐MeTHF	0.06	3	60	90	65
**Entry**	**Solvent**	**C (M)**	**B_2_(OH)_4_ (eq.)**	**t, min**	**T, (°C)**	**Yield (%)** c
8	DMSO (dry)	0.22	3	15	RT	55
9	DMSO (dry)	0.22	3	15	50	60
10	DMSO (dry)	0.22	3.5	15	RT	76
11	DMSO (dry)	0.22	4	15	RT	94 (97)^d^
12	H_2_O	0.22	4	15	RT	50
13	H_2_O	0.22	4	30	RT	78
14	H_2_O	0.22	3	40	RT	83
15	H_2_O	0.22	3.5	40	RT	86
16^e^	H_2_O	0.22	4	40	RT	(82)^d^
17^f^	H_2_O	0.22	4	40	RT	99 (81)^d^

a Determined by LC–MS.

b Isolated yield previously reported by Jang et al. [18].

c Yield determined by LC–MS calibration curve (Figure S1).

d Isolated yield in parentheses.

e Substrate scale: **1a** (0.6 mmol).

f Substrate scale: **1a** (9 mmol).

The high reaction rate, yield, and homogeneity of the reaction mixture (clear yellow solution) in dry DMSO, allow the reaction to be carried out at increased concentration (entry 8). Increasing the temperature (entry 9) did not show a significant improvement of the yield. On the contrary, the amount of tetrahydroxydiboron plays a crucial role in the yield with the addition of 4 equivalents of the reductant (entry 11) giving excellent yield (Table 1, entry 8–11).

Although the reaction proceeded efficiently in dry DMSO, the use of water as reaction solvent was tested to improve the overall process sustainability. Prolonged reaction times resulted in a significant increase in reaction yield, from 50% (entry 12) to 99% (entry 17). However, increasing the amount of B_2_(OH)_4_ led to a slightly improved yield, from 83% (entry 14) to 99% yield (entry 17) (yield determined according to a liquid chromatography‐mass spectrometry (LC–MS) calibration curve, Figure S1). The isolated yield of the nitro reduction performed in H_2_O (entry 17) deviated slightly from the yield determined according to the LC–MS calibration curve, while in the case of DMSO (entry 11) it was in good alignment. The isolated yield (entry 17) obtained on a 15‐fold scale up (1.5 gram‐scale) was in agreement with the isolated yield of entry 16 (100 mg‐scale), showing the reproducibility of the method (Table 1, entry 11–17).

**SCHEME 2:**
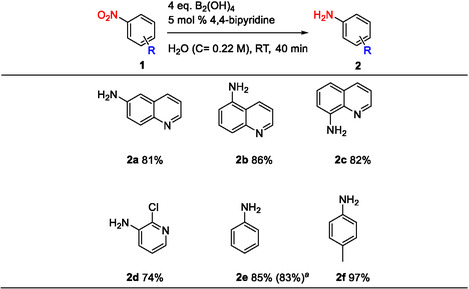
Nitro reduction of nitroquinolines and nitropyridines. Isolated yields. Substrate scale: **1a** (1.5 g), **1b‐1e** (1.0 g), **1f** (100 mg), ^a^ **1e** (120 mg) in parentheses.

After determining the optimal reaction conditions, the reduction protocol was applied to a series of substituted nitro compounds to explore its substrate scope and chemoselectivity. Initially, nitroquinolines and nitropyridines were selected, as previous reported nitro reductions in water led to overreduction [21]. The reaction selectively reduced the nitro group and no hydrogenation of the N‐heterocycle was observed. Aminoquinolines **2a‐2c** were isolated in high yields (81%−86% yield) and 2‐chloropyridin‐3‐amine **2d** in 74% yield, while the reactions were carried out on a gram‐scale showing the robustness and applicability of the method (Scheme 2, **2a‐2d**). Similar reproducible results were obtained for aniline **2e** on a 8‐fold scale up (120 mg to 1 g), with yields of 83% and 85%, respectively. The efficiency of the reaction remained high when the nitrobenzene was substituted with an electron‐donating group (methyl group) and 4‐aminotoluene **2f** was obtained in 97% yield (Scheme 2).

**SCHEME 3:**
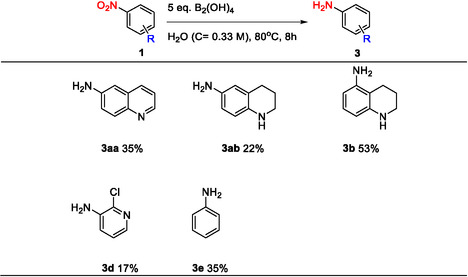
Scale‐up of the nitro reduction method reported by Chen et al. [21]. Isolated yields. Substrate scale: **1a** (1.5 g), **1b‐1e** (1.0 g).

In contrast, scaling‐up the method published by Chen et al. led to a significant decrease in yields. Moreover, scaling up of this method led to the reduction of the 6‐nitroquinoline pyridine ring which was not reported in the small‐scale study [21]. Upon a 9‐fold scale up, 6‐aminoquinoline **3aa** was obtained in a lower yield of 35% compared to the previously reported yield of 65%, while 1,2,3,4‐tetrahydroquinolin‐6‐amine **3ab** was isolated in a yield of 22%. Upon a 6‐fold scale‐up, the nitro reduction of 5‐nitroquinoline **1b** led to the expected reduction of both nitro group and pyridine ring, affording the 1,2,3,4‐tetrahydroquinolin‐5‐amine **3b** in a lower yield of 53% compared to the previously reported yield of 76%. 1,2,3,4‐tetrahydroquinolin‐6‐amine **3ab** and 1,2,3,4‐tetrahydroquinolin‐5‐amine **3b** were isolated in lower yields than expected due to challenging purifications. Finally, a dramatic decrease in yields was observed for 2‐chloropyridin‐3‐amine **3d** and aniline **3e**. 2‐Chloropyridin‐3‐amine **3d** was obtained in a significantly lower yield of 17% compared to the previously reported yield of 69%, after a 6‐fold scale‐up. Aniline **3e** was also obtained in a significant lower yield of 35% compared to the previously reported yield of 99%, after a 8‐fold scale‐up (Scheme 3).

**SCHEME 4:**
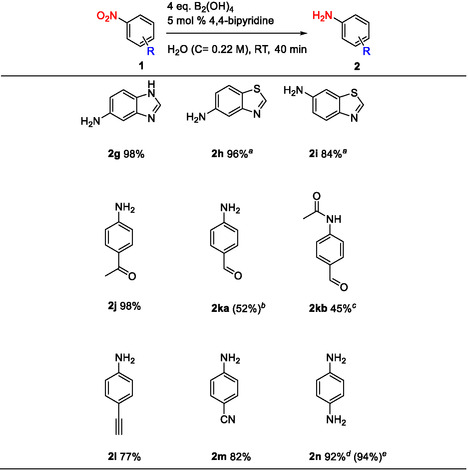
Substrate scope for the nitro reduction of various nitro compounds containing sensitive groups. Isolated yields. Substrate scale: **1g‐1n** (100 mg). ^a^Reaction carried out at 50°C instead of room temperature. ^b^Yield determined by ^1^H‐NMR using 1,3,5‐trimethoxybenzene as an internal standard. ^c^Isolated 4‐aminobenzaldehyde **2ka** as acetamide **2kb**. ^d^B_2_(OH)_4_ (8 eq.), 4,4′‐bipyridine (10 mol%), 40 min. ^e^B_2_(OH)_4_ (8 eq.), 4,4′‐bipyridine (5 mol%), 2 h.

To further investigate the chemoselectivity of the method, N‐heterocycles such as benzimidazole and benzothiazole scaffolds, which have been previously reported in the literature to undergo hydrogenation mediated by tetrahydroxydiboron in water, were selected [22]. ^1^H‐Benzo[d]imidazole‐5‐amine **2g** was obtained in excellent yield (98%) as mixture of tautomer's. The NMR characterization of **2g** was challenging due to the presence of a tautomeric equilibrium. At low concentration in DMSO‐*d_6_*, the ^1^H‐NMR chemical shifts correspond to a mixture of tautomer's (Figure S28), and the ^13^C NMR spectrum displays all expected carbon resonances (Figure S30). At higher concentration in DMSO‐*d_6_*, some ^13^C signals become broadened or are not observed (Figure S31). This behavior is likely related to the relatively high viscosity of DMSO‐*d_6_*. Notably, when the ^13^C NMR spectra are recorded in MeOH‐*d4* at high concentration, all expected resonances are clearly observable (Figure S31). Although, benzo[d]thiazole‐5‐amine **2h** was obtained in low yield (24%) when the reaction was carried out at room temperature, increasing the temperature to 50°C led to significantly improved yields for benzo[d]thiazole‐5‐amine **2h** and benzo[d]thiazole‐6‐amine **2i**, which were obtained in 96% and 84% yields, respectively (Scheme 4).

Our method proved to be chemoselective even with scaffolds containing easily reducible unsaturated bonds, such as carbonyl groups [10, 11, 12]. 4‐Aminoacetophenone **2j** was successfully isolated in excellent yield (96%). The nitro reduction of 4‐nitroacetophenone **1j** afforded a higher yield under the standard conditions compared to the reactions carried out at 50°C (86% yield) or using 5 equivalents of B_2_(OH)_4_ (61% yield). In case of 4‐aminobenzaldehyde **2ka**, the isolation of the product was challenging, as polymerization occurs immediately upon product formation. According to ^1^H‐NMR analysis, the reaction was incomplete and the reaction mixture still contained the starting material 4‐nitrobenzaldehyde **1k**. The desired product, 4‐aminobenzaldehyde **2ka** was obtained in 52% yield, with the reaction yield determined by ^1^H‐NMR using 1,3,5‐trimethoxybenzene as an internal standard (Figure S40). No formation of 4‐aminophenyl methanol was observed, indicating that the carbonyl group of the aldehyde is tolerated under these conditions, in contrast to other methods employing strong basic conditions in aqueous medium at elevated temperature [11]. Attempts to isolate 4‐aminobenzaldehyde as an HCl salt were unsuccessful, while the isolation of the aldehyde as bisulfite adduct following the method of Boucher et al., also failed [23]. To prevent polymerization, the reaction mixture was quenched with acetic anhydride, affording 4‐acetamidobenzaldehyde **2kb** in 45% isolated yield (Scheme 4).

Furthermore, reducible functional groups such as alkynyl and cyano groups are also tolerated under the standard reaction conditions. 4‐Aminophenylacetylene **2l** and 4‐aminobenzonitrile **2m** were obtained in 77% and 82% yields, respectively. In both cases, the reactions proceeded with clear conversions and no reduction of alkynyl (LC–MS analysis, Figure S2) or cyano groups (LC–MS analysis, Figure S3) was observed. Finally, both the nitro groups of 1,4‐dinitrobenzene **1n** were reduced and no formation of p‐nitroaniline was observed. Decreasing the amount of B_2_(OH)_4_ to 2 equivalents still favored the formation of the 1,4‐diaminobenzene **2n**, although traces of p‐nitroaniline were detected by mass spectrometry (LC–MS analysis, Figure S4). Under standard reaction conditions, 1,4‐diaminobenzene **2n** was formed with 40% conversion after 40 min and 45% conversion after 2 h, affording 46% isolated yield. Increasing the amount of B_2_(OH)_4_ to 8 equivalents led to 63% conversion after 40 min and complete conversion after 2 h, with 94% isolated yield. When both the amount of B_2_(OH)_4_) and 4,4′‐bipyridine were increased (8 instead of 4 equivalents and 10 mol% instead of 5 mol%, respectively), complete conversion was achieved after 40 min, affording 92% isolated yield of 1,4‐diaminobenzene **2n**. These results indicate that the quantity of reducing agent is crucial for the overall yield, whereas the organocatalyst primarily affects the reaction rate (LC–MS analysis, Figures S5 and S6).

**SCHEME 5:**
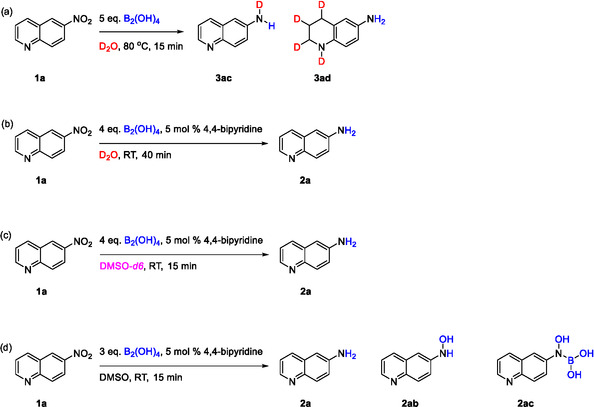
Mechanistic studies for the aromatic nitro reduction.

A series of experimental studies were conducted to investigate the reaction mechanism. First, the addition of the radical inhibitor (2,2,6,6 tetramethylpiperidin‐1‐yl)oxyl TEMPO did not affect the reaction, indicating that the reaction mechanism does not likely proceed via a radical pathway (LC–MS analysis, Figure S7). Moreover, deuterium labeling control experiments were performed using 6‐nitroquinoline **1a** and D_2_O as solvent. Previously, a possible mechanism for the nitro reduction with use of B_2_(OH)_4_ in H_2_O through a six‐membered ring coordinating the nitroaromatic with B_2_(OH)_4_ and H_2_O, was reported [21]. This mechanism was based on previous mechanistic studies for the reduction of aromatic nitro compounds to aromatic amines with B_2_pin_2_ in isopropanol [10]. In both cases, the ‐NH_2_ protons derived from the solvent (H_2_O or isopropanol) and not from the reducing agent B_2_(OH)_4_. To confirm this hypothesis, the reaction reported by Chen et al. was performed in D_2_O instead of H_2_O. Two main peaks of the deuterated product **3ac** and the over‐reduced deuterated byproduct **3ad** were observed. The formation of the deuterated product **3ac** confirms the previous hypothesis that the protons of ‐NH_2_ come from the solvent. On the contrary, the formation of deuterated byproduct **3ad** most likely follows a different mechanistic pathway, were the water acts as the hydrogen source for the reduction of the 6‐nitroquinoline pyridine ring (previously reported by Xia et al.) (Scheme 5a, Figure S8) [22].

Furthermore, a deuterium labeling control experiment was performed under the standard conditions (including the use of 4,4′‐bipyridine) using D_2_O as solvent. LC–MS analysis of the crude reaction mixture confirmed the formation of the desired nondeuterated product **2a**, indicating that the ‐NH_2_ hydrogen atoms exclusively originate from the B_2_(OH)_4_ and not from the solvent (Scheme 5b, LC–MS analysis Figure S9). As expected, when the crude reaction mixture was analysed by ^1^H NMR in D_2_O, the ‐NH_2_ protons were not observed due to rapid proton–deuterium exchange. (Scheme 5b, ^1^H NMR analysis, Figure S10). Upon dissolution of the crude mixture in CDCl_3_, the ‐NH_2_ protons became visible as separate broad singlets, reflecting the slower proton exchange and confirming the formation of the desired nondeuterated product **2a** (Scheme 5b, ^1^H NMR analysis Figure S11). These ^1^H NMR results further support the hypothesis that the reducing agent, rather than the solvent, serves as the ‐NH_2_ hydrogen source during the nitro reduction. It is likely that a 4,4′‐bipyridine‐B_2_(OH)_4_ complex is formed, which promotes proton transfer from the reducing agent B_2_(OH)_4_ and its formation is facilitated by H_2_O, as previously reported [19]. In the absence of 4,4′‐bipyridine, a similar complex between the pyridine ring of 6‐nitroquinoline **1a** and the B_2_(OH)_4_ is likely formed. This complex facilitates the reduction of the pyridine ring and again favours the proton transfer from the reducing agent B_2_(OH)_4_, leading to the amine group formation (Scheme 5a, **3ad**). The desired product **2a** was also obtained when DMSO‐*d6* was used as reaction solvent possibly indicating a similar mechanistic pathway in both cases (Scheme 5b,c, LC–MS analysis Figure S9, S12). Nevertheless, reducing the amount of B_2_(OH)_4_ to 3 equivalents in DMSO led to the observation of the intermediates **2ab** and **2ac**, supporting a possible mechanistic pathway (Scheme 5d, LC–MS analysis Figure S13). In contrast, no intermediates were detected when the reaction was conducted in H_2_O under the same conditions (LC–MS analysis Figure S14).

**SCHEME 6:**
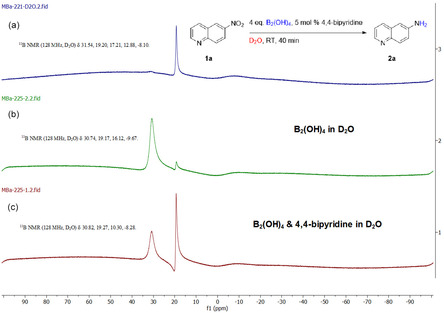
^11^B‐NMR control experiments.

Finally, ^11^B‐NMR and ^1^H‐NMR experiments were performed to gain insight into the 4,4′‐bipyridine‐B_2_(OH)_4_ complex. According to ^11^B‐NMR, the characteristic peaks of B_2_(OH)_4_ are a broad peak at 30.74 ppm along with a small broad peak at 19.17 ppm (Scheme 6b). A new complex was formed when 4,4′‐bipyridine was added to mixture of B_2_(OH)_4_ in D_2_O. The characteristic B peak of B_2_(OH)_4_ was shifted (30.82 ppm) and a sharp signal peak appeared at 19.27 ppm, indicating the generation of a new complex between the B_2_(OH)_4_ and the 4,4′‐bipyridine (Scheme 6c). Similarly, the two characteristic multiplets of 4,4′‐bipyridine ^1^H‐NMR ((δ 8.49–8.43 (m, 1H), 7.60–7.54 (m, 1H)) were shifted and appeared as singlets ((δ 8.55 (s, 4H), 7.70 (s, 4H)) upon addition of B_2_(OH)_4_ to a mixture of 4,4′‐bipyridine in D_2_O (Figure S15). ^11^B‐NMR analysis of the crude reaction mixture (Scheme 5b) confirmed the proposed 4,4′‐bipyridine‐B_2_(OH)_4_ complex formation indicated by a sharp resonance at 19.20 ppm (Scheme 6a).

**SCHEME 7:**
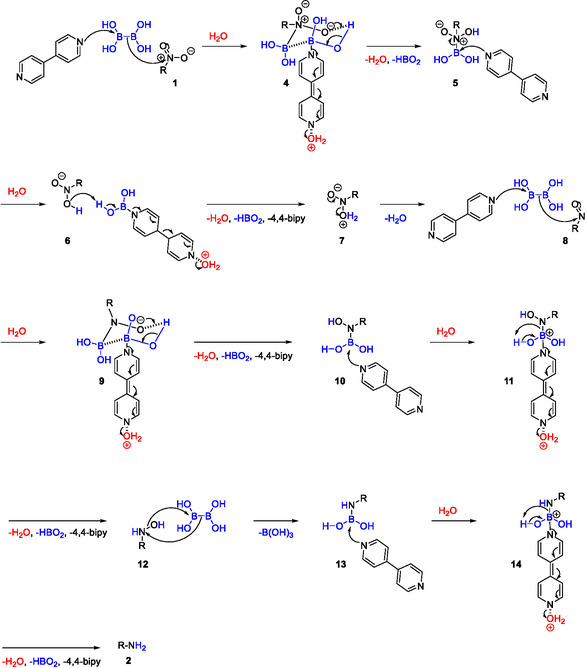
Plausible mechanistic pathway of nitro reduction.

Based on the above experimental results and the literature reports, a plausible mechanistic pathway is proposed. First, aromatic nitro substrate **1** is reduced to nitroso **8**. This is performed through a six‐membered ring transition state **4** where 4,4′‐bipyridine‐B_2_(OH)_4_ complex coordinates with nitroaromatic substrate and is stabilized by H_2_O molecule. Elimination of 4,4′‐bipyridine, HBO_2_ and H_2_O gives intermediate **5**. The nitroso intermediate **8** was formed after protonation of intermediate **6** which led to elimination of 4,4′‐bipyridine, HBO_2_ and H_2_O. A new six‐membered ring transition state **9** was formed followed again by elimination of 4,4′‐bipyridine, HBO_2_ and H_2_O to give intermediate **10**. The hydroxylamine **12** was formed through transition state **11** where 4,4′‐bipyridine‐B_2_(OH)_4_ complex coordinates with intermediate **10** and is stabilized by a H_2_O molecule. Elimination of 4,4′‐bipyridine, HBO_2_ and H_2_O gives hydroxylamine **12**. Finally, amine **15** is formed through transition state **14** where 4,4′‐bipyridine‐B_2_(OH)_4_ complex coordinates with intermediate **13** and is stabilized by a H_2_O molecule. Elimination of 4,4′‐bipyridine, HBO_2_ and H_2_O gives the desired amine product **2** (Scheme 7). It is crucial to emphasize the difference of the current proposed mechanism with previous reported mechanisms of nitro reductions that do not use 4,4′‐bipyridine. In previous reported mechanisms hydroxylamine **12** and amine **15** are formed after hydrolysis [10]. This explains why in absence of 4,4′‐bipyridine the ‐NH_2_ protons derive from the solvent (H_2_O or isopropanol) and not from the reducing agent B_2_(OH)_4_. In our proposed mechanism, the formation of hydroxylamine **12** and amine **15** goes through transition state **11** and **14** where 4,4′‐bipyridine‐B_2_(OH)_4_ complex coordinates with intermediates **10** and **13**, respectively. In that way, 4,4′‐bipyridine‐B_2_(OH)_4_ complex prevents the hydrolysis and favors the proton transfer from the reducing agent B_2_(OH)_4_ (Scheme 7). Finally, 4,4′‐bipyridine‐B_2_(OH)_4_ complex promotes the chemoselectivity by preventing the interaction of the reducing agent with either easily reducible groups or the pyridine ring of nitroquinolines and nitropyridines.

## 3 Conclusions

In summary, a metal‐free, fast, and chemoselective method has been developed for the reduction of aromatic nitro compounds to the corresponding aromatic amines. In this protocol, nitro reduction is performed in water at room temperature using B_2_(OH)_4_ as the reductant and 4,4′‐bipyridine as the organocatalyst. Unlike existing aqueous methodologies, this approach provides an efficient and mild process, minimizing selectivity issues such as over‐reduction of N‐heterocyclic rings or other easily reducible functional groups. A plausible reaction pathway has been proposed and supported by mechanistic studies, which reveal the crucial role of the 4,4′‐bipyridine–B_2_(OH)_4_ complex in controlling selectivity. Further research is ongoing to explore additional applications of this potentially greener methodology.

## 4 Experimental Section

### 4.1 Materials and Instruments

All reagents and solvents were purchased from commercial sources and were used without further purification. All the reactions were performed in 8 ml glass vials and the scale‐up reactions in 50 ml high pressure tubes.

Thin‐layer chromatography analysis was carried out with alumina silica plates (Merck Silica gel with 60 F254 indicators, precoated, thickness 0,25 mm) using UV light (254 or 365 nm). Purifications were performed with automatic flash chromatography on a Büchi Pure C‐815 flash chromatography system (normal phase) using silica gel (230–400 mesh) with Hawach Empty chromatography columns (solid load cartridges) or prepacked Reveleris silica.

All ^1^H,^13^C, and ^11^B NMR spectra were recorded at 400 and 100.6 MHz, respectively, on a Bruker Avance III HD 400 spectrometer equipped with 1H/BB z‐gradient probe (BBO, 5 mm). DMSO‐*d6* was used as solvent and tetramethylsilane was used as an internal chemical shift standard. All ^1^H and ^13^C spectra were acquired through the standard sequences available in the Bruker pulse program library and processes using MestReNova 15.0 NMR processing program. Proton and carbon chemical shifts are reported in parts per million (ppm, δ scale) and are referenced to the residual signal of the NMR solvent peak (DMSO‐*d6* at 2.50 ppm and 39.52 ppm, respectively). ^1^HNMR data are reported as chemical shift, multiplicity (s = singlet, d = doublet, t = triplet, q = quartet, and m = multiplet), constant coupling in Hertz (Hz) and integration.

LC−MS analysis was performed on an Agilent 1200 Series HPLC equipped with a Supelco Ascentic Express C18 column (3 cm × 4.6 mm, 2.7 µm fused‐core particles, 90 Å), Phenomenex Guard column (SecurityGuard Standard) and a UV‐DAD detector. The HPLC is coupled to an Agilent 1100 Series MS with electrospray ionisation (70 V) with a single quadrupole detector.

GC/MS analysis was performed on a Hewlett‐Packard 6890/5973N gas chromatograph/mass spectrometer equipped with an Phenomenex Zebron ZB‐5MSplus (30 m × 0.25 mm × 0.25 µm) column and quadrupole mass analyzer (Electron Ionization, 70 eV).

### 4.2 General Procedure for the Reduction of Nitro Compound

Nitroarene (100.0 mg, 0.5742 mmol, 1 eq.), 4,4′‐ bipyridine (4.48 mg, 0.0287 mmol, 0.05 eq.) and water (2.0 ml) were all added to a glass vial (8.0 mL) containing a magnetic stirring bar. B_2_(OH)_4_ (205.9 mg, 2.2967 mmol, 4 eq.) was added to the stirring solution and rinsed with water (0.6 ml). The reaction mixture was stirred at room temperature for 40 min. The reaction mixture was poured into a saturated NaHCO_3_ solution, the mixture was extracted with EtOAc and repeated three times. The combined organic layers were dried over anhydrous MgSO_4_ and filtered. The solvent was evaporated under reduced pressure. The crude product was purified by column chromatography on a silica gel to obtain the isolated yield.

### 4.3 Gram‐Scale Reaction for the Reduction of the Nitro Compound

6‐Aminoquinoline (1.5 g, 8.6128 mmol, 1 eq.), 4,4′‐bipyridine (67.26 mg, 0.4306 mmol, 0,05 eq.) and water (37.0 ml) were all added to a high pressure tube (50.0 ml) containing a magnetic stirring bar. B_2_(OH)_4_ (3.088 g, 34.4511 mmol, 4 eq.) was added to the stirring solution and rinsed with water (2.0 ml). The reaction mixture was stirred at room temperature for 40 min. The reaction mixture was poured into a saturated NaHCO_3_ solution, the mixture was extracted with EtOAc and repeated three times. The combined organic layers were dried over anhydrous MgSO_4_ and filtered. The solvent was evaporated under reduced pressure. The crude product was purified by column chromatography on a silica gel to obtain the isolated yield.

### 4.4 Scaling‐Up the Method Published in Synlett by Chen et al.

6‐Aminoquinoline (1.5 g, 8.6128 mmol, 1 eq.), B_2_(OH)_4_ (3.861 g, 43.0638 mmol, 5 eq.) and water (25.8 ml) were all added to a high pressure tube (50.0 ml) containing a magnetic stirring bar. The reaction mixture was stirred at 80°C for 8 h. The reaction mixture was poured into a saturated NaHCO_3_ solution, the mixture was extracted with EtOAc and repeated three times. The combined organic layers were dried over anhydrous MgSO_4_ and filtered. The solvent was evaporated under reduced pressure. The crude product was purified by column chromatography on a silica gel to obtain the isolated yield.

## 5 Characterization Data

### 5.1 6‐Aminoquinoline (2a)

Product was purified by flash chromatography on silica gel. Solvent A: DCM Solvent B: MeOH (0%−10%). Yellow solid (1.01 g, 81% yield). ^1^H‐NMR (400 MHz, DMSO‐*d6*): *δ* 8.52–8.41 (m, 1H), 7.97–7.88 (m, 1H), 7.70 (d, *J* = 8.9 Hz, 1H), 7.31–7.22 (m, 1H), 7.22–7.12 (m, 1H), 6.79 (d, *J* = 2.4 Hz, 1H), 5.59 (s, 2H)^.^. ^13^C‐NMR (100.6 MHz, DMSO): *δ* 147.48, 145.51, 142.52, 133.30, 130.29, 130.05, 122.08, 121.66, 105.23.

### 5.2 5‐Aminoquinoline (2b)

Product was isolated after work‐up without further purification. Orange solid (712.9 mg, 86% yield). ^1^H‐NMR (400 MHz, DMSO): *δ* 8.77 (dd, *J* = 4.2, 1.7 Hz, 1H), 8.57–8.49 (m, 1H), 7.42 (t, *J* = 8.0 Hz, 1H), 7.39–7.32 (m, 1H), 7.22–7.15 (m, 1H), 6.76–6.68 (m, 1H), 5.99 (s, 2H). ^13^C‐NMR (100.6 MHz, DMSO): *δ* 147.48, 145.51, 142.52, 133.30, 130.29, 130.05, 122.08, 121.66, 105.23.

### 5.3 8‐Aminoquinoline (2c)

Product was purified by flash chromatography on silica gel. Solvent A: DCM Solvent B: MeOH (0%−10%). Orange solid (680.0 mg, 82% yield). ^1^H‐NMR (400 MHz, DMSO): *δ* 8.73 (dd, *J* = 4.1, 1.7 Hz, 1H), 8.18 (dd, *J* = 8.3, 1.7 Hz, 1H), 7.48–7.43 (m, 1H), 7.30 (t, *J* = 7.8 Hz, 1H), 7.06 (dd, *J* = 8.1, 1.3 Hz, 1H), 6.88 (dd, *J* = 7.6, 1.3 Hz, 1H), 5.92 (s, 2H).^13^C‐NMR (100.6 MHz, DMSO): *δ* 147.42, 145.66, 137.87, 136.29, 129.01, 128.04, 121.89, 114.15, 109.11.

### 5.4 3‐Amino‐2‐chloropyridine (2d)

Work‐up: The reaction mixture was poured into a NaOH (aq.) solution, the mixture was extracted with DCM and repeated three times. The combined organic layers were dried over anhydrous MgSO4 and filtered. The solvent was evaporated under reduced pressure. The product was filtered with the aid of diethylether. Product was isolated after work‐up without further purification. Beige solid (603.3 mg, 74% yield). ^1^H‐NMR (400 MHz, DMSO): *δ* 7.58 (dd, *J* = 4.2, 2.0 Hz, 1H), 7.17 – 7.04 (m, 2H), 5.54 (s, 2H). ^13^C‐NMR (100.6 MHz, DMSO): *δ* 141.93, 136.54, 135.47, 124.19, 122.33.

### 5.5 Aniline (2e)

Work‐up: The reaction mixture was poured into a NaOH (aq.) solution, the mixture was extracted with DCM and repeated three times. The combined organic layers were dried over anhydrous MgSO4 and filtered. The solvent was evaporated under reduced pressure. Purification: Product was purified by flash chromatography on silica gel. Solvent A: Hexane Solvent B: EtOAc (0%−30%). Colorless oil (700.5 mg, contains 8,6% w/w EtOAc, 85% yield). ^1^H‐NMR (400 MHz, DMSO): *δ* 7.06–6.95 (m, 2H), 6.60–6.54 (m, 2H), 6.54–6.45 (m, 1H), 4.99 (s, 2H). ^13^C‐NMR (100.6 MHz, DMSO): *δ* 149.06, 129.27, 116.13, 114.36.

### 5.6 2‐Aminotoluene (2f)

Work‐up: The reaction mixture was poured into a NaOH (aq.) solution, the mixture was extracted with DCM and repeated three times. The combined organic layers were dried over anhydrous MgSO4 and filtered. The solvent was evaporated under reduced pressure. Product was isolated after work‐up without further purification. Beige solid (76.1 mg, 97% yield). ^1^H‐NMR (400 MHz, DMSO): *δ* 6.85–6.76 (m, 2H), 6.50–6.42 (m, 2H), 4.79 (s, 2H), 2.12 (s, 3H). ^13^C‐NMR (100.6 MHz, DMSO): *δ* 146.54, 129.69, 124.35, 114.48, 20.59.

### 5.7 ^1^H‐benzo[d]imidazole‐5‐amine (2g)

Work‐up: The reaction mixture was poured into a Na_2_CO_3_ (aq.) solution, the mixture was extracted with EtOAc and repeated three times. The combined organic layers were dried over anhydrous MgSO4 and filtered. The solvent was evaporated under reduced pressure. Purification: Product was purified by flash chromatography on silica gel. Solvent A: DCM Solvent B: MeOH (0%−15%). White solid (80.3 mg, 98% yield). ^1^H‐NMR (400 MHz, DMSO): *δ* 11.77 (s, 1H), 7.86 (s, 1H), 7.25 (d, *J* = 8.5 Hz, 1H), 6.67 (d, *J* = 2.1 Hz, 1H), 6.52 (dd, *J* = 8.5, 2.1 Hz, 1H), 4.87 (s, 2H). ^13^C‐NMR (100.6 MHz, DMSO): *δ* 145.20, 139.35, 135.74, 134.95, 119.39, 111.53, 95.15. ^1^H NMR (400 MHz, MeOD) *δ* 7.82 (s, 1H), 7.26 (d, *J* = 8.6 Hz, 1H), 6.82 (d, *J* = 2.1 Hz, 1H), 6.65 (dd, *J* = 8.6, 2.1 Hz, 1H). ^13^C NMR (100.6 MHz, MeOD) *δ* 143.36, 139.68, 137.08, 132.56, 115.98, 113.22, 99.12.

### 5.8 Benzo[d]thiazole‐5‐amine (2h)

The reaction mixture was stirred at 50°C for 40 min. Work‐up: The reaction mixture was poured into a Na_2_CO_3_ (aq.) solution, the mixture was extracted with EtOAc and repeated three times. The combined organic layers were dried over anhydrous MgSO4 and filtered. The solvent was evaporated under reduced pressure. Purification: Product was purified by flash chromatography on silica gel. Solvent A: Hexane Solvent B: EtOAc (0%−50%). White solid (80.1 mg, 96% yield). ^1^H‐NMR (400 MHz, DMSO): *δ* 9.17 (s, 1H), 7.73 (d, *J* = 8.6 Hz, 1H), 7.22–7.16 (m, 1H), 6.85–6.78 (m, 1H), 5.30 (s, 2H). ^13^C‐NMR (100.6 MHz, DMSO): *δ* 155.76, 155.27, 148.37, 122.44, 120.78, 115.54, 106.33.

### 5.9 Benzo[d]thiazole‐6‐amine (2i)

The reaction mixture was stirred at 50°C for 40 min. Work‐up: The reaction mixture was poured into a Na_2_CO_3_ (aq.) solution, the mixture was extracted with EtOAc and repeated three times. The combined organic layers were dried over anhydrous MgSO4 and filtered. The solvent was evaporated under reduced pressure. Purification: Product was purified by flash chromatography on silica gel. Solvent A: Hexane Solvent B: EtOAc (25%−100%). White solid (70.0 mg, 84% yield). ^1^H‐NMR (400 MHz, DMSO): *δ* 8.88 (s, 1H), 7.71 (d, *J* = 8.7 Hz, 1H), 7.12 (d, *J* = 2.2 Hz, 1H), 6.80 (dd, *J* = 8.7, 2.2 Hz, 1H), 5.40 (s, 2H). ^13^C‐NMR (100.6 MHz, DMSO): *δ* 149.59, 147.79, 145.25, 135.67, 123.55, 115.37, 104.26.

### 5.10 4‐Aminoacetophenone (2j)

Work‐up: The reaction mixture was poured into a NaOH (aq.) solution, the mixture was extracted with DCM and repeated three times. The combined organic layers were dried over anhydrous MgSO4 and filtered. The solvent was evaporated under reduced pressure. Purification: Product was purified by flash chromatography on silica gel. Solvent A: Hexane Solvent B: EtOAc (20%−50%). White solid (79.8 mg, 98% yield). ^1^H‐NMR (400 MHz, DMSO): *δ* 87.69–7.64 (m, 2H), 6.58–6.52 (m, 2H), 6.05 (s, 2H), 2.38 (s, 3H). ^13^C‐NMR (100.6 MHz, DMSO): *δ* 195.37, 154.07, 131.02, 125.32, 112.92, 26.30.

### 5.11 4‐Aminoacetophenone (2ka)

After the reaction was stirred at room temperature for 40 min, the internal standard 1,3,5‐Trimethoxybenzene (51 mg) was added to the reaction mixture. Sample from the reaction mixture was dissolved in DMSO‐d6 solvent and analyzed by a NMR spectrometer to obtain the NMR yield. (mmol_product_ = (Intergration_product_/numberH_product_) × (numberH_standard_/Integration_standard_) × mmol_standard_ = 41.8 mg) (52% yield). ^1^H‐NMR (400 MHz, DMSO): *δ* 9.56 (s, 1H), 7.59–7.52 (m, 2H), 6.64–6.61 (m, 2H), 6.30 (s, 2H).

### 5.12 4‐Acetamidobenzaldehyde (2 kb)

After the reaction was stirred at room temperature for 40 min, acetic anhydride (0.5 ml, 5.2938 mmol, 8 eq.) was added to the reaction mixture. Work‐up: The reaction mixture was dissolved in MeOH (3,0 ml) and poured into a NaHSO_3_ (aq) solution (7.0 ml), the mixture was extracted with EtOAc and repeated three times. The water layer was basified with 2 M NaOH (aq.) solution and extracted with EtOAc and repeated three times. The combined organic layers were dried over anhydrous MgSO_4_ and filtered. The solvent was evaporated under reduced pressure. Purification: Product was purified by flash chromatography on silica gel. Solvent A: Hexane Solvent B: EtOAc (0%−5%). Yellow solid. (50.0 mg, contains 3.1% w/w EtOAc, 45% yield). ^1^H NMR (400 MHz, DMSO) *δ* 10.38 (s, 1H), 9.87 (s, 1H), 7.89–7.82 (m, 2H), 7.82–7.73 (m, 2H). ^13^C NMR (100.6 MHz, DMSO) δ 192.03, 169.61, 145.31, 131.55, 131.34, 118.99, 24.72.

### 5.13 4‐Aminophenylacetylene (2l)

Work‐up: The reaction mixture was poured into a Na_2_CO_3_ (aq.) solution, the mixture was extracted with EtOAc and repeated three times. The combined organic layers were dried over anhydrous MgSO4 and filtered. The solvent was evaporated under reduced pressure. Purification: Product was purified by flash chromatography on silica gel. Solvent A Hexane Solvent B: EtOAc (0%−20%). White solid. (60.3 mg, 77% yield). ^1^H NMR (400 MHz, DMSO) *δ* 7.09–6.96 (m, 2H), 6.47–6.35 (m, 2H), 5.44 (s, 2H), 3.71 (s, 1H). ^13^C NMR (100.6 MHz, DMSO) *δ* 149.97, 133.23, 113.93, 108.10, 85.60, 77.67.

### 5.14 4‐Aminobenzonitrile (2m)

Work‐up: The reaction mixture was poured into a NaOH (aq.) solution, the mixture was extracted with EtOAc and repeated three times. The combined organic layers were dried over anhydrous MgSO4 and filtered. The solvent was evaporated under reduced pressure. Purification: Product was purified by flash chromatography on silica gel. Solvent A: Hexane Solvent B: EtOAc (0%−30%). White solid (65.1 mg, contains 0.8% w/w MeOH, 82% yield). ^1^H NMR (400 MHz, DMSO) *δ* 7.45–7.28 (m, 2H), 6.69–6.52 (m, 2H), 6.13 (s, 2H). ^13^C NMR (100.6 MHz, DMSO) *δ* 153.48, 133.91, 121.14, 113.91, 95.98.

### 5.15 1,4‐Diaminobenzene (2n)

More equivalents of 4,4′‐ bipyridine (9.29 mg, 0.0595 mmol, 0.1 eq.) and B_2_(OH)_4_ (426.6 mg, 4.7588 mmol, 8 eq.) were used. Work‐up: The reaction mixture was poured into a NaOH (aq.) solution, the mixture was extracted with EtOAc and repeated three times. The combined organic layers were dried over anhydrous MgSO4 and filtered. The solvent was evaporated under reduced pressure. Purification: Product was purified by flash chromatography on silica gel. Solvent A: DCM Solvent B: MeOH (0%−6%). Off‐white solid (58.9 mg, 92% yield). ^1^H NMR (400 MHz, DMSO) *δ* 6.35 (s, 4H), 4.21 (s, 4H). ^13^C NMR (100.6 MHz, DMSO) *δ* 139.34, 115.89.

### 5.16 6‐Aminoquinoline (3aa)

Crude was purified twice by flash chromatography on silica gel in order to obtain pure product. Solvent A: DCM Solvent B: MeOH (0%−10%) and Solvent A: Hexane Solvent B: EtOAc (0%−65%). Dark brown solid (434.1 mg, 34% yield). ^1^H‐NMR (400 MHz, DMSO): *δ* 8.50–8.43 (m, 1H), 7.97–7.85 (m, 1H), 7.70 (d, *J* = 8.9 Hz, 1H), 7.31–7.22 (m, 1H), 7.22–7.12 (m, 1H), 6.79 (d, *J* = 2.5 Hz, 1H), 5.59 (s, 2H)^.^. ^13^C‐NMR (100.6 MHz, DMSO): *δ* 147.48, 145.51, 142.52, 133.29, 130.29, 130.05, 122.08, 121.66, 105.24.

### 5.17 1,2,3,4‐tetrahydroquinolin‐6‐amine (3ab)

Crude was purified by flash chromatography on silica gel. Solvent A: DCM Solvent B: MeOH (0%−10%). Diethylether was added to the crude obtained after purification and the filtrate was concentrated under reduced pressure to obtain product. Dark brown solid (320.0 mg, contains 13% w/w 6‐Aminoquinoline, 22% yield). ^1^H‐NMR (400 MHz, DMSO): *δ* 6.25–6.19 (m, 2H), 6.19–6.15 (m, 1H), 4.70 (s, 1H), 4.10 (s, 2H), 3.09–3.01 (m, 2H), 2.55 (t, *J* = 6.5 Hz, 2H), 1.74 (p, *J* = 6.2 Hz, 2H). ^13^C‐NMR (100.6 MHz, DMSO): *δ* 139.02, 137.16, 121.71, 115.95, 115.57, 114.08, 42.04, 27.23, 22.89.

### 5.18 1,2,3,4‐tetrahydroquinolin‐5‐amine (3b)

Crude was purified twice by flash chromatography on silica gel in order to obtain pure product. Solvent A: Hexane Solvent B: EtOAc (0%−50%) and Solvent A: DCM Solvent B: MeOH (0%−10%). Beige solid (450.0 mg, 53% yield). ^1^H‐NMR (400 MHz, DMSO): *δ* 6.55 (t, *J* = 7.8 Hz, 1H), 5.85 (dd, *J* = 7.7, 1.2 Hz, 1H), 5.74 (dd, *J* = 7.9, 1.2 Hz, 1H), 5.22 (t, *J* = 2.4 Hz, 1H), 4.46 (s, 2H), 3.10–3.01 (m, 2H), 2.33 (t, *J* = 6.6 Hz, 2H), 1.84–1.75 (m, 2H). ^13^C‐NMR (100.6 MHz, DMSO): *δ* 146.73, 146.45, 126.54, 105.15, 103.60, 103.25, 40.94, 22.51, 21.82.

### 5.19 3‐Amino‐2‐chloropyridine (3d)

Product was purified by flash chromatography on silica gel. Solvent A: Hexane Solvent B: EtOAc (0%−50%). Off‐white solid (140.0 mg, 17% yield). ^1^H‐NMR (400 MHz, DMSO): *δ* 7.58 (dd, *J* = 4.1, 2.1 Hz, 1H), 7.15–7.06 (m, 2H), 5.56 (s, 2H). ^13^C‐NMR (100.6 MHz, DMSO): *δ* 141.94, 136.51, 135.44, 124.21, 122.31.

### 5.20 Aniline (3e)

Work‐up: The reaction mixture was poured into a NaOH (aq.) solution, the mixture was extracted with DCM and repeated three times. The combined organic layers were dried over anhydrous MgSO4 and filtered. The solvent was evaporated under reduced pressure. Purification: Product was purified by flash chromatography on silica gel. Solvent A: Hexane Solvent B: EtOAc (0%−30%). Colorless oil (264.2 mg, 35% yield). ^1^H‐NMR (400 MHz, DMSO): *δ* 7.07–6.94 (m, 2H), 6.61–6.53 (m, 2H), 6.53–6.45 (m, 1H), 4.99 (s, 2H). ^13^C‐NMR (100.6 MHz, DMSO): *δ* 149.06, 129.26, 116.12, 114.35.

## Author Contributions

**Maria Batzaki**: investigation, writing – original draft. **Thomas S. A. Heugebaert**: validation, writing – review and editing. **Christian V. Stevens**: writing – review and editing, conceptualization, funding acquisition.

## Supporting Information

Additional supporting information can be found online in the Supporting Information section.

## Funding

This study was supported by Horizon MSCA 2021 DN 01‐01 (101073089).

## Conflicts of Interest

The authors declare no conflicts of interest.

## Supporting information

Supplementary Material

## Data Availability

The data that support the findings of this study are available on request from the corresponding author. The data are not publicly available due to privacy or ethical restrictions.
