# Supplementary material for: Metal‐Free, Chemoselective Reduction of Aromatic Nitro Compounds in Water at Room Temperature
Source: ChemistryOpen. 2026 May 14;15(5):e70219. doi: 10.1002/open.70219 (PMC13176622; doi:10.1002/open.70219)
Supplement: Supplementary file 1 — Supplementary Material [file OPEN-15-e70219-s001.pdf]

Supporting Information

for the article entitled:

Meal-Free, Chemoselective Reduction of Aromatic Nitro Compounds in Water at Room Temperature

By

Maria Batzaki, Thomas S. A. Heugebaert, Christian V. Stevens*

Department of Green Chemistry and Technology, Faculty of Bioscience Engineering, Ghent University, Ghent, Belgium

1. Calibration Curve

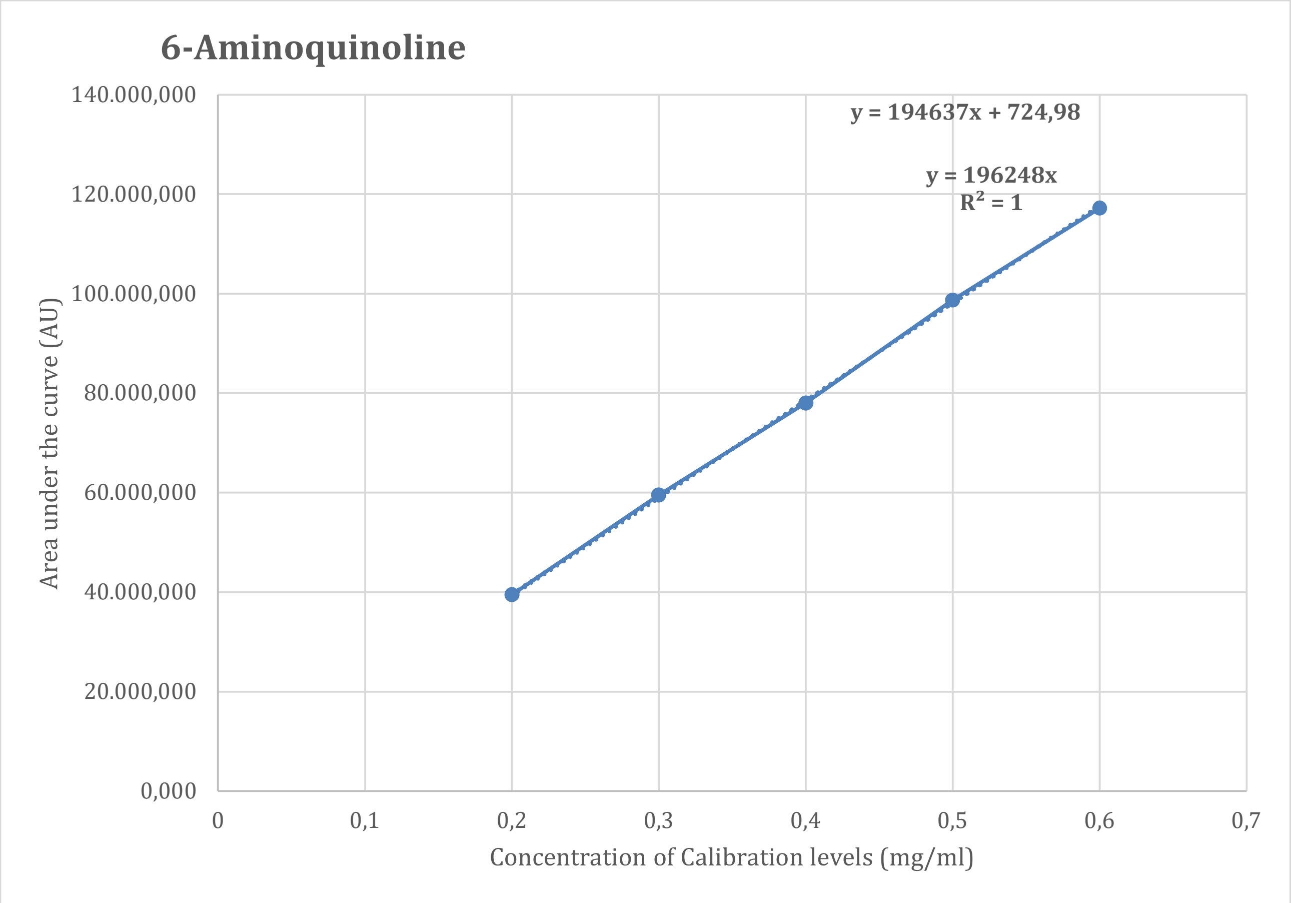

Figure S1 LC-MS calibration curve of 6-Aminoquinoline **2a**.

**concentration (mg/ml)**	volume sample (ml)	mass sample (mg)	**Polair Area LC-MS (254)**
**0.6**	0.48	0.48	117223.700
**0.5**	0.40	0.40	98678.909
**0.4**	0.32	0.32	77992.962
**0.3**	0.24	0.24	59515.967
**0.2**	0.16	0.16	39486.808

A stock solution of 25mg 6-Aminoquinoline in 25ml MeCN/H2O 1:1 was prepared.

Five samples were prepared:

MBa-055-06 0.6mg/ml (480µl stock solution + 320µl MeCN/H2O 1:1)

MBa-055-05 0.5mg/ml (400µl stock solution + 400µl MeCN/H2O 1:1)

MBa-055-04 0.4mg/ml (320µl stock solution + 480µl MeCN/H2O 1:1)

MBa-055-03 0.3mg/ml (240µl stock solution + 560µl MeCN/H2O 1:1)

MBa-055-02 0.2mg/ml (160µl stock solution + 640µl MeCN/H2O 1:1)

2. LC-MS analysis of reaction mixtures

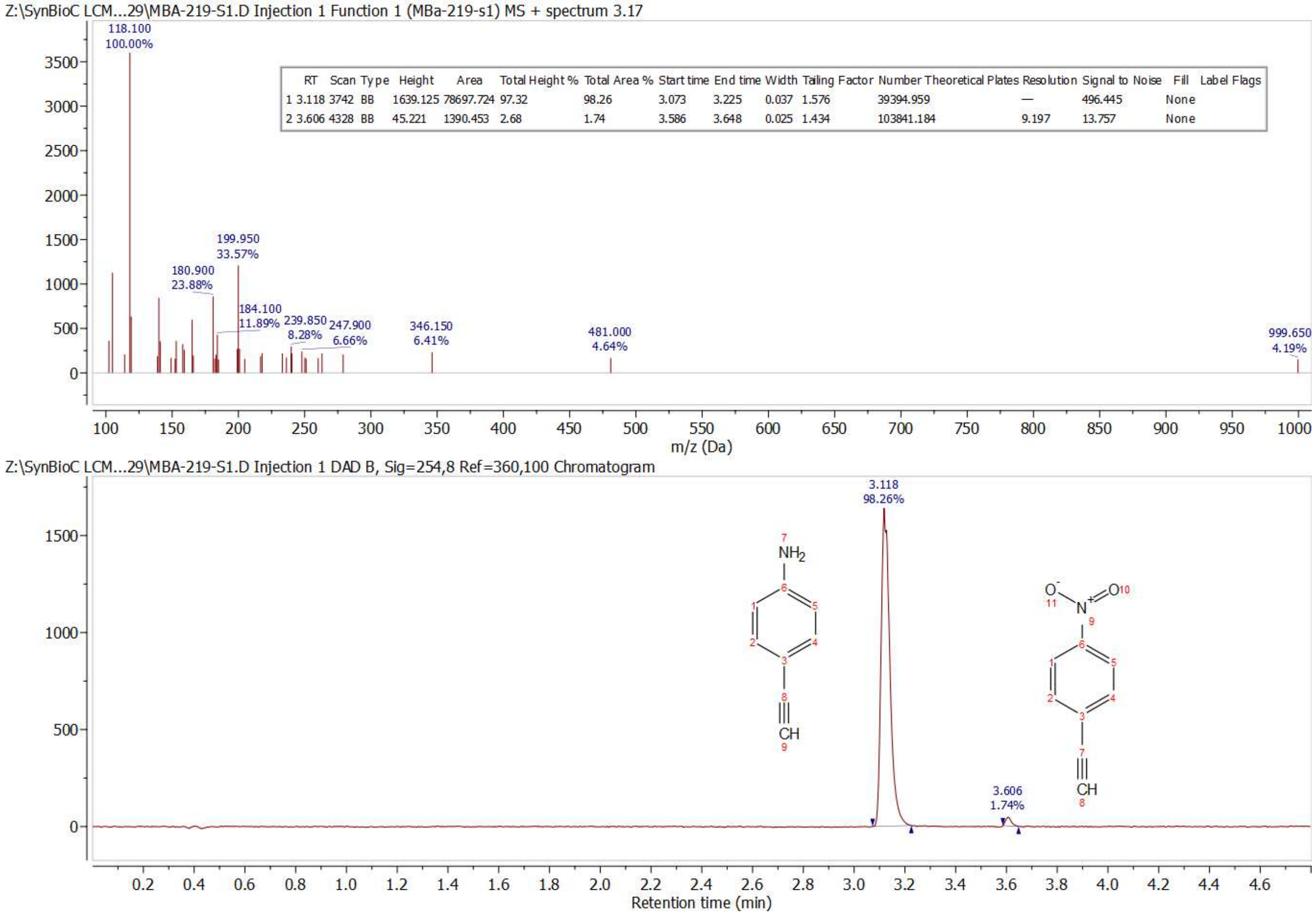

	RT	Scan	Type	Height	Area	Total Height %	Total Area %	Start time	End time	Width	Tailing Factor	Number Theoretical Plates	Resolution	Signal to Noise	Fill	Label Flags
1	3.118	3742	BB	1639.125	78697.724	97.32	98.26	3.073	3.225	0.037	1.576	39394.959	—	496.445	None	
2	3.606	4328	BB	45.221	1390.453	2.68	1.74	3.586	3.648	0.025	1.434	103841.184	9.197	13.757	None	

Figure S2 LC-MS analysis reaction mixture **2l**.

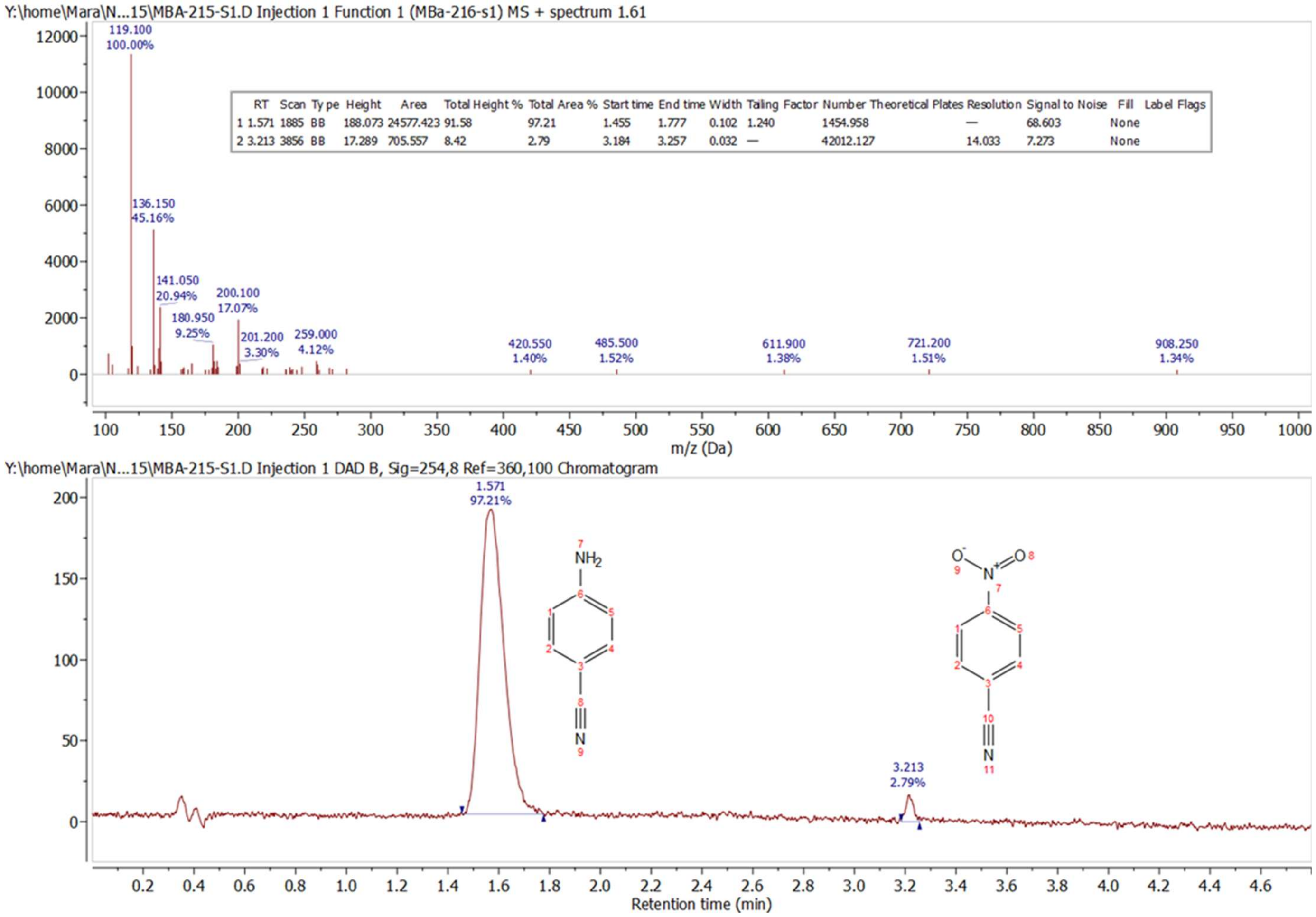

	RT	Scan	Type	Height	Area	Total Height %	Total Area %	Start time	End time	Width	Tailing Factor	Number Theoretical Plates	Resolution	Signal to Noise	Fill	Label Flags
1	1.571	1885	BB	188.073	24577.423	91.58	97.21	1.455	1.777	0.102	1.240	1454.958	—	68.603	None	
2	3.213	3856	BB	17.289	705.557	8.42	2.79	3.184	3.257	0.032	—	42012.127	14.033	7.273	None	

Figure S3 LC-MS analysis reaction mixture **2m**.

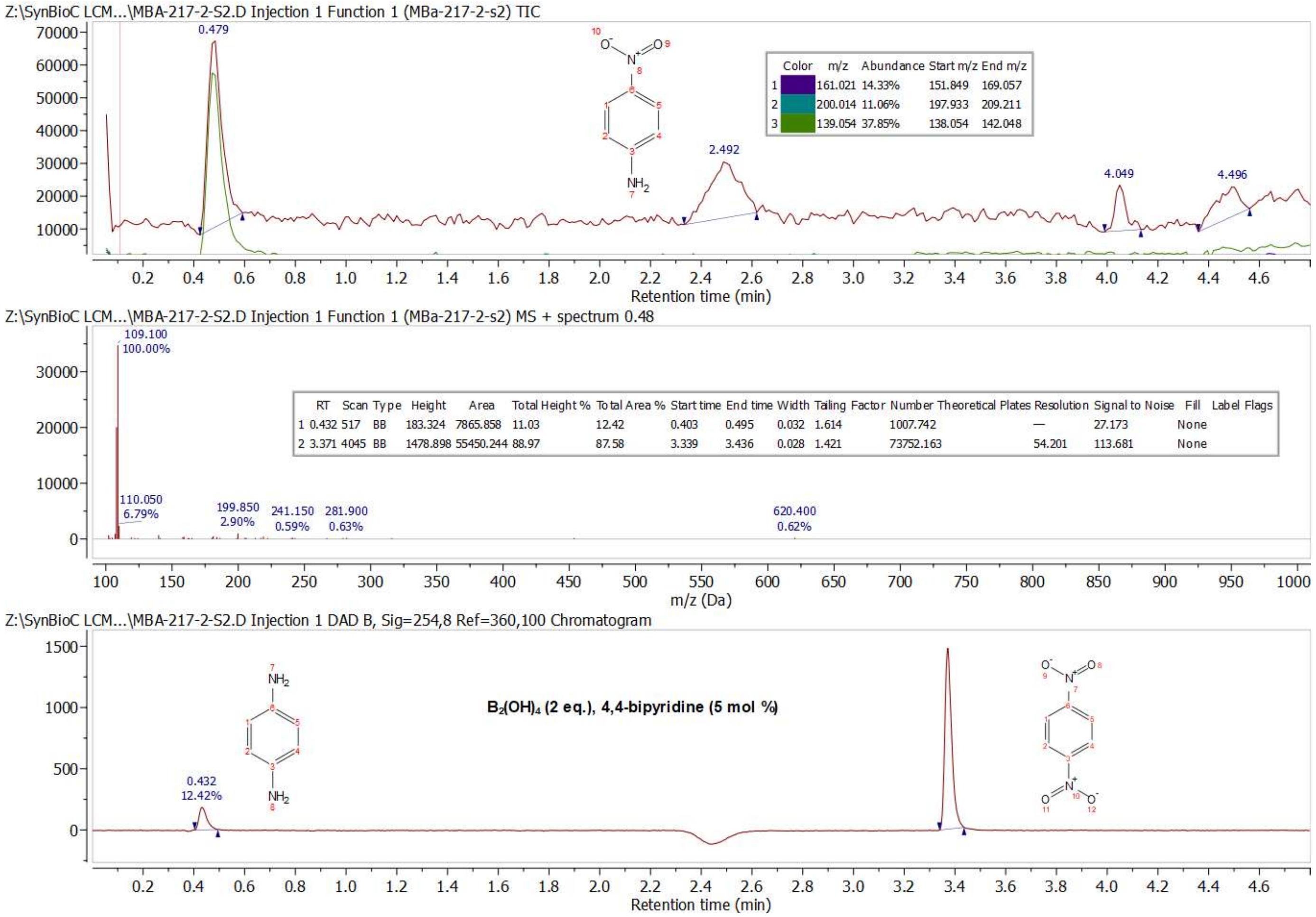

Figure 4 LC-MS analysis reaction mixture **2n** after 40 min.

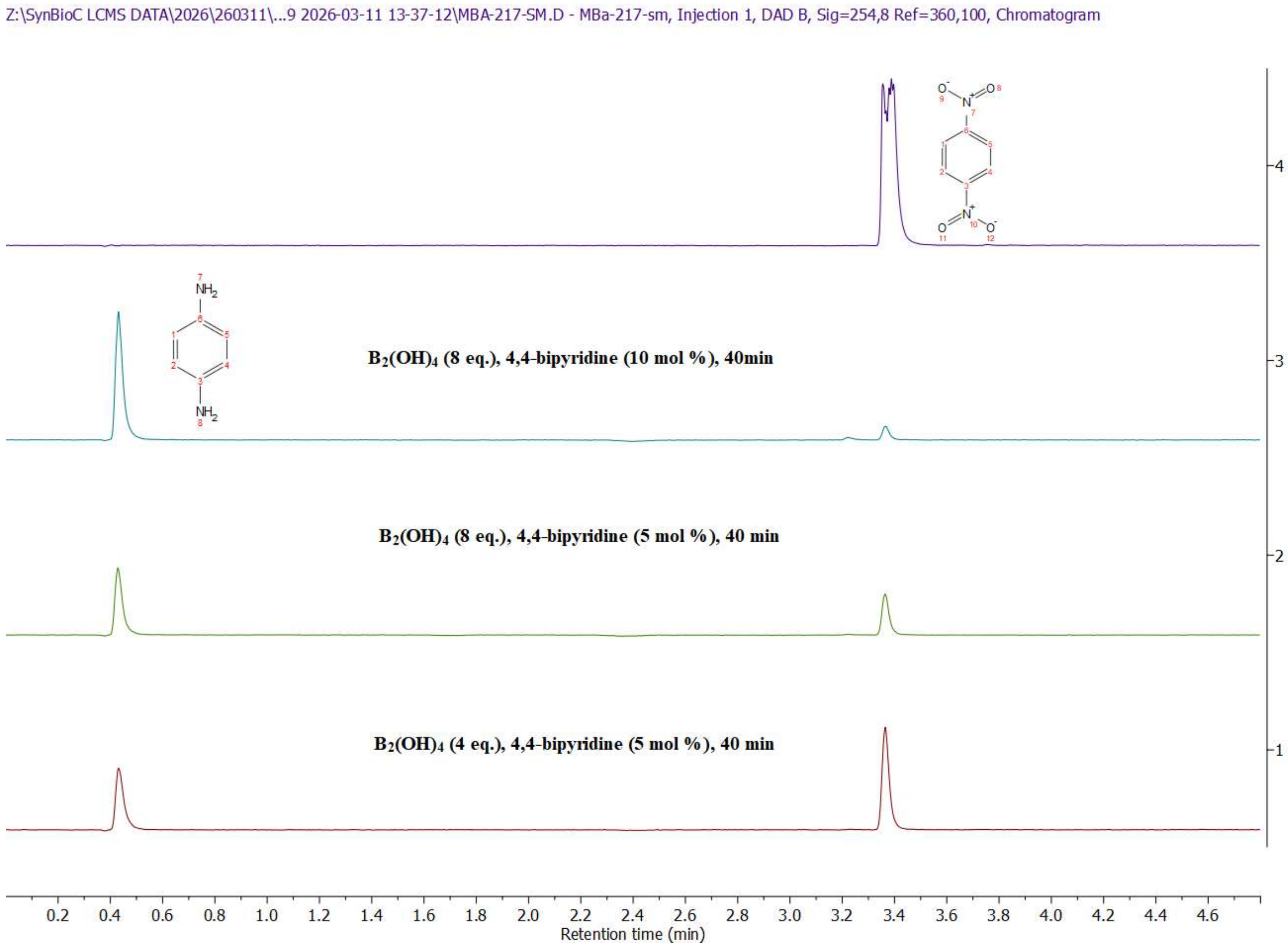

Figure S5 LC-MS analysis reaction mixtures **2n** after 40 min.

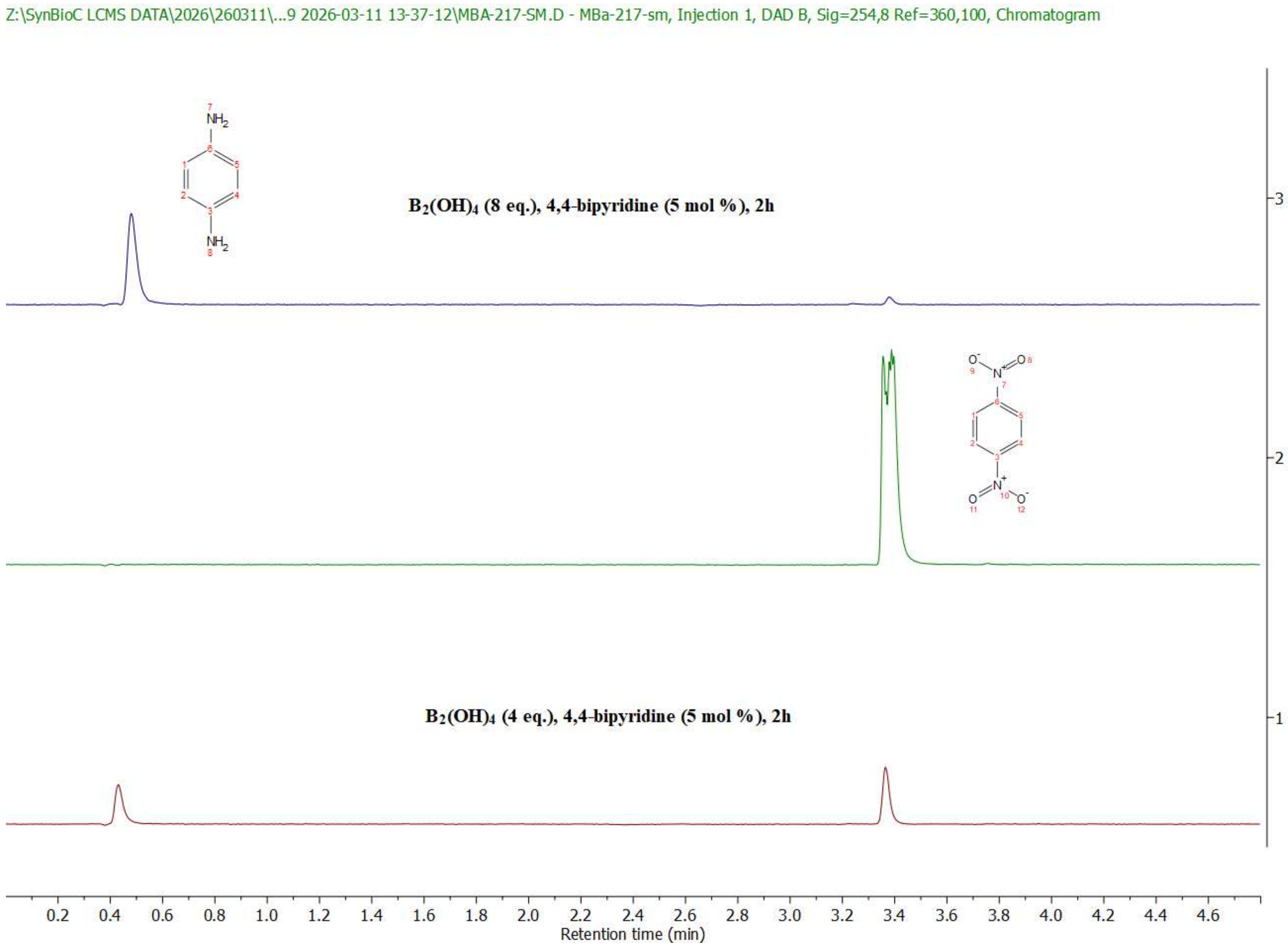

Figure S6 LC-MS analysis reaction mixtures **2n** after 2h.

3. Mechanistic studies

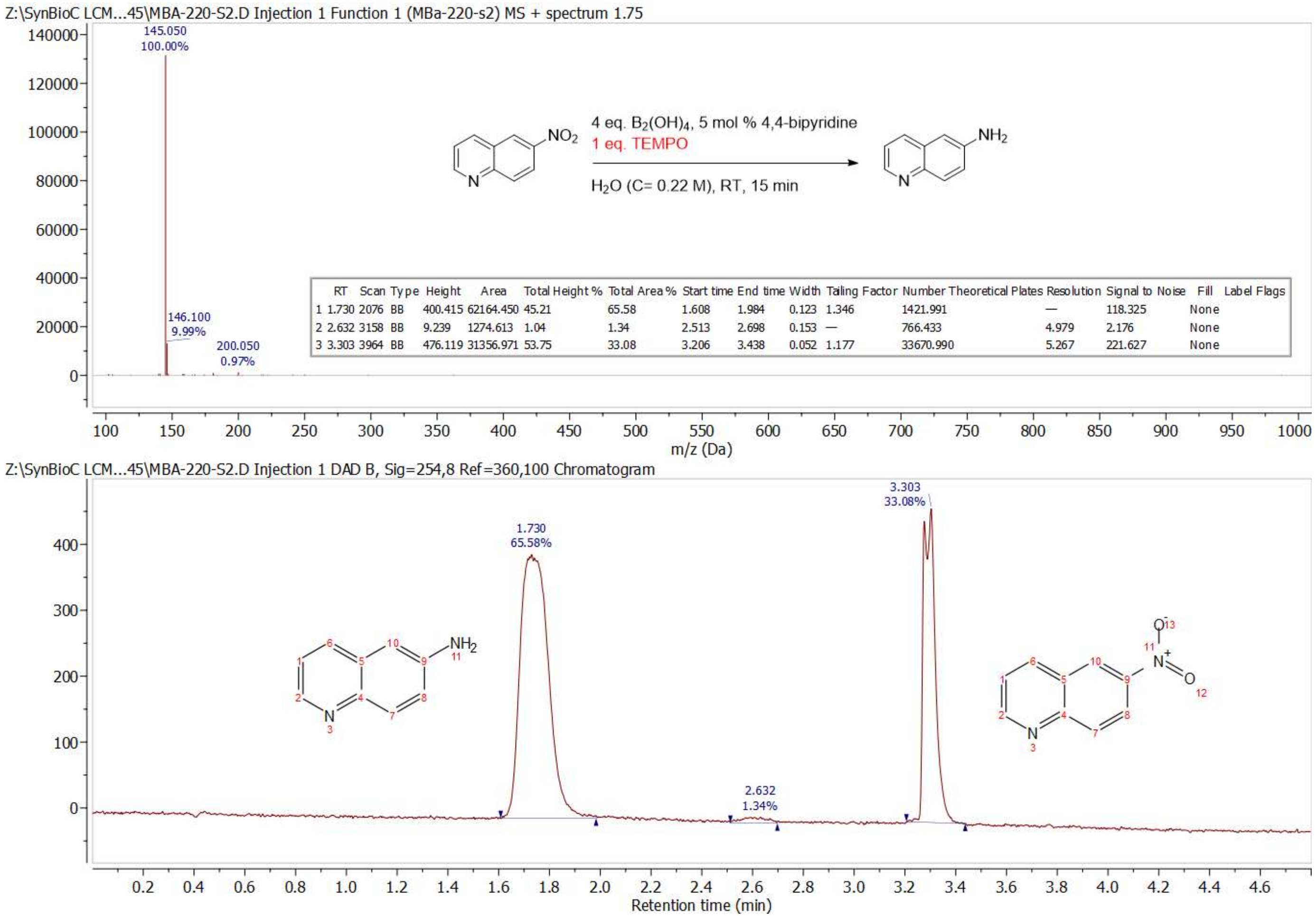

	RT	Scan	Type	Height	Area	Total Height %	Total Area %	Start time	End time	Width	Tailing Factor	Number Theoretical Plates	Resolution	Signal to Noise	Fill	Label Flags
1	1.730	2076	BB	400.415	62164.450	45.21	65.58	1.608	1.984	0.123	1.346	1421.991	—	118.325	None	
2	2.632	3158	BB	9.239	1274.613	1.04	1.34	2.513	2.698	0.153	—	766.433	4.979	2.176	None	
3	3.303	3964	BB	476.119	31356.971	53.75	33.08	3.206	3.438	0.052	1.177	33670.990	5.267	221.627	None	

Figure S7 LC-MS analysis of crude reaction mixture. Control experiment with addition of radical inhibitor TEMPO.

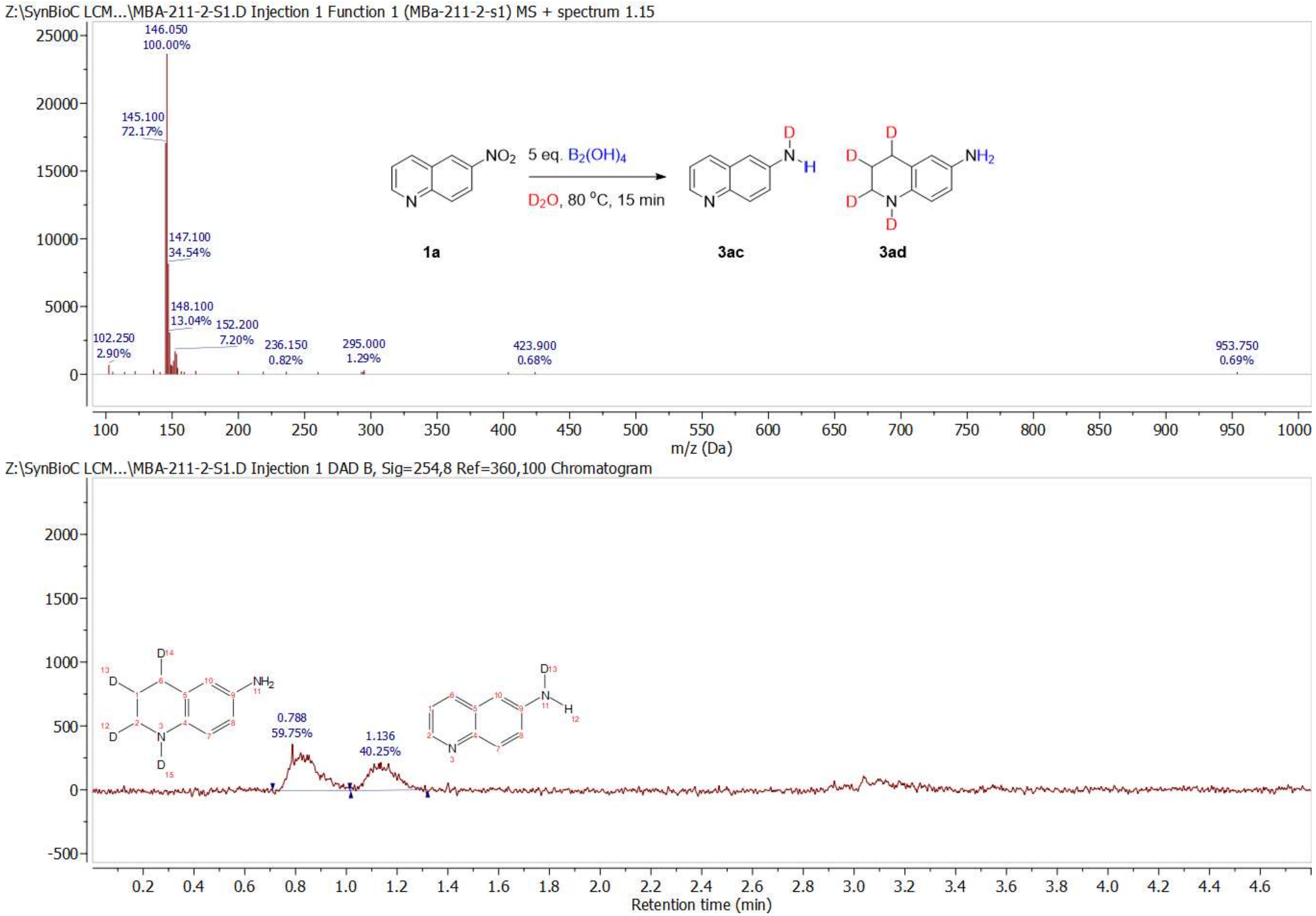

Figure 8 LC-MS analysis of crude reaction mixture. Deuterium labeling control experiments for the reaction reported by Chen et al.

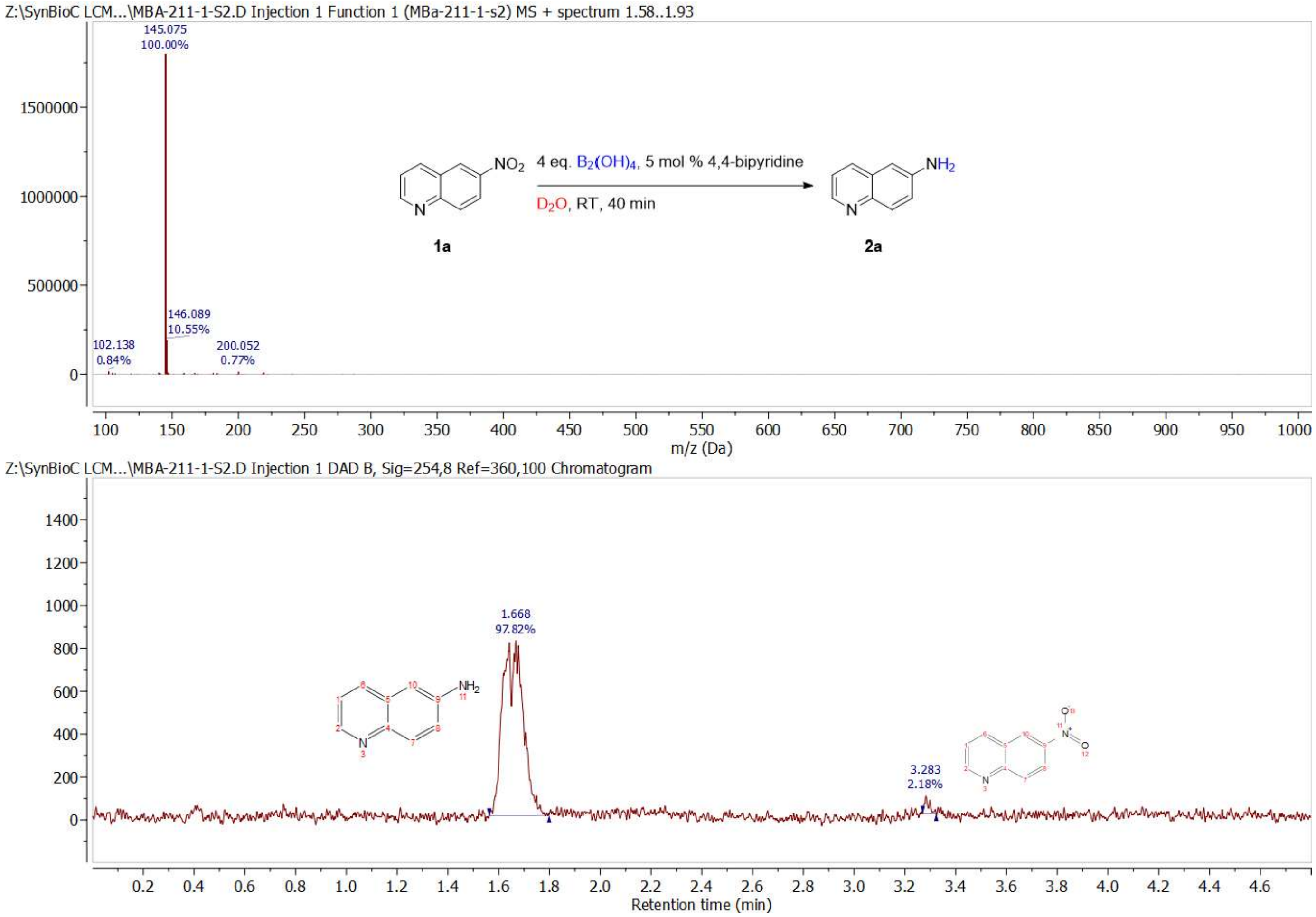

Figure 9 LC-MS analysis of crude reaction mixture. Deuterium labeling control experiments for the nitro reduction of the current study with D_2O as solvent.

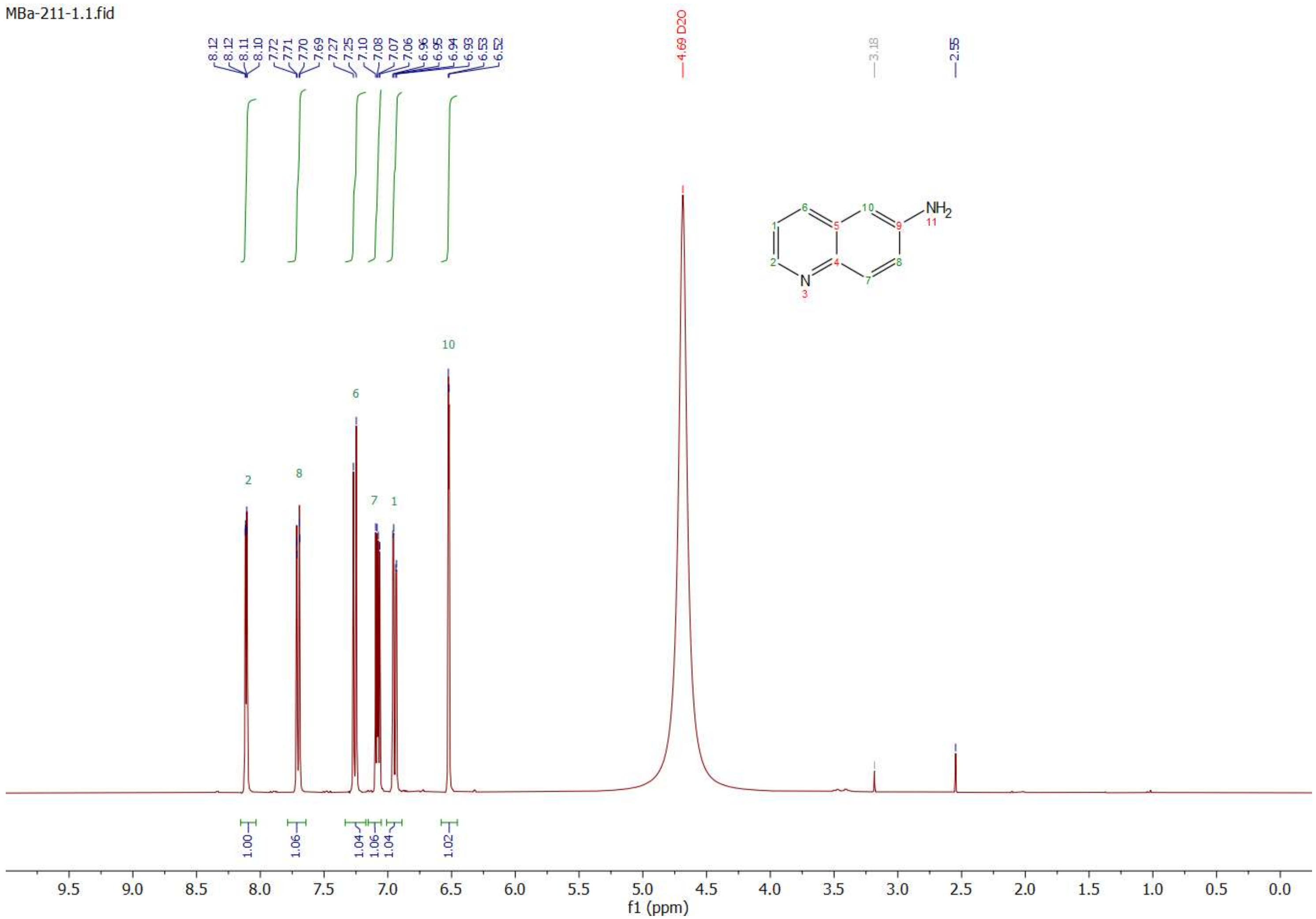

Figure S10 1H-NMR analysis of crude reaction mixture. Deuterium labeling control experiments for the nitro reduction of the current study with D_2O as solvent.

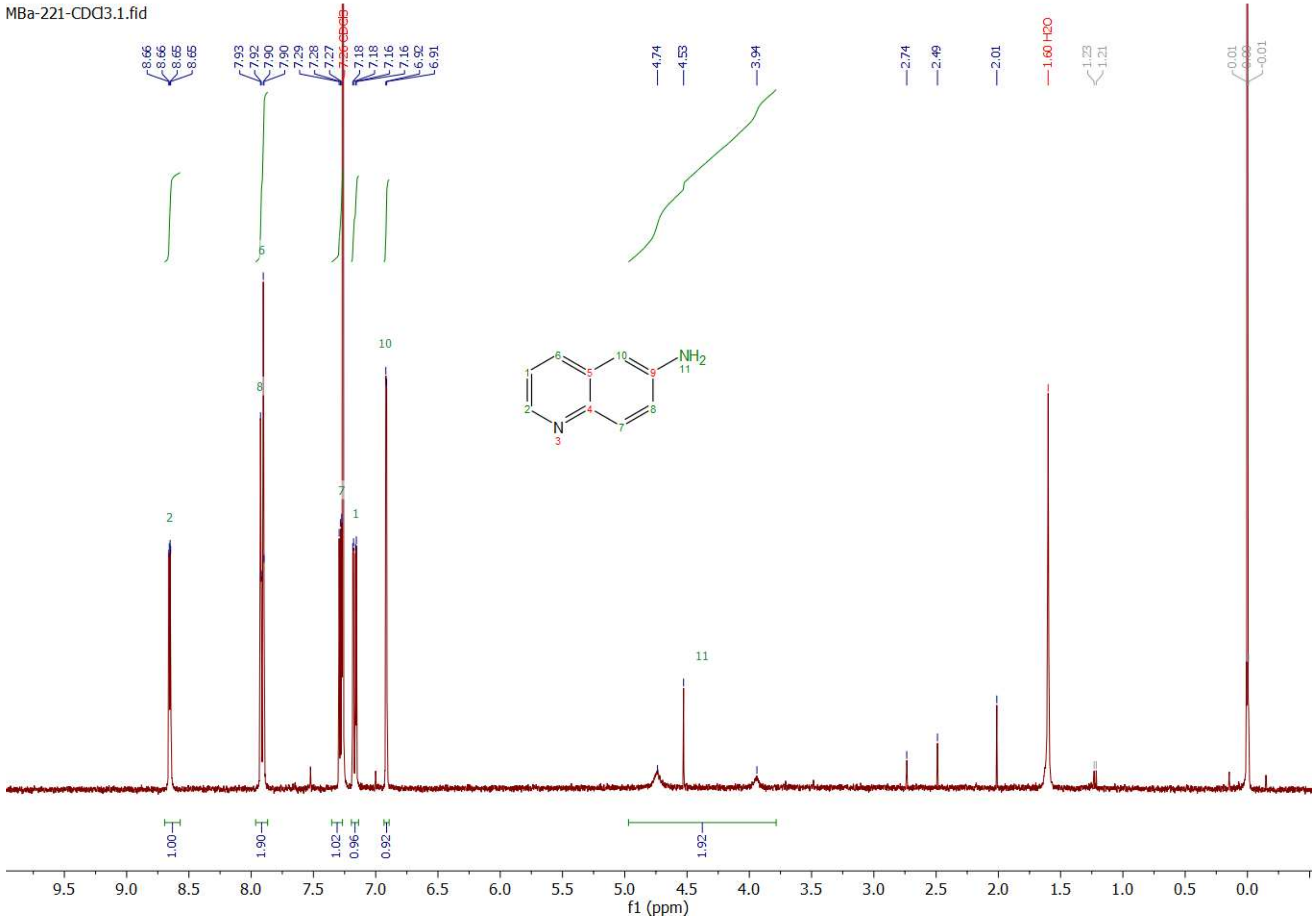

Figure S11 1H-NMR analysis of crude reaction mixture in $CDCl_3$. Deuterium labeling control experiments for the nitro reduction of the current study with D_2O as solvent.

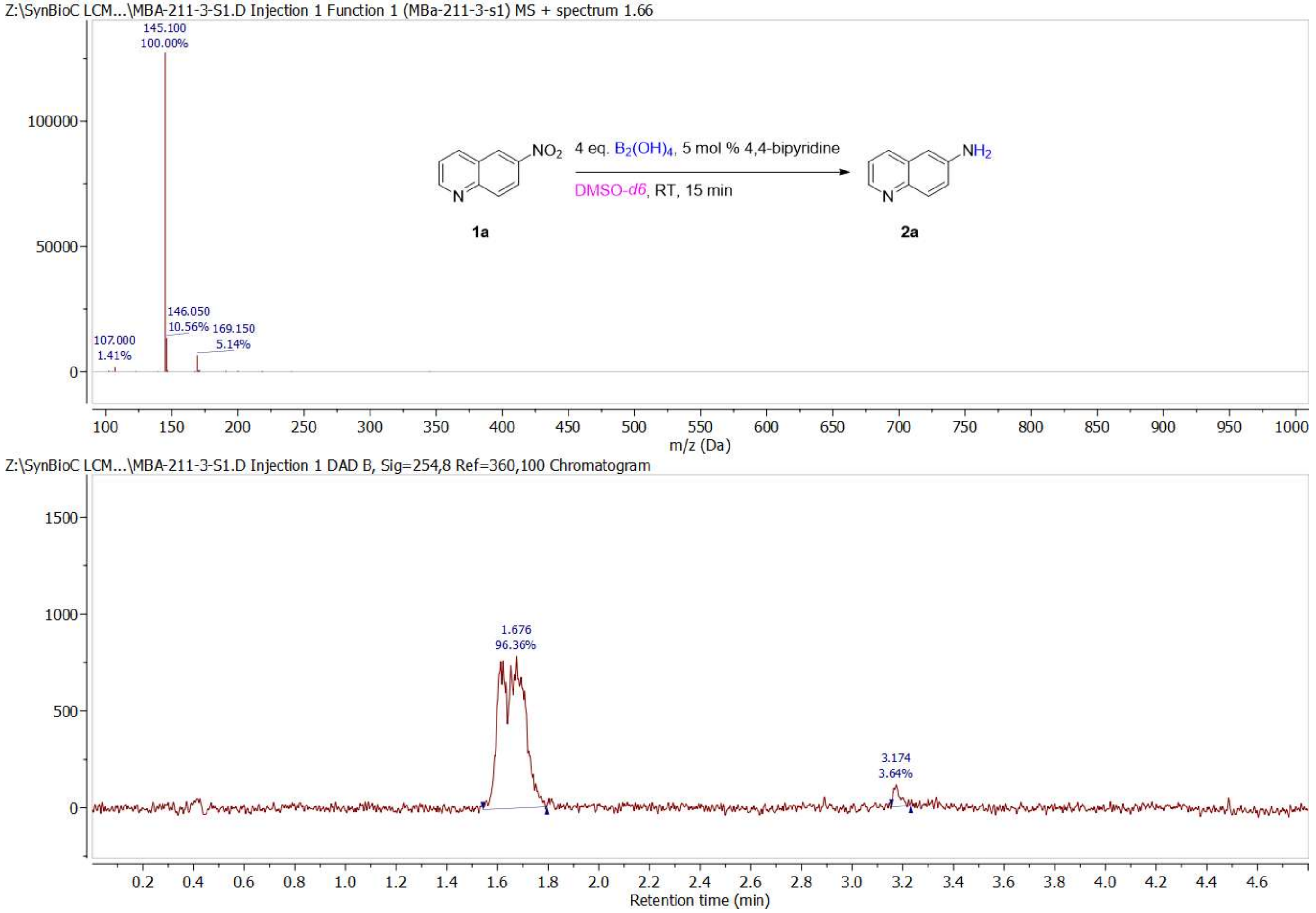

Figure S12 LC-MS analysis of crude reaction mixture. Deuterium labeling control experiments for the nitro reduction with DMSO-d6 as solvent.

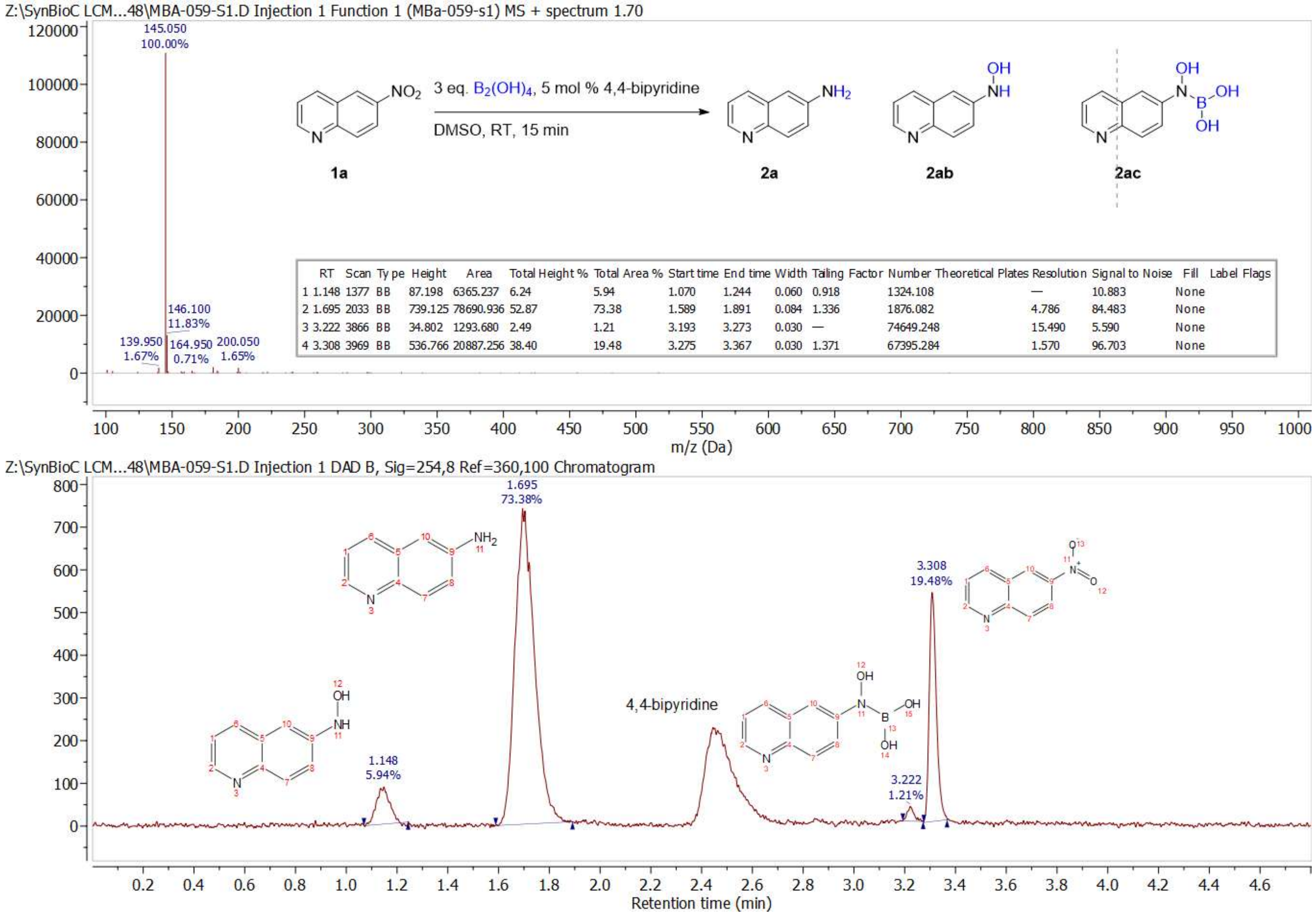

	RT	Scan	Type	Height	Area	Total Height %	Total Area %	Start time	End time	Width	Tailing Factor	Number Theoretical Plates	Resolution	Signal to Noise	Fill	Label	Flags
1	1.148	1377	BB	87.198	6365.237	6.24	5.94	1.070	1.244	0.060	0.918	1324.108	—	10.883	None		
2	1.695	2033	BB	739.125	78690.936	52.87	73.38	1.589	1.891	0.084	1.336	1876.082	4.786	84.483	None		
3	3.222	3866	BB	34.802	1293.680	2.49	1.21	3.193	3.273	0.030	—	74649.248	15.490	5.590	None		
4	3.308	3969	BB	536.766	20887.256	38.40	19.48	3.275	3.367	0.030	1.371	67395.284	1.570	96.703	None		

Figure S13 LC-MS analysis of crude reaction mixture. Control experiment with use of 3 eq. $B_2(OH)_4$ and DMSO as solvent.

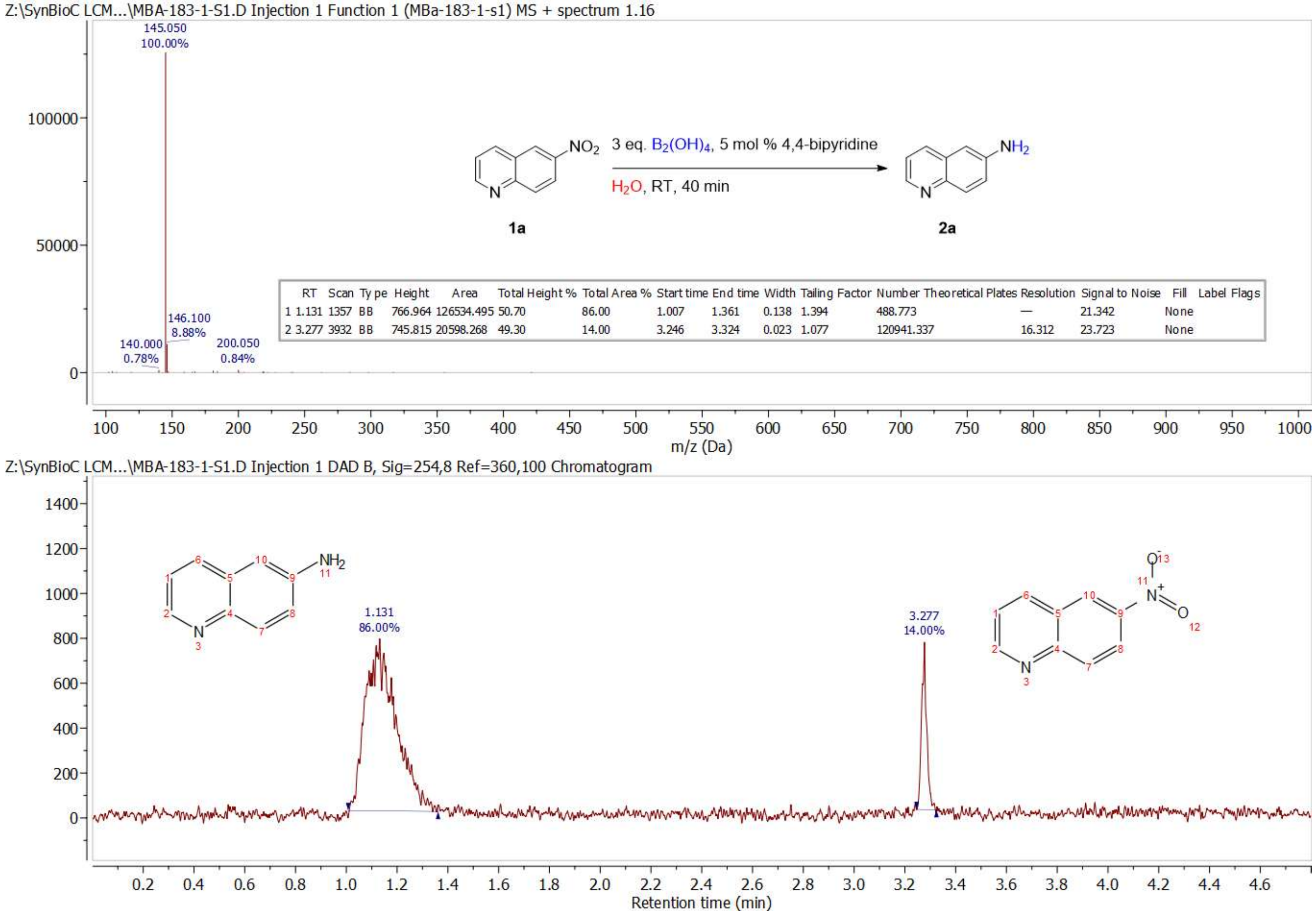

	RT	Scan	Type	Height	Area	Total Height %	Total Area %	Start time	End time	Width	Tailing Factor	Number Theoretical Plates	Resolution	Signal to Noise	Fill	Label	Flags
1	1.131	1357	BB	766.964	126534.495	50.70	86.00	1.007	1.361	0.138	1.394	488.773	—	21.342	None		
2	3.277	3932	BB	745.815	20598.268	49.30	14.00	3.246	3.324	0.023	1.077	120941.337	16.312	23.723	None		

Figure S14 LC-MS analysis of crude reaction mixture. Control experiment with use of 3 eq. $B_2(OH)_4$ and H_2O as solvent.

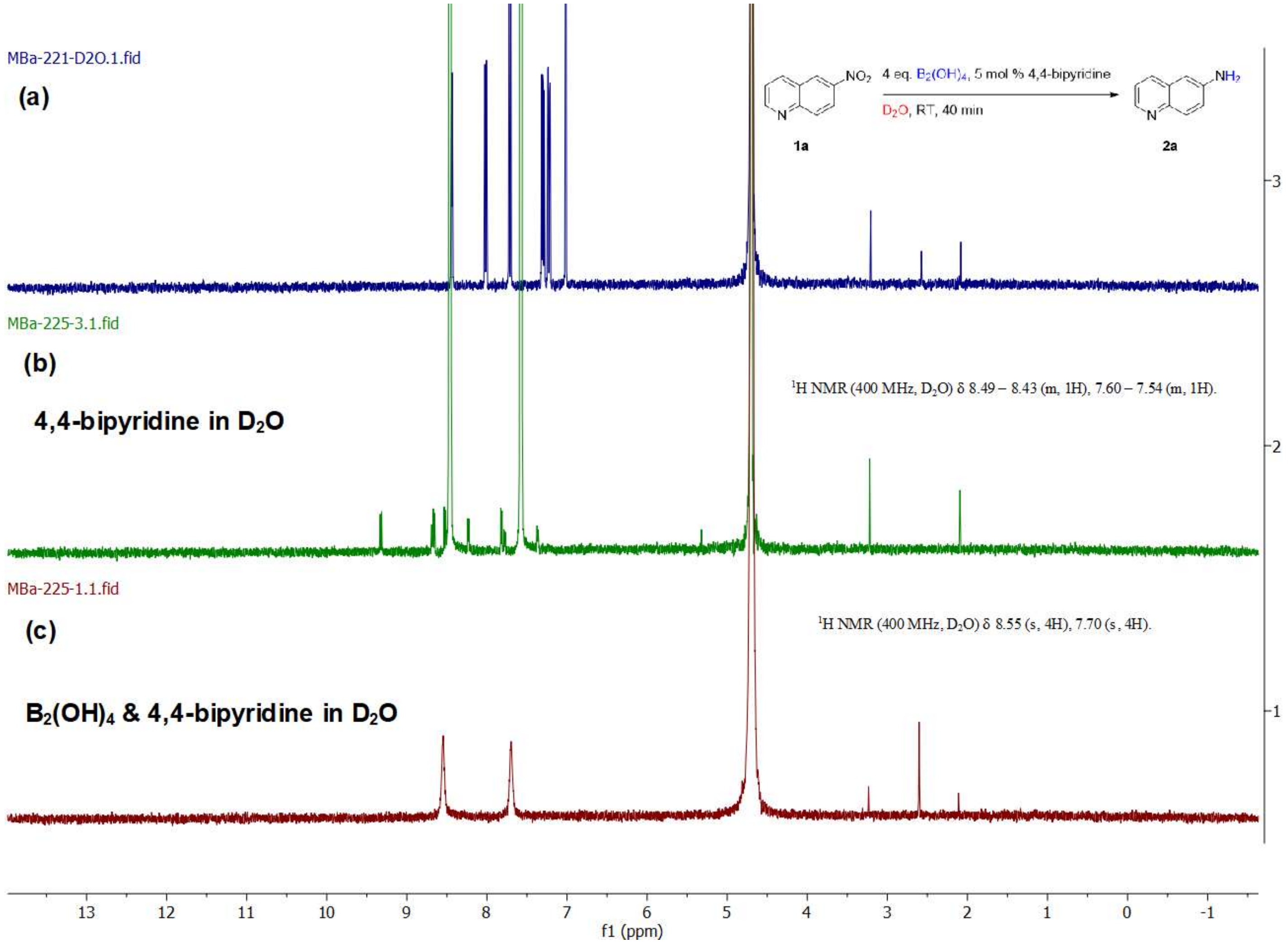

Figure S15 ^{1}H-NMR control experiments.

4. Spectral copies of ^{1}H, ^{13}C NMR of the products.

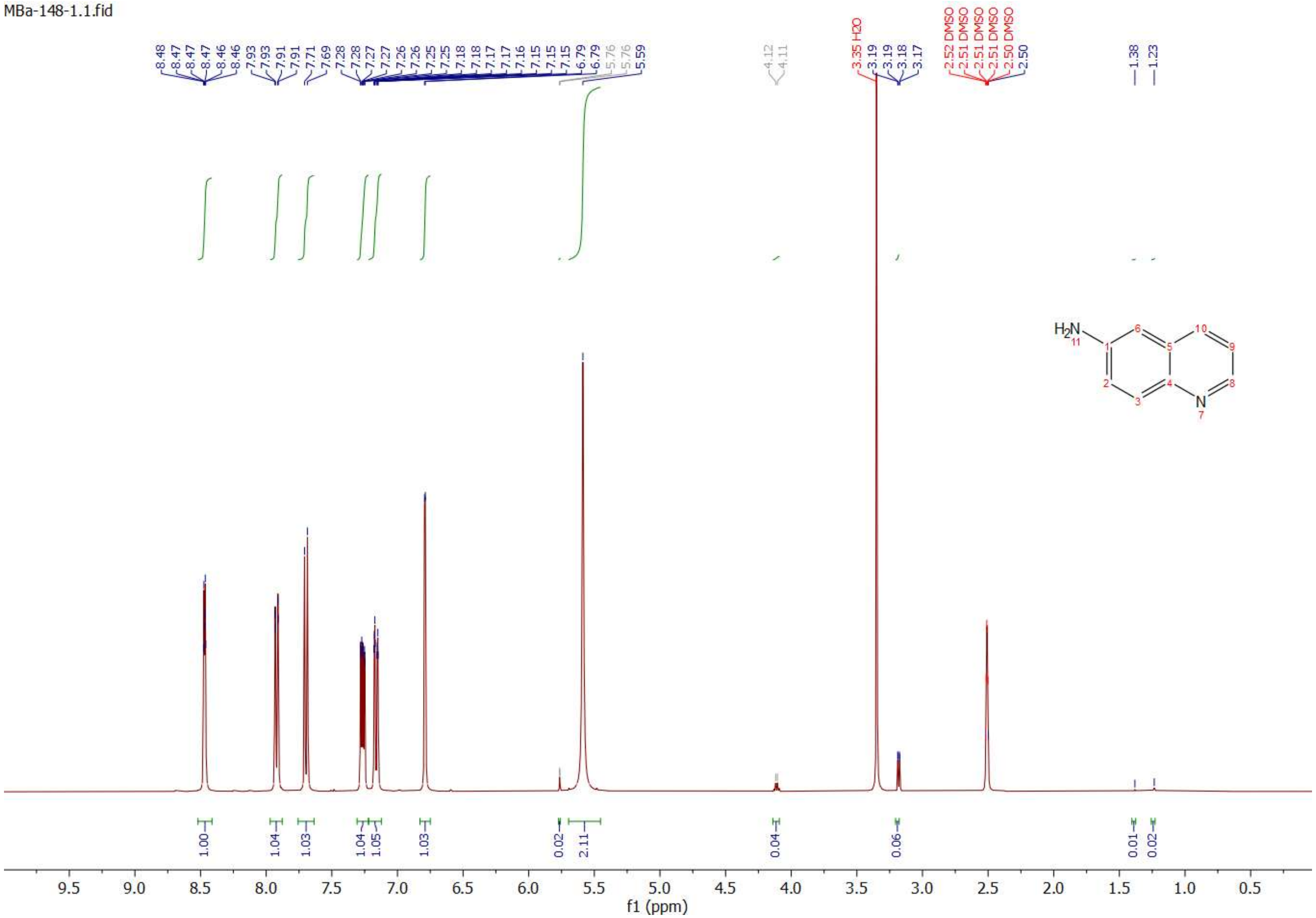

Figure S16 ^{1}H NMR **2a** in DMSO-*d6*.

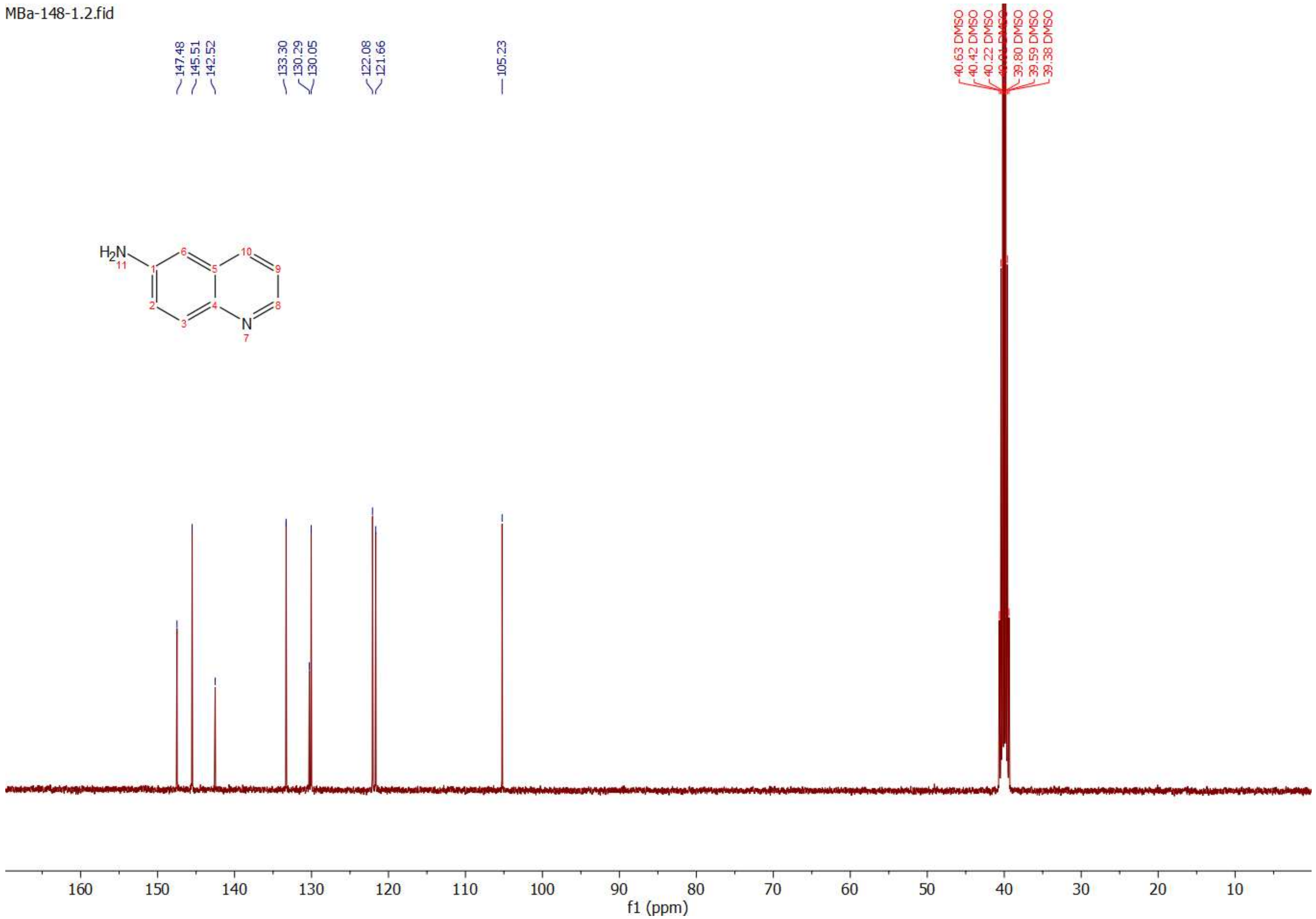

Figure S17 ^{13}C NMR **2a** in DMSO-*d6*.

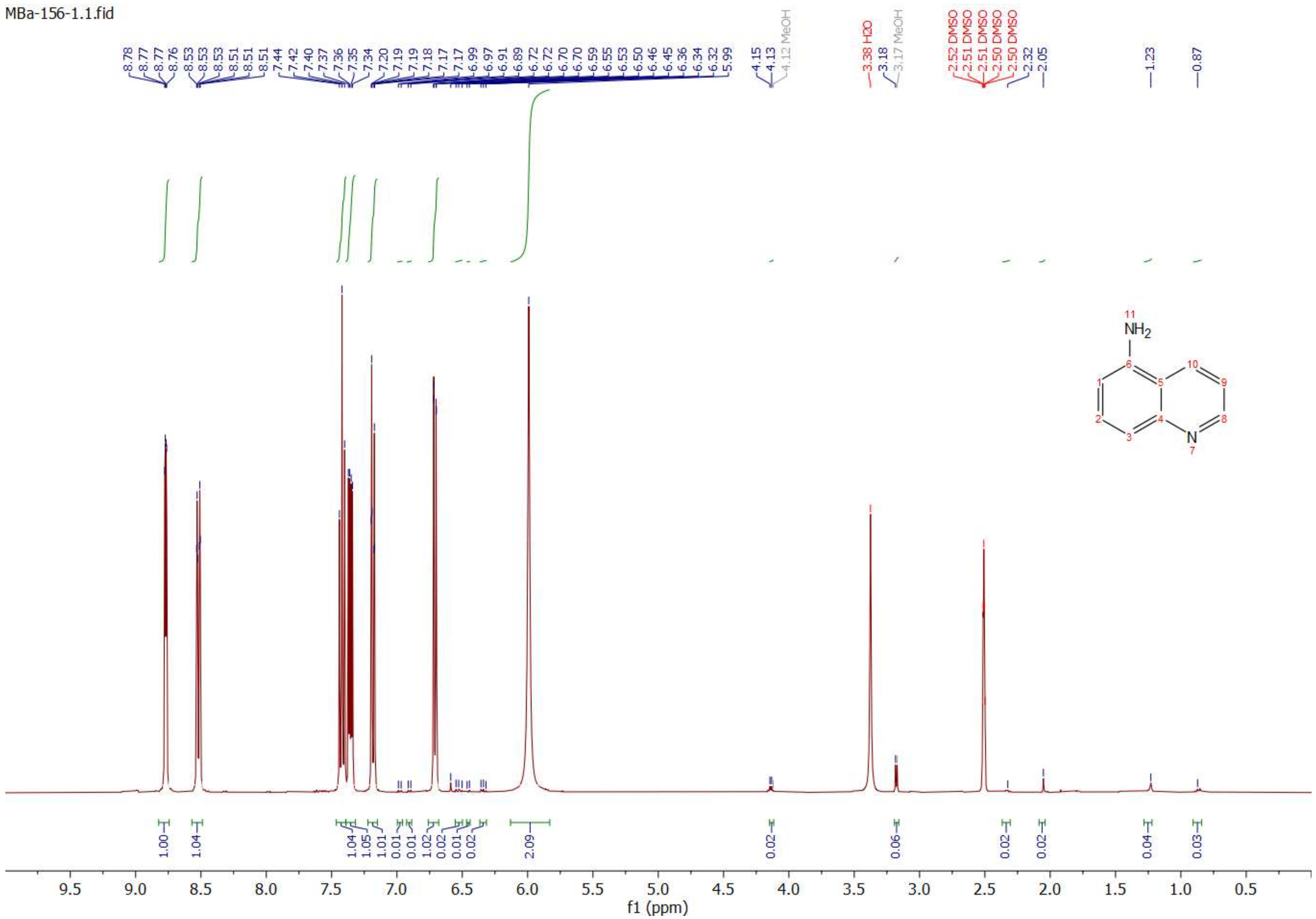

Figure S18 ^{1}H NMR **2b** in DMSO-*d6*.

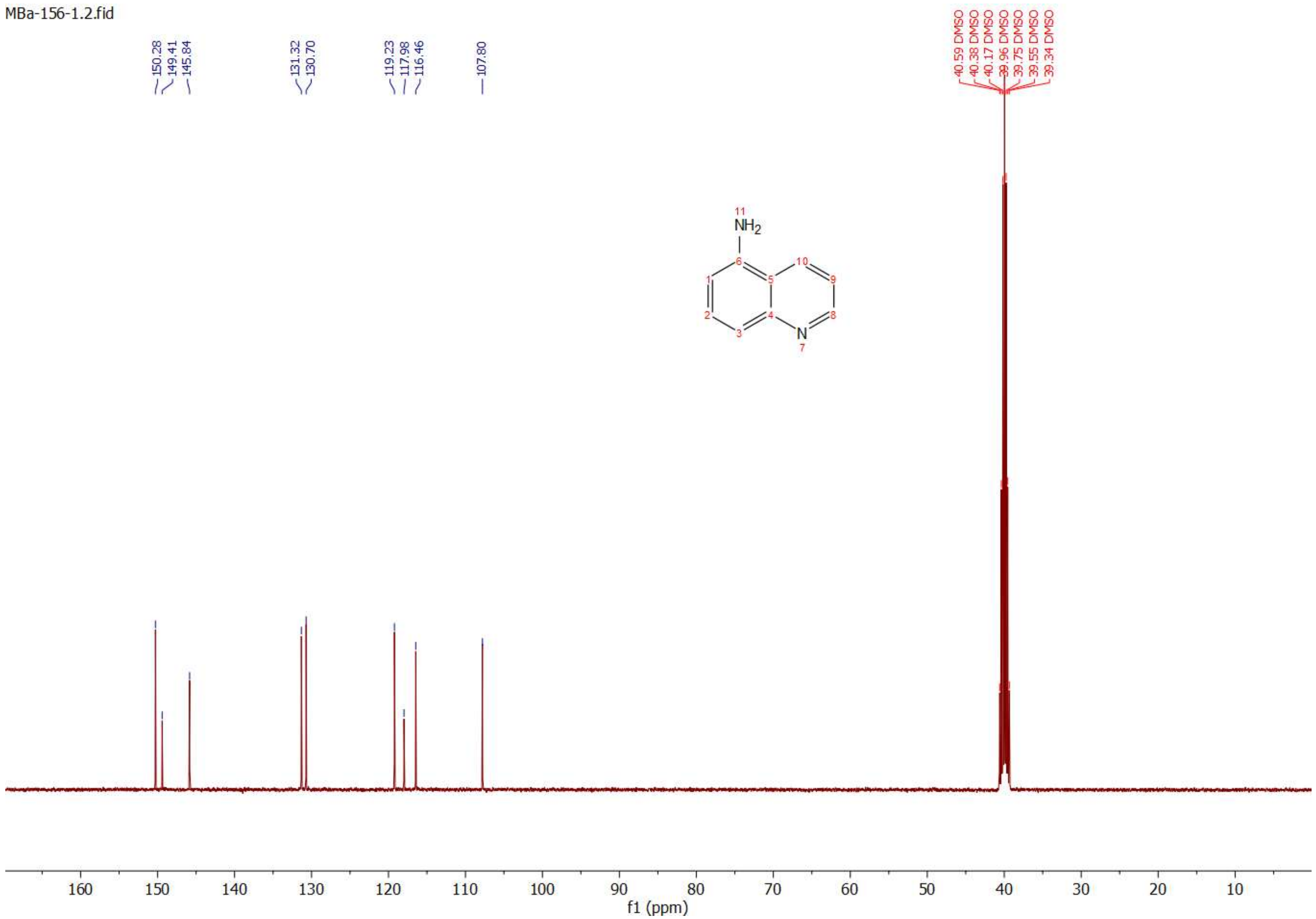

Figure S19 ^{13}C NMR **2b** in DMSO-*d6*.

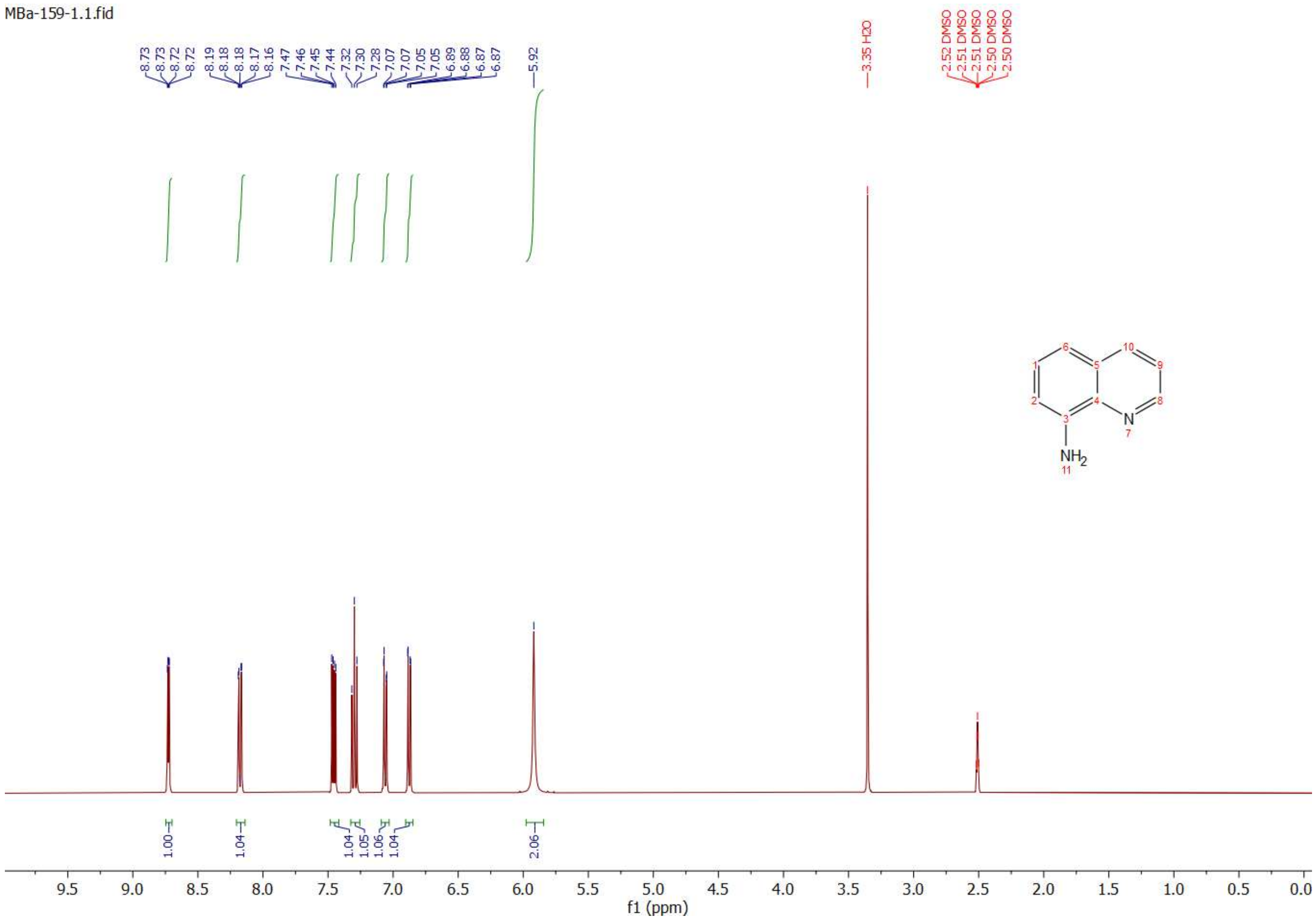

Figure S20 ^{1}H NMR **2c** in DMSO-*d6*.

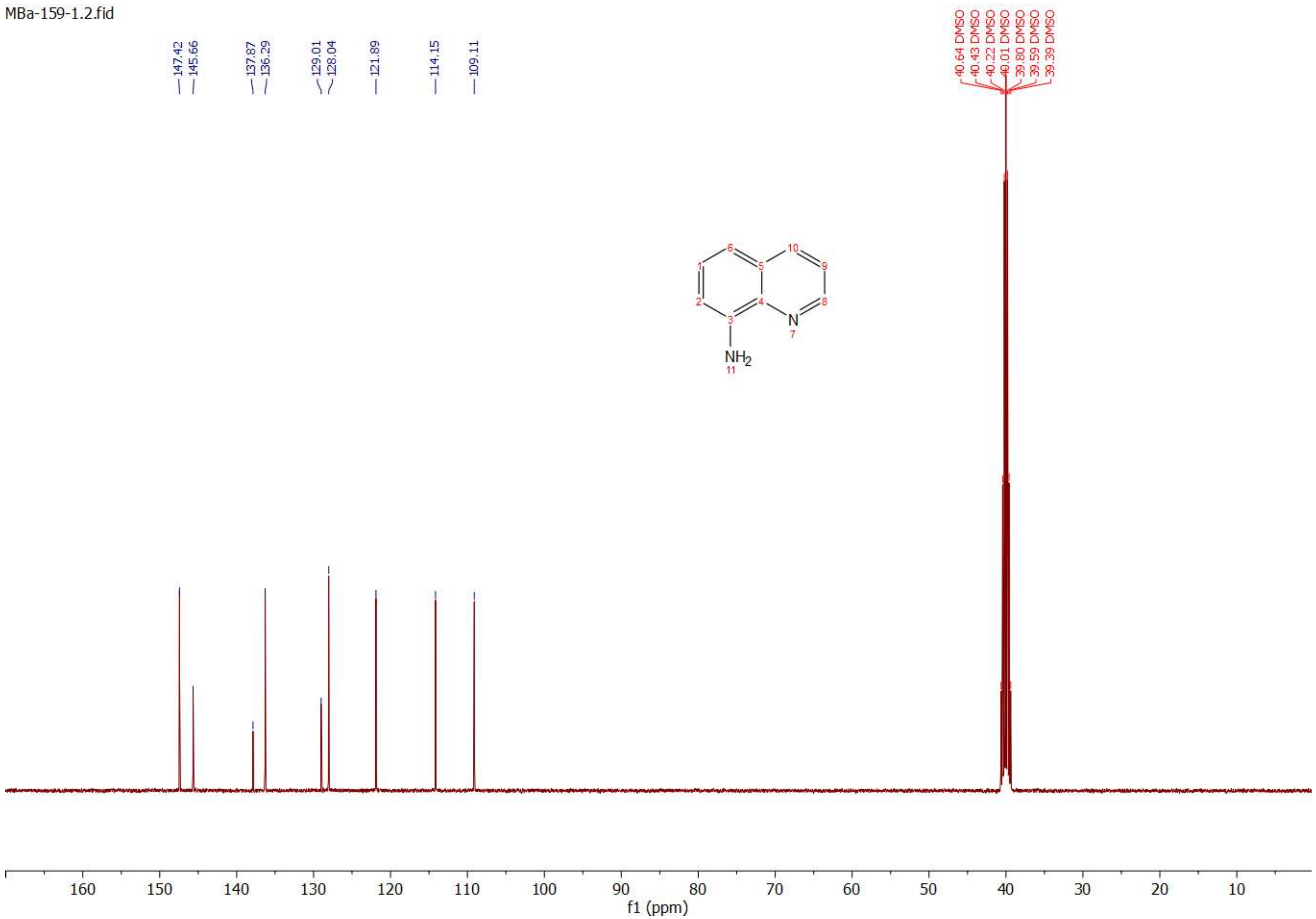

Figure S21 ^{13}C NMR **2c** in DMSO-*d6*.

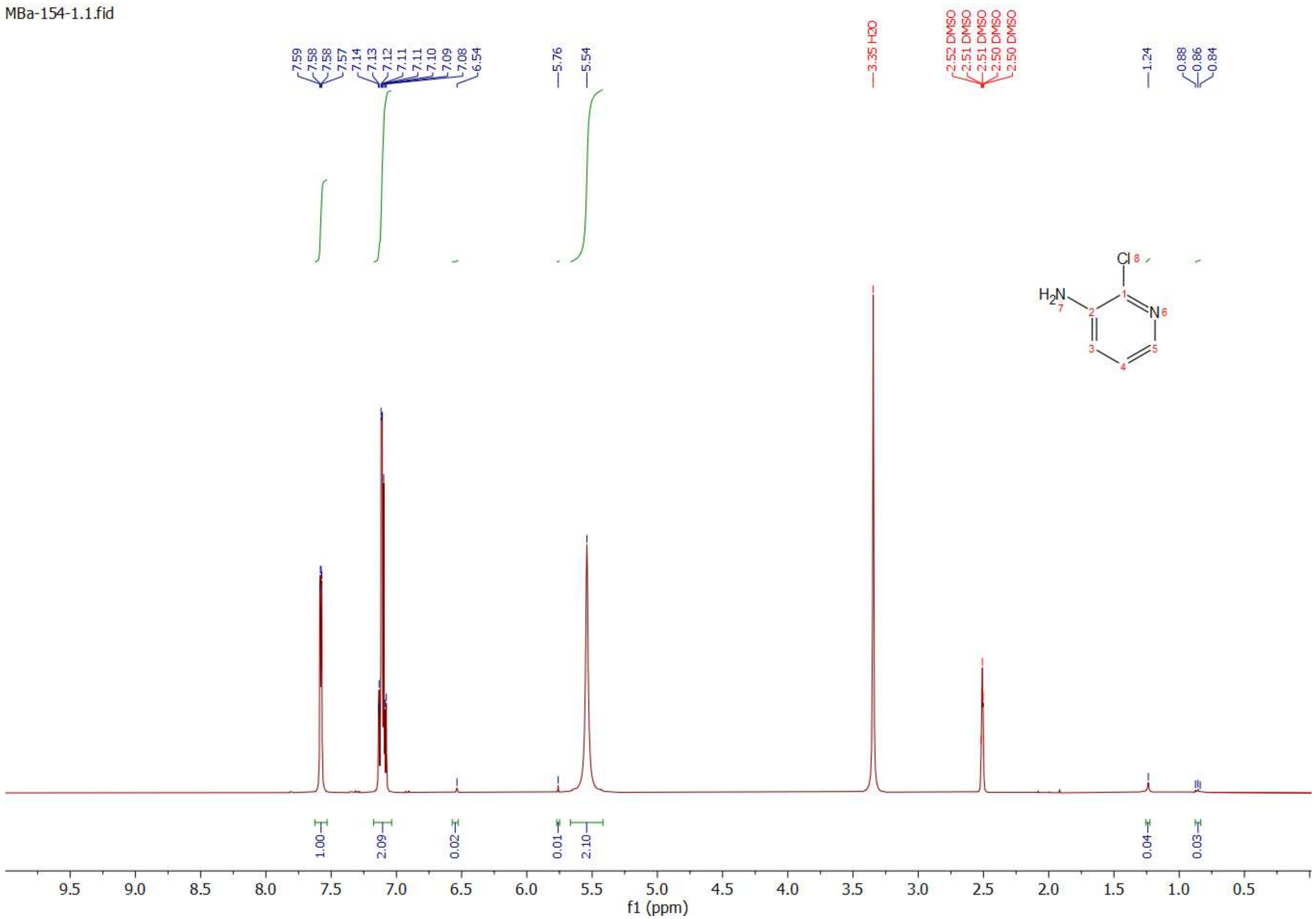

Figure S22 ^{1}H NMR **2d** in DMSO-*d6*.

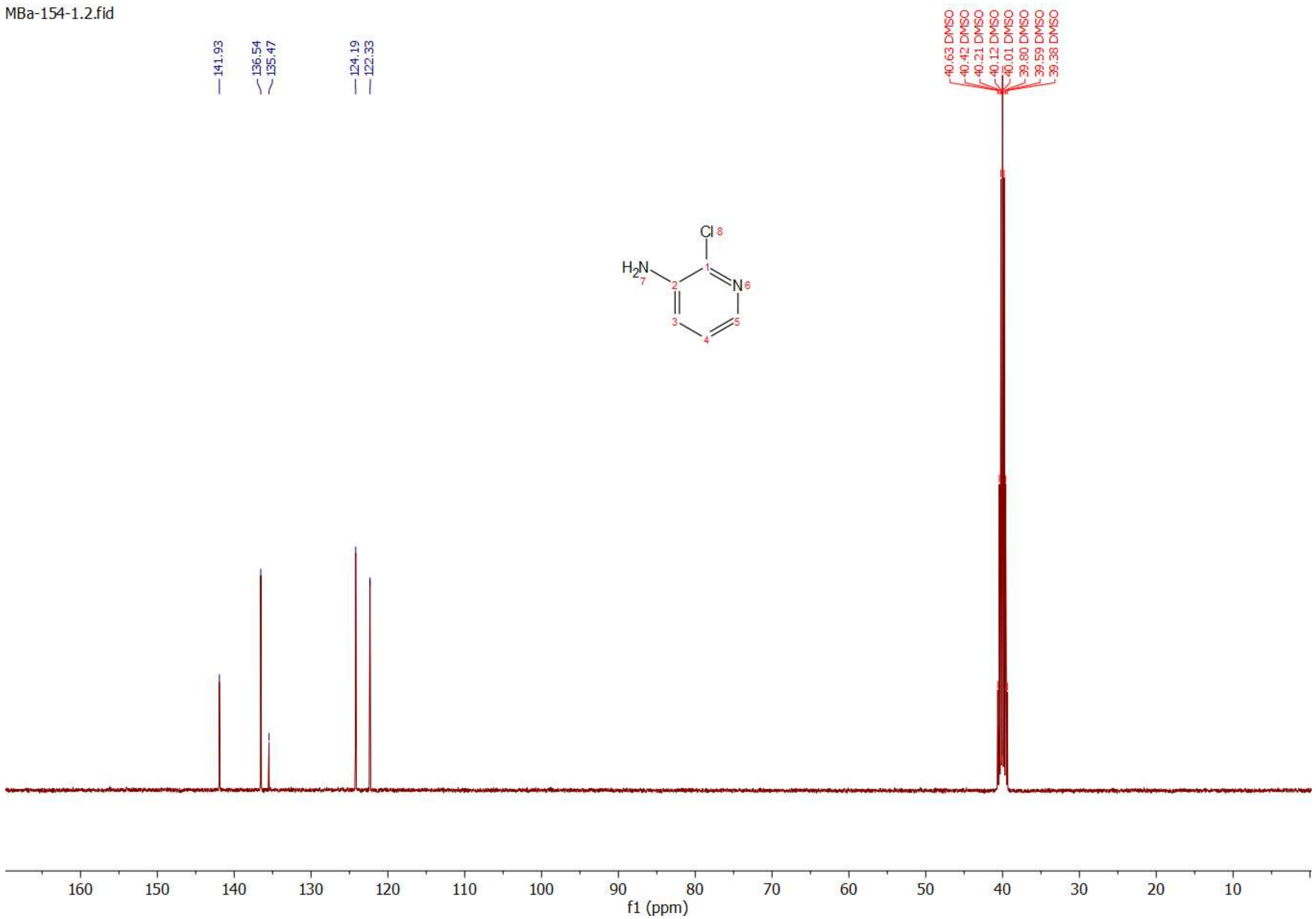

Figure S23 ^{13}C NMR **2d** in DMSO-*d6*.

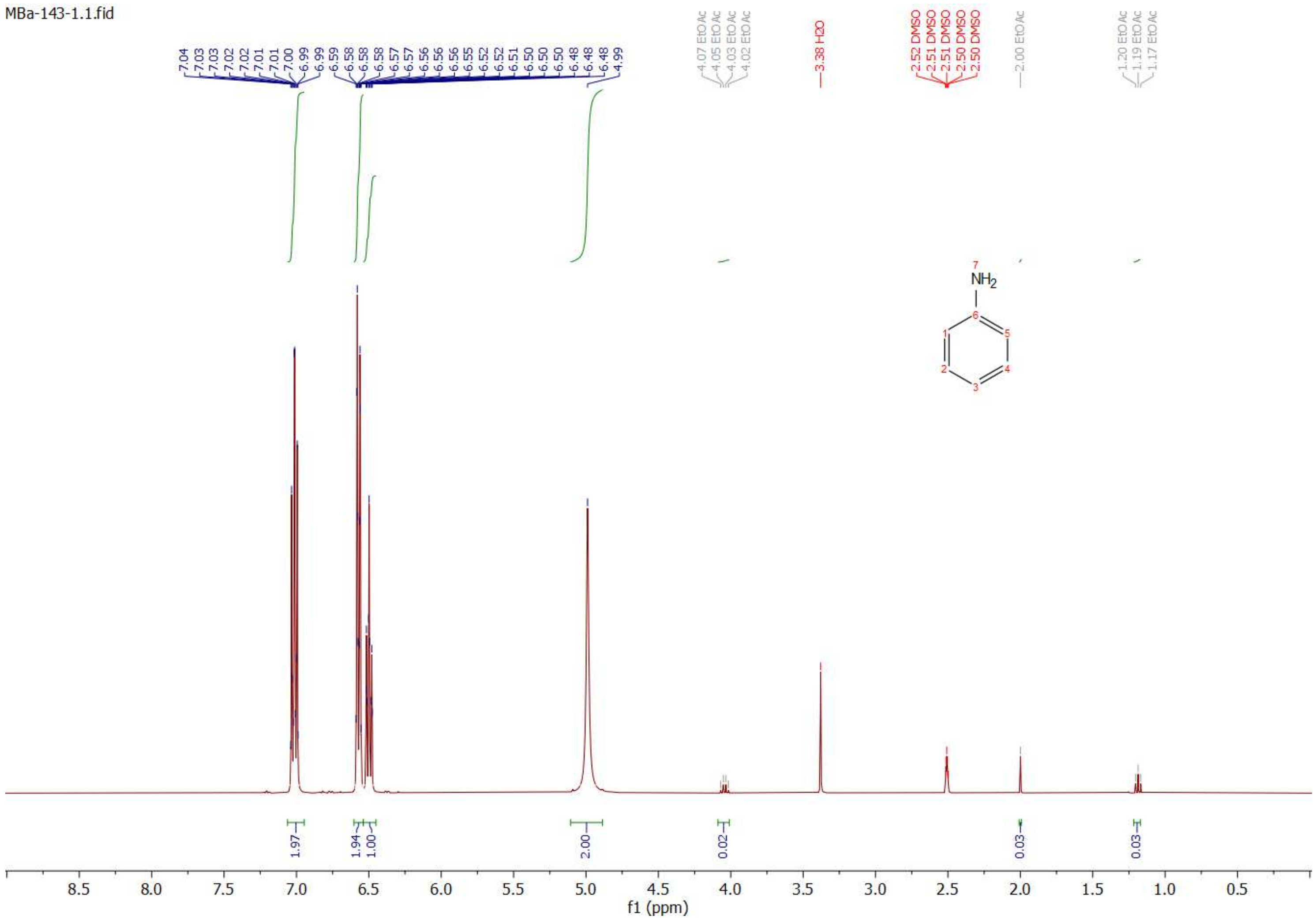

Figure S24 ^{1}H NMR **2e** in DMSO-*d6*.

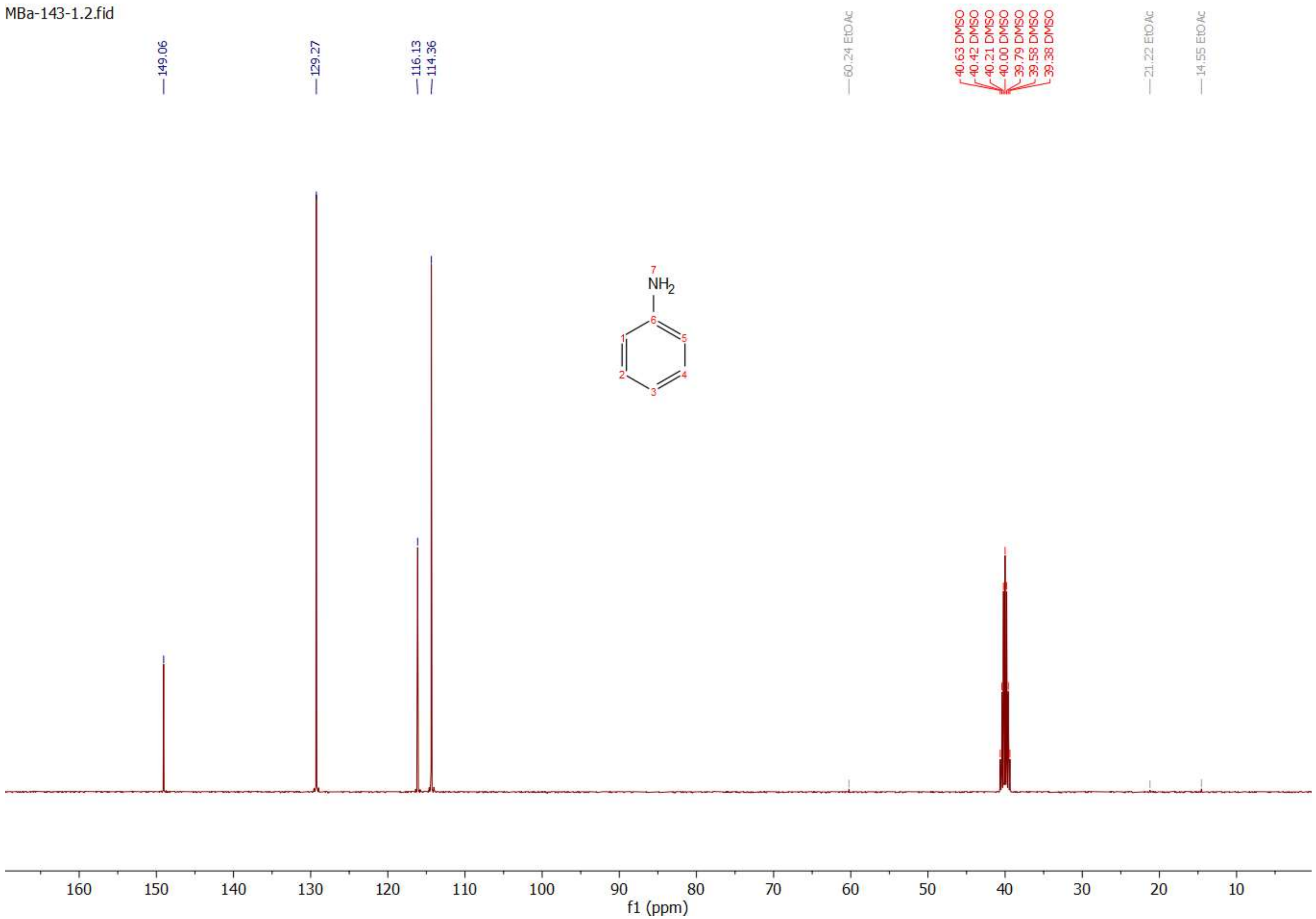

Figure S25 ^{13}C NMR **2e** in DMSO-*d6*.

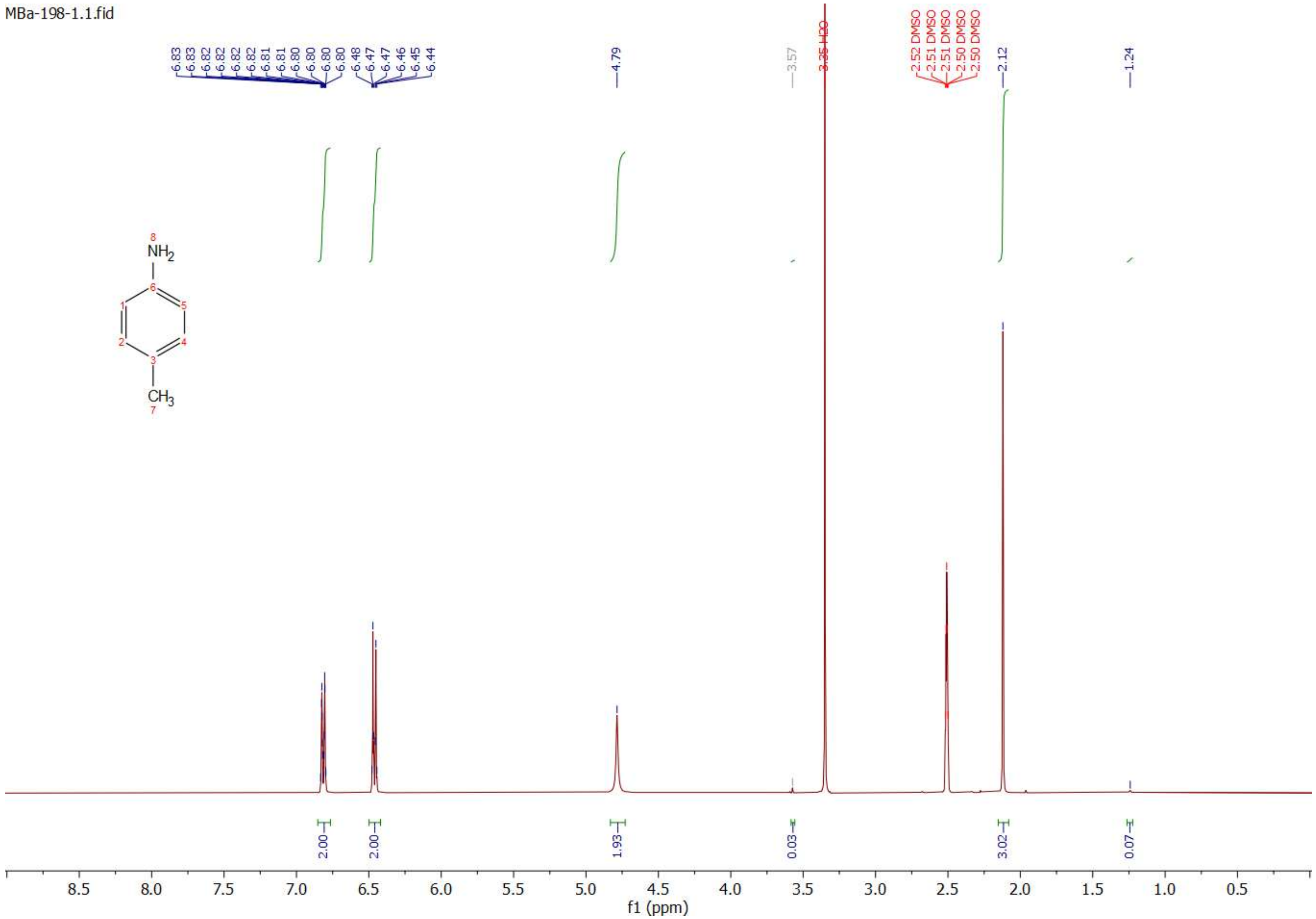

Figure S26 ^{1}H NMR **2f** in DMSO-*d6*.

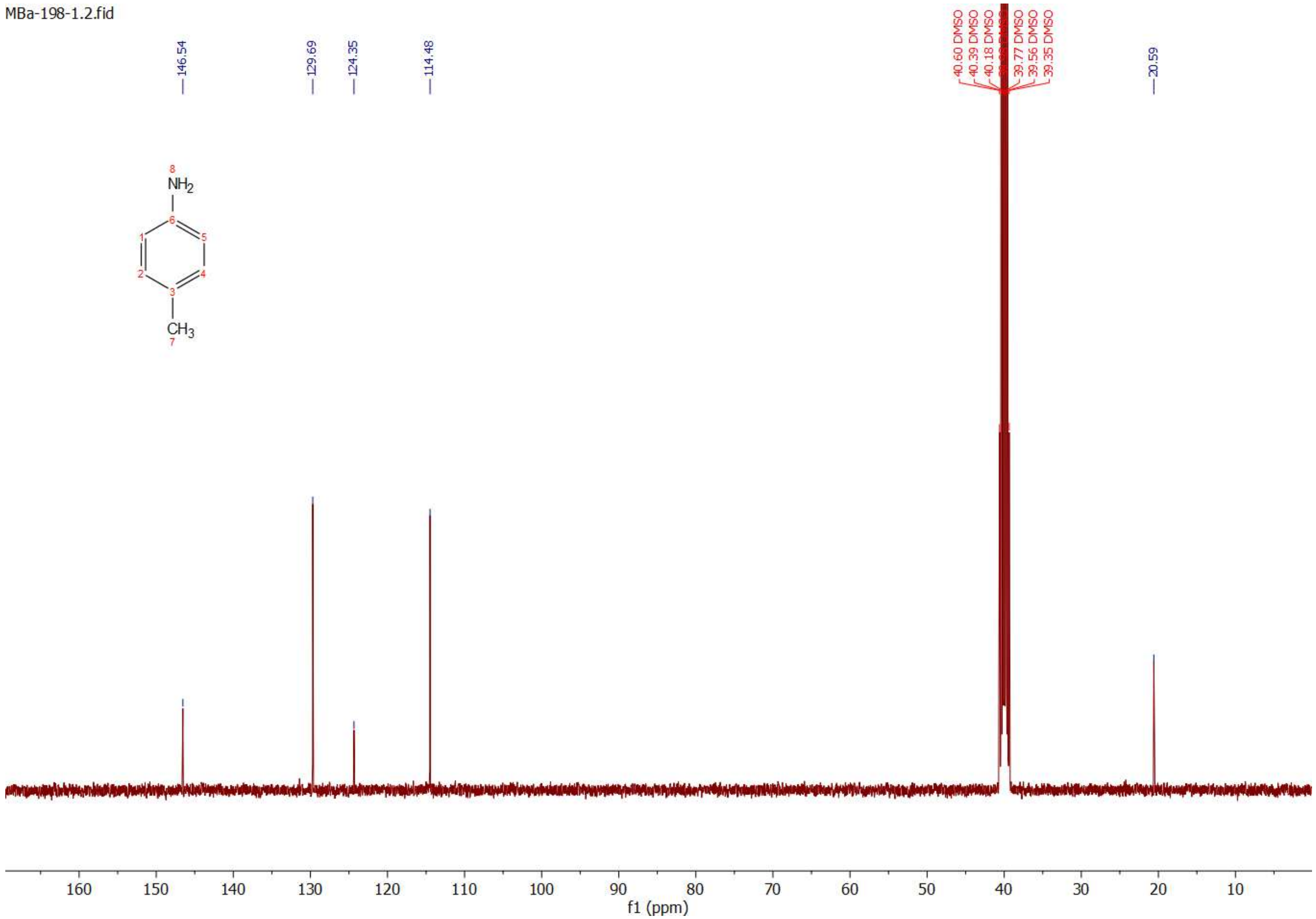

Figure S27 ^{13}C NMR **2f** in DMSO-d6.

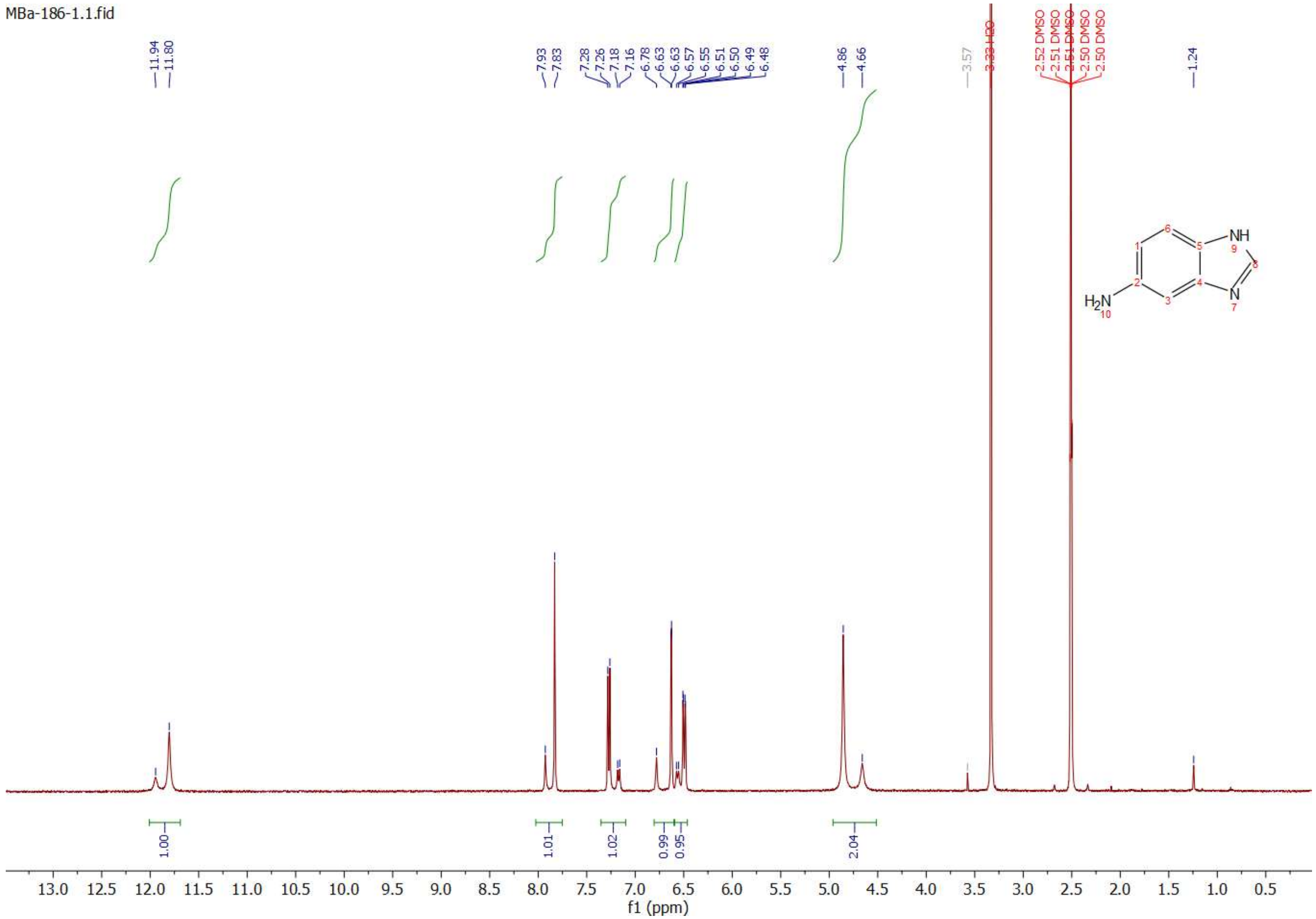

Figure S28 ^{1}H NMR **2g** in DMSO-*d6* (sample low C).

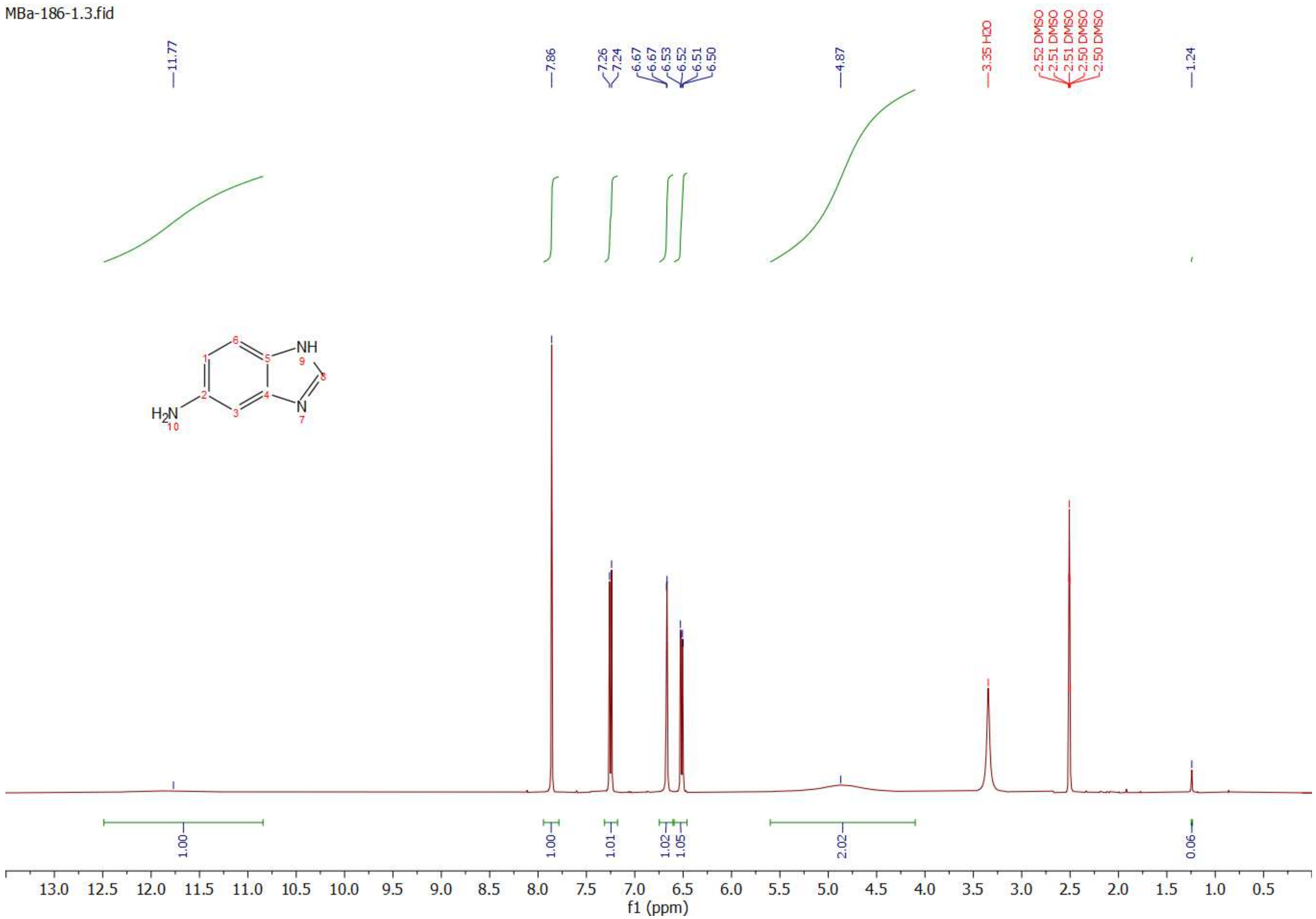

Figure S29 ^{1}H NMR **2g** in DMSO-*d6* (sample high C).

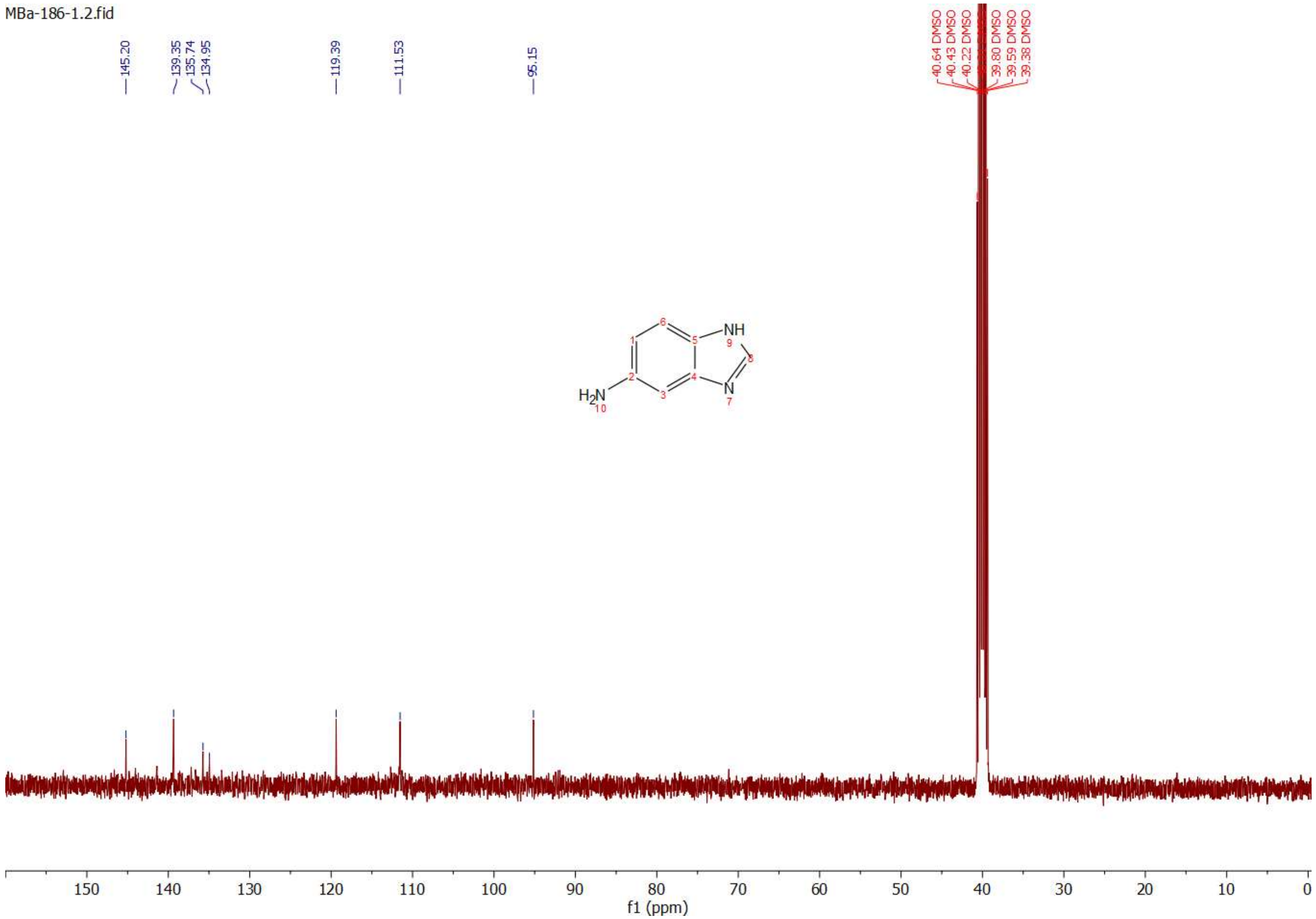

Figure S30 ^{13}C NMR **2g** in DMSO-*d6* (sample low C).

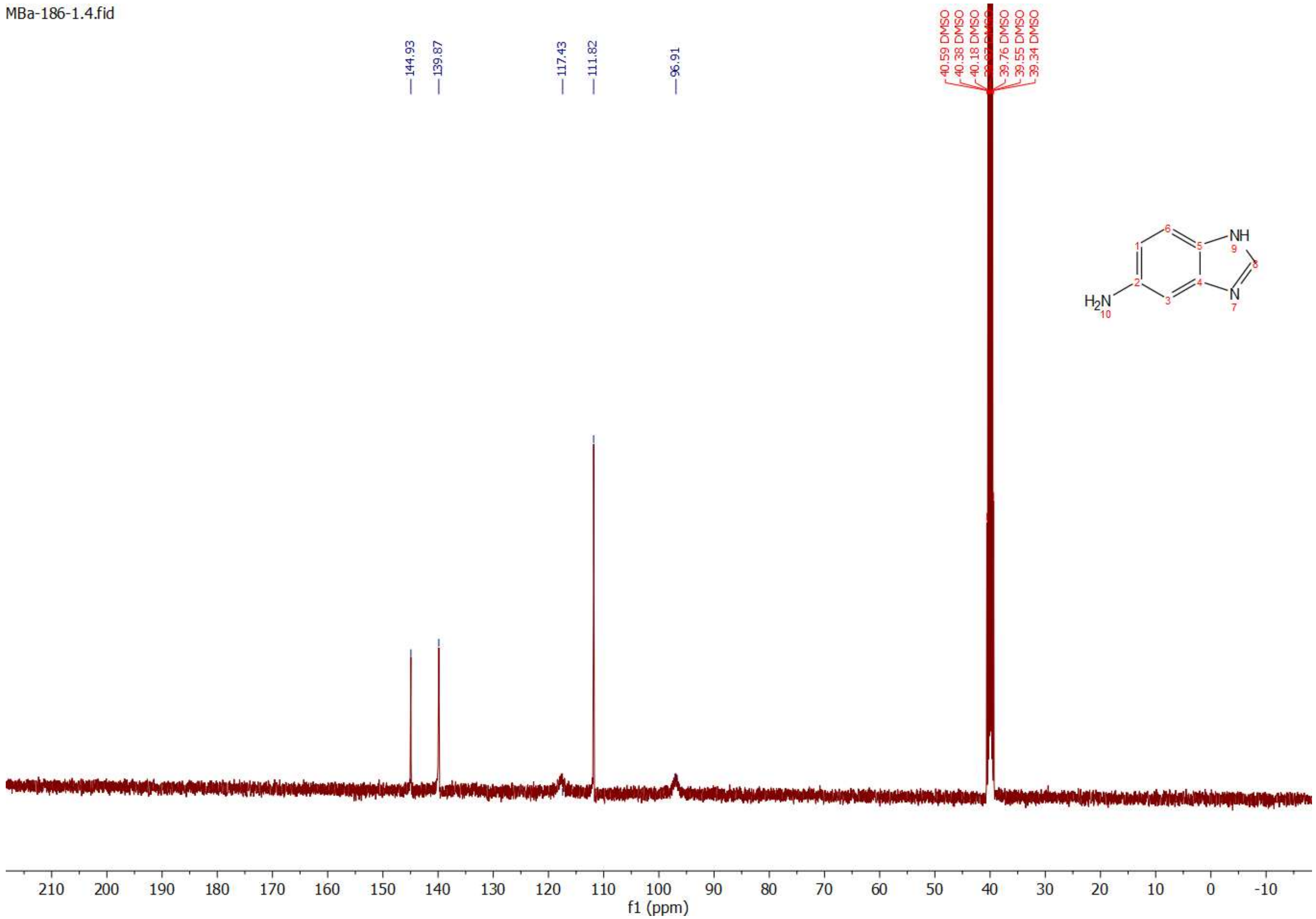

Figure S31 ^{13}C NMR **2g** in DMSO-*d6* (sample high C).

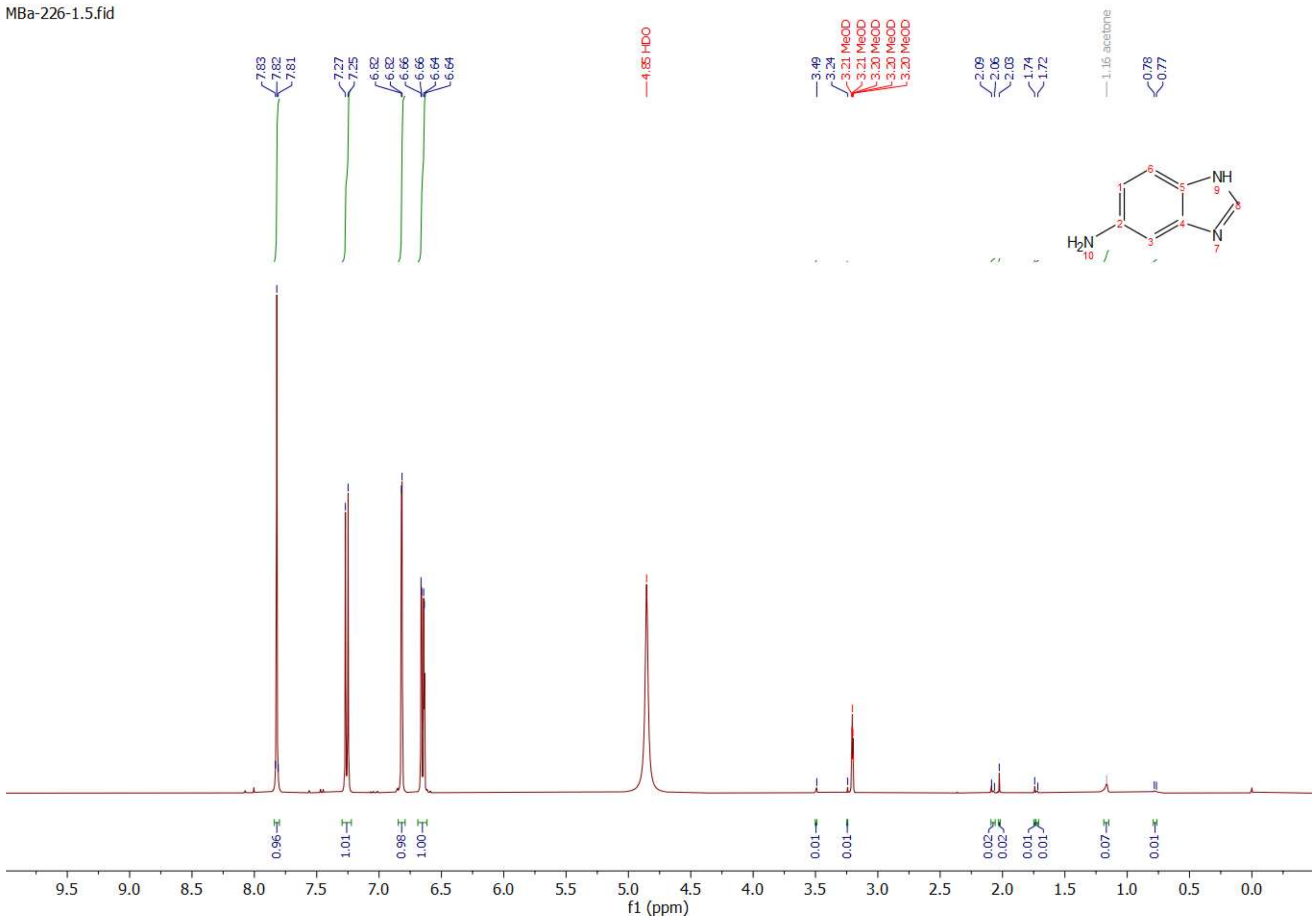

Figure S32 ¹H NMR **2g** in MeOH-*d4*.

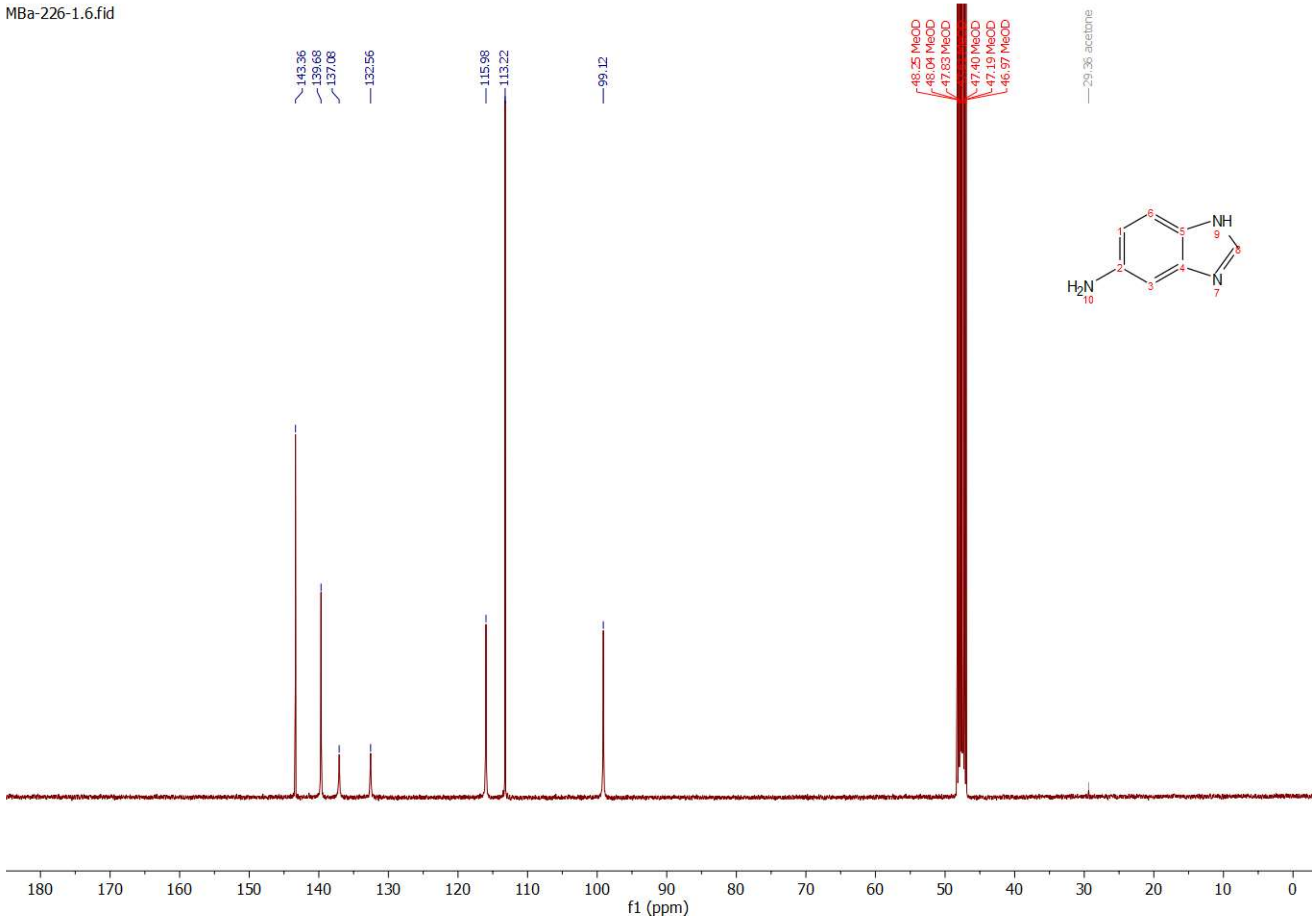

Figure S33 ^{13}C NMR **2g** in MeOH-*d4*.

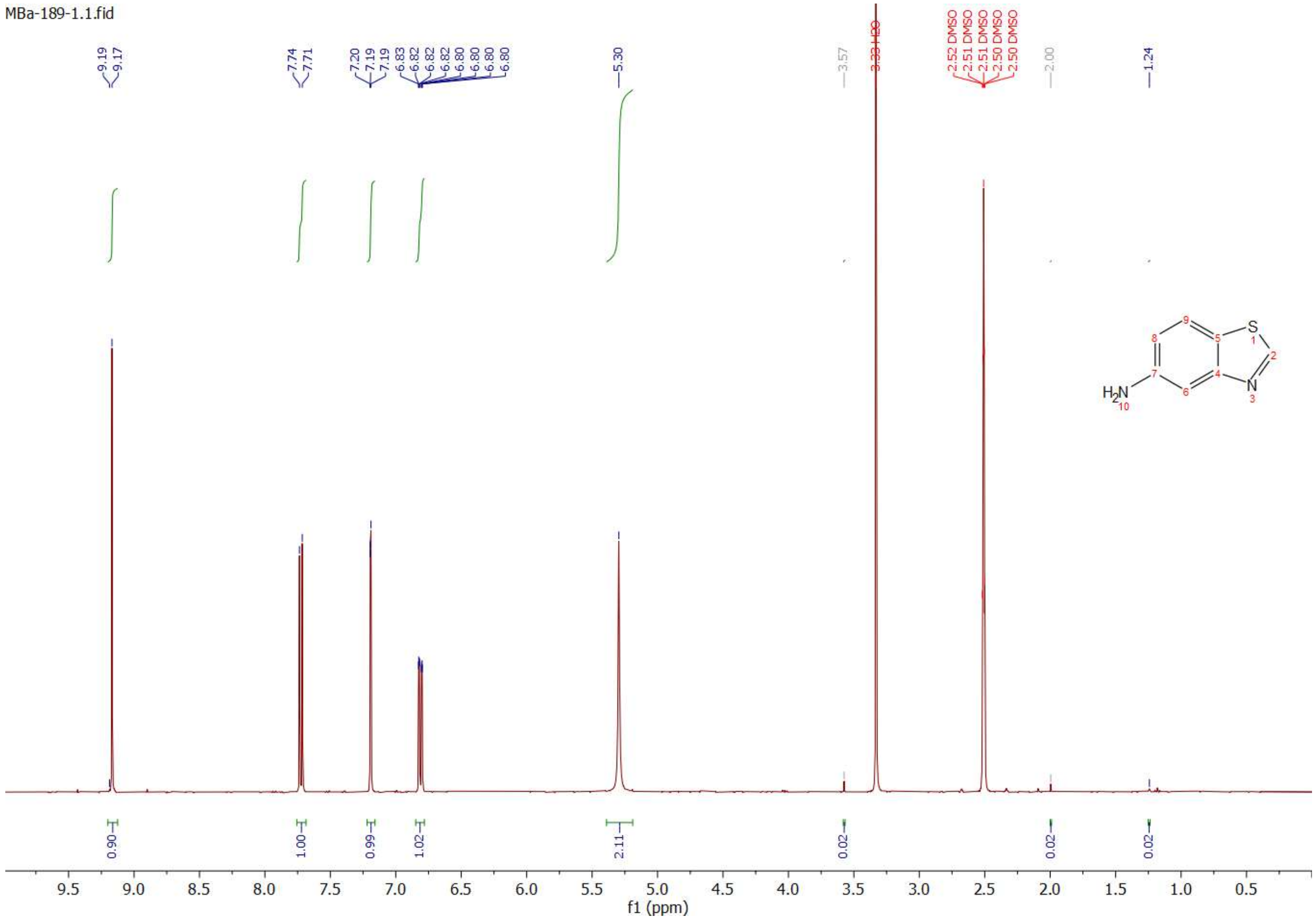

Figure S34 ¹H NMR **2h** in DMSO-*d6*.

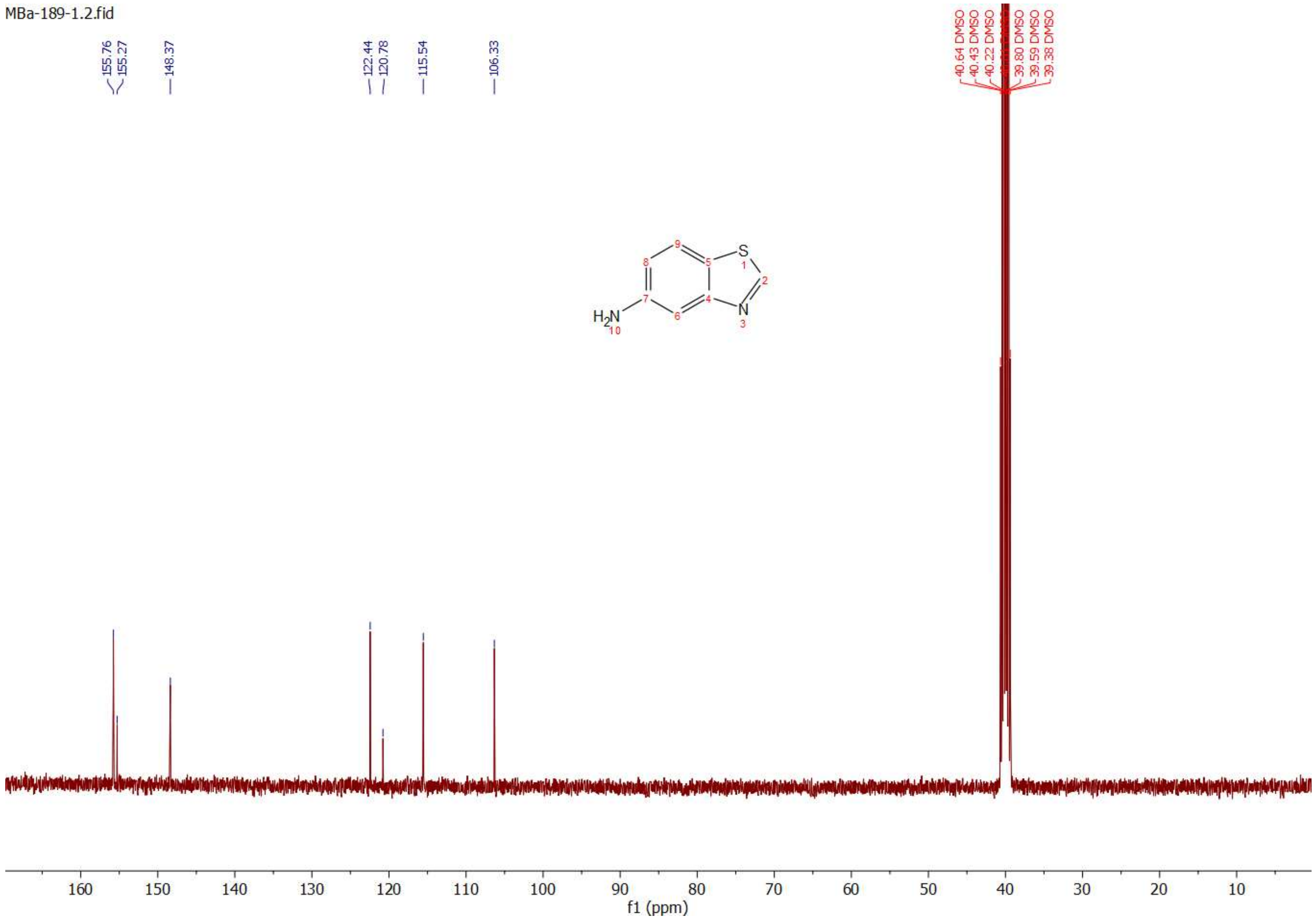

Figure S35 ^{13}C NMR **2h** in DMSO-*d6*.

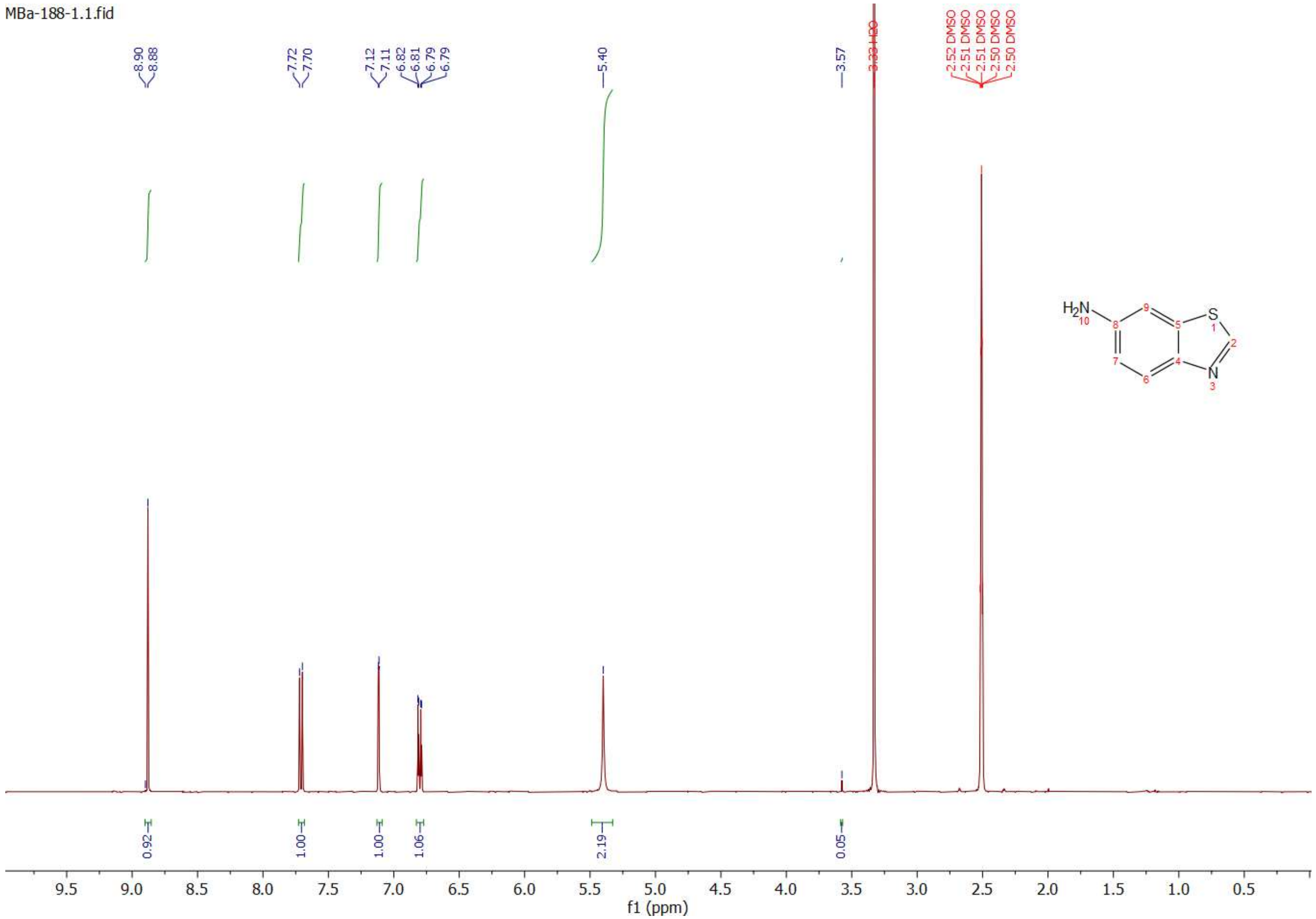

Figure S36 ^{1}H NMR **2i** in DMSO-*d6*.

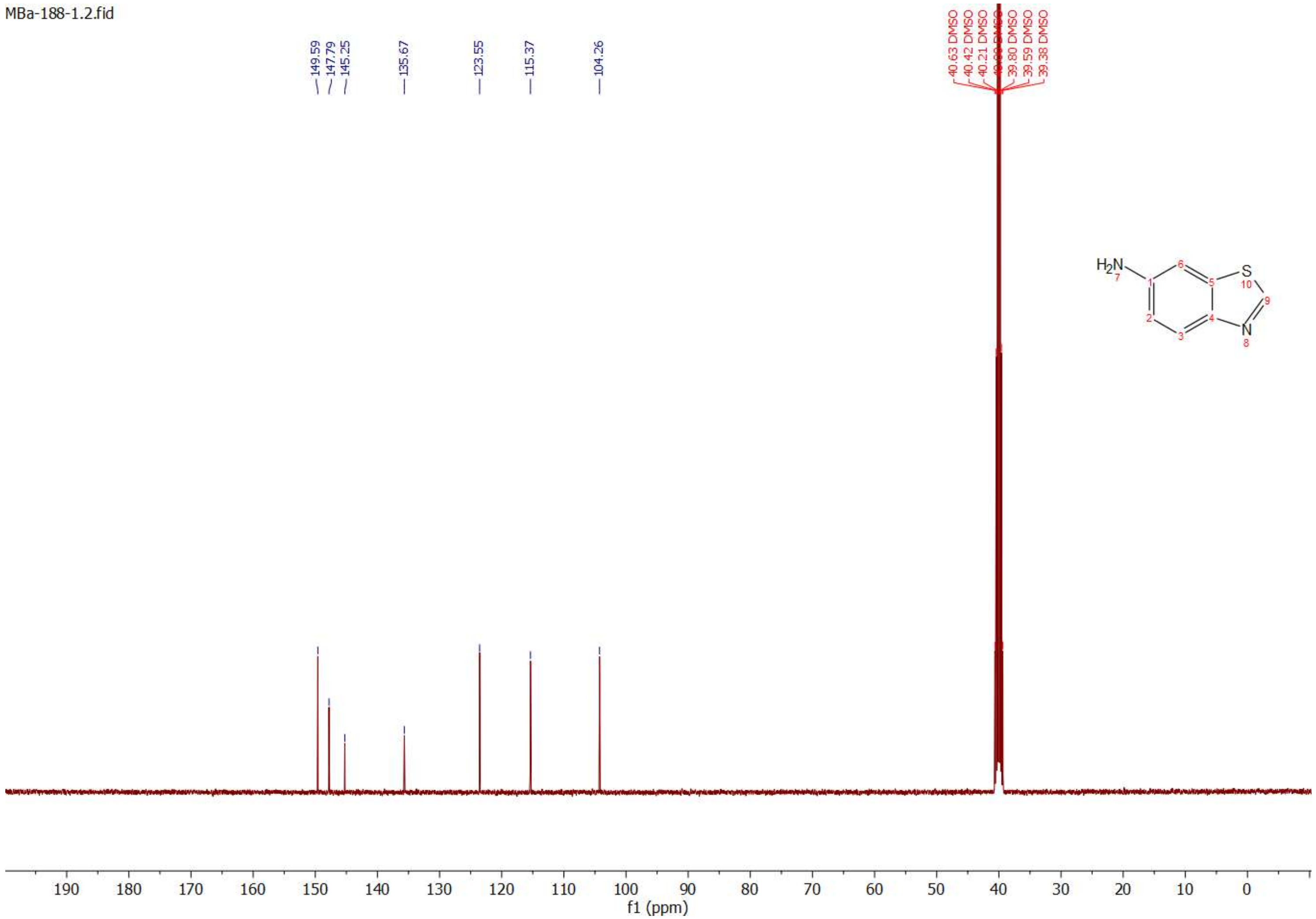

Figure S37 ^{13}C NMR **2i** in DMSO-*d6*.

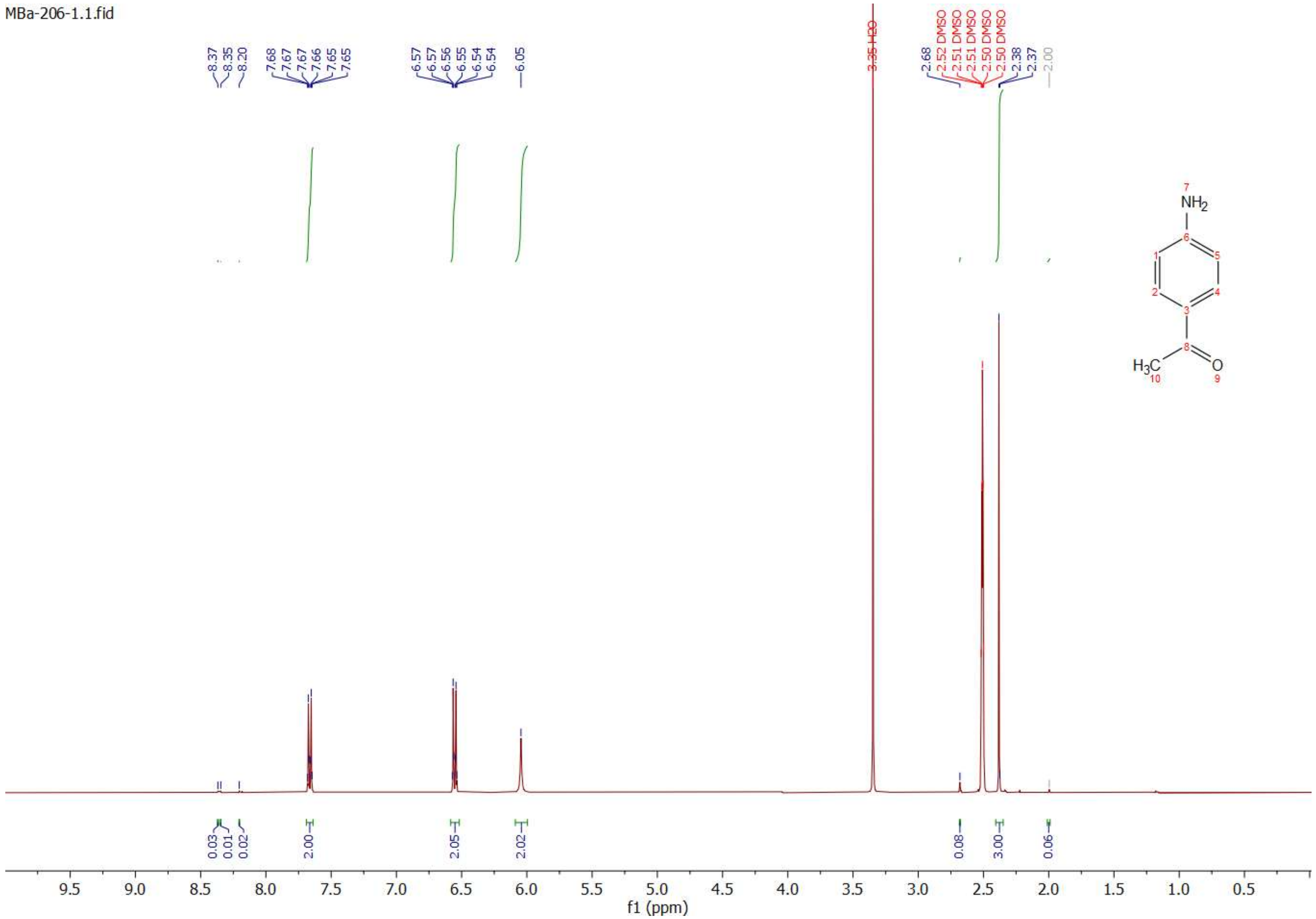

Figure S38 ¹H NMR **2j** in DMSO-*d6*.

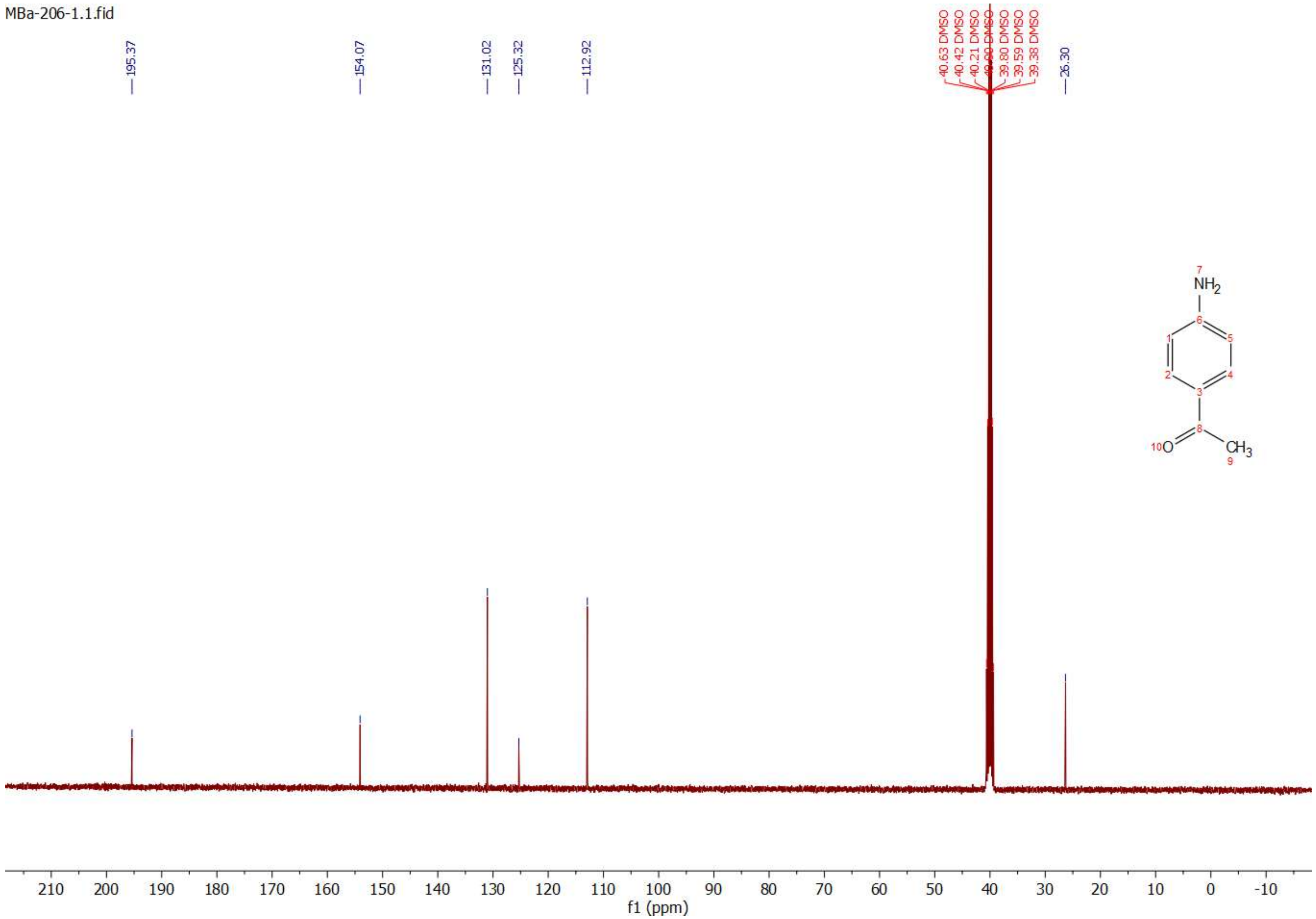

Figure S39 ^{13}C NMR **2j** in DMSO-*d6*.

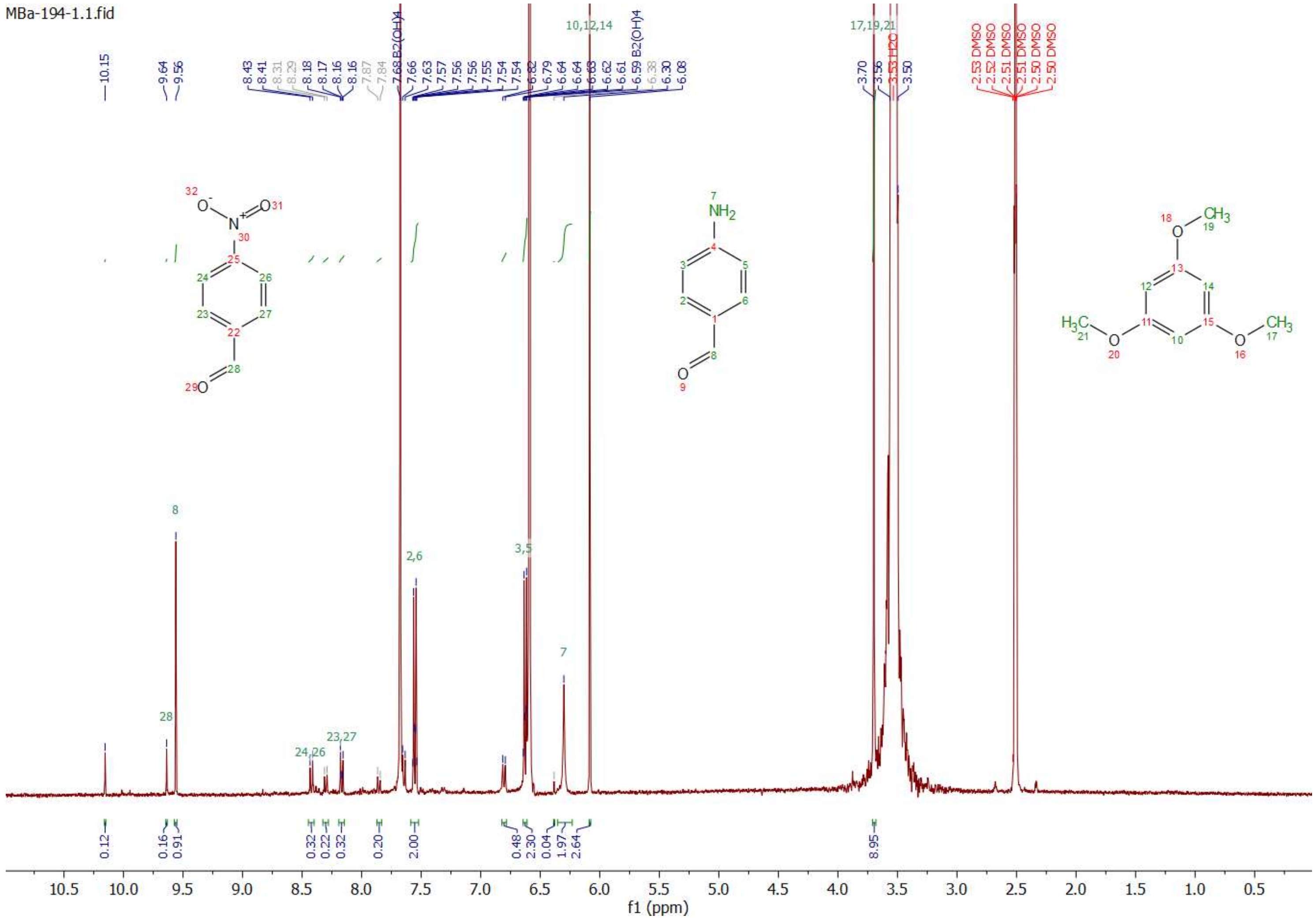

Figure S40 ^{1}H NMR **2ka** in DMSO-*d6*.

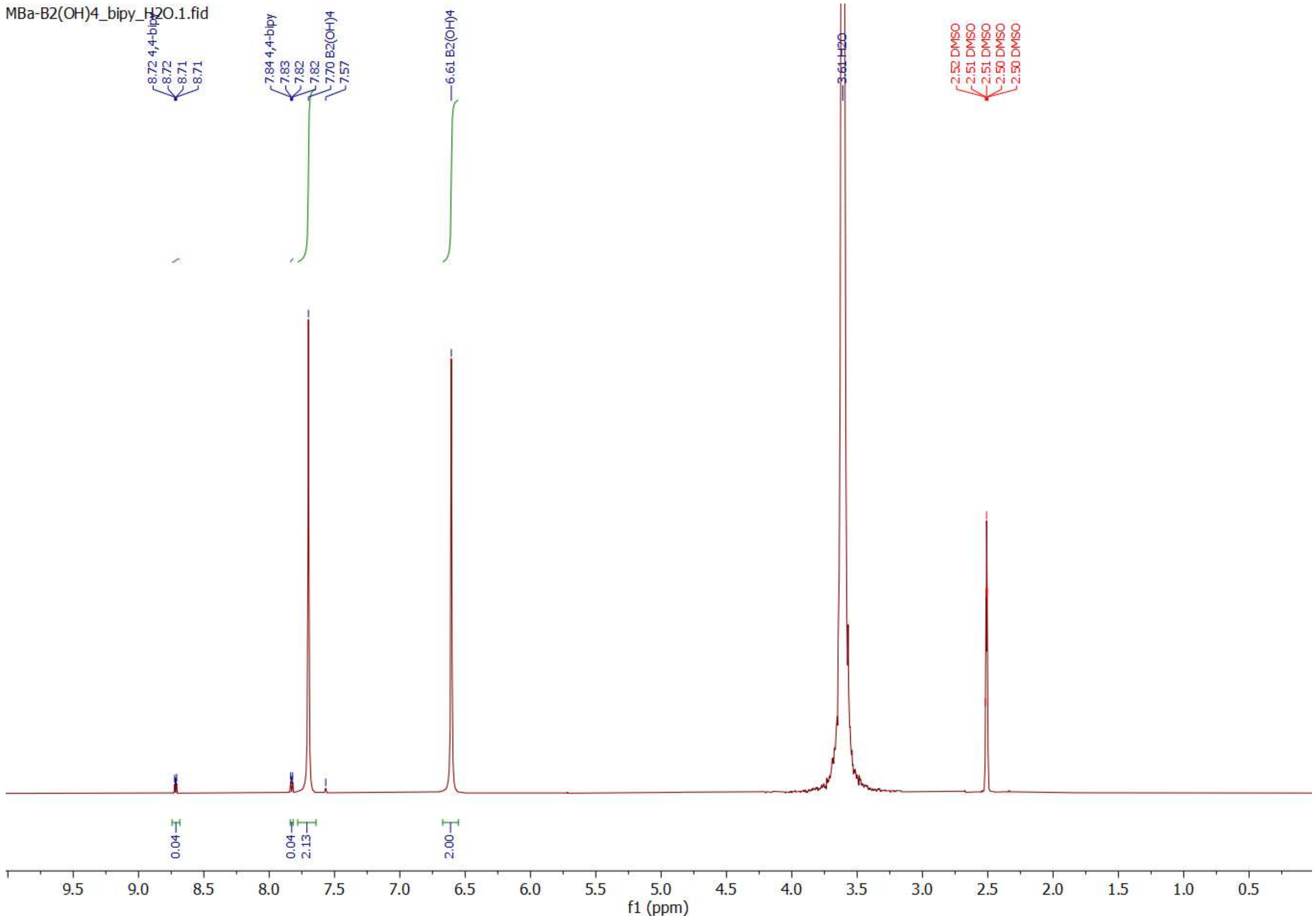

Figure S41 1H NMR Tetrahydroxydiboron, 4,4'-bipyridine and water in DMSO-*d6*.

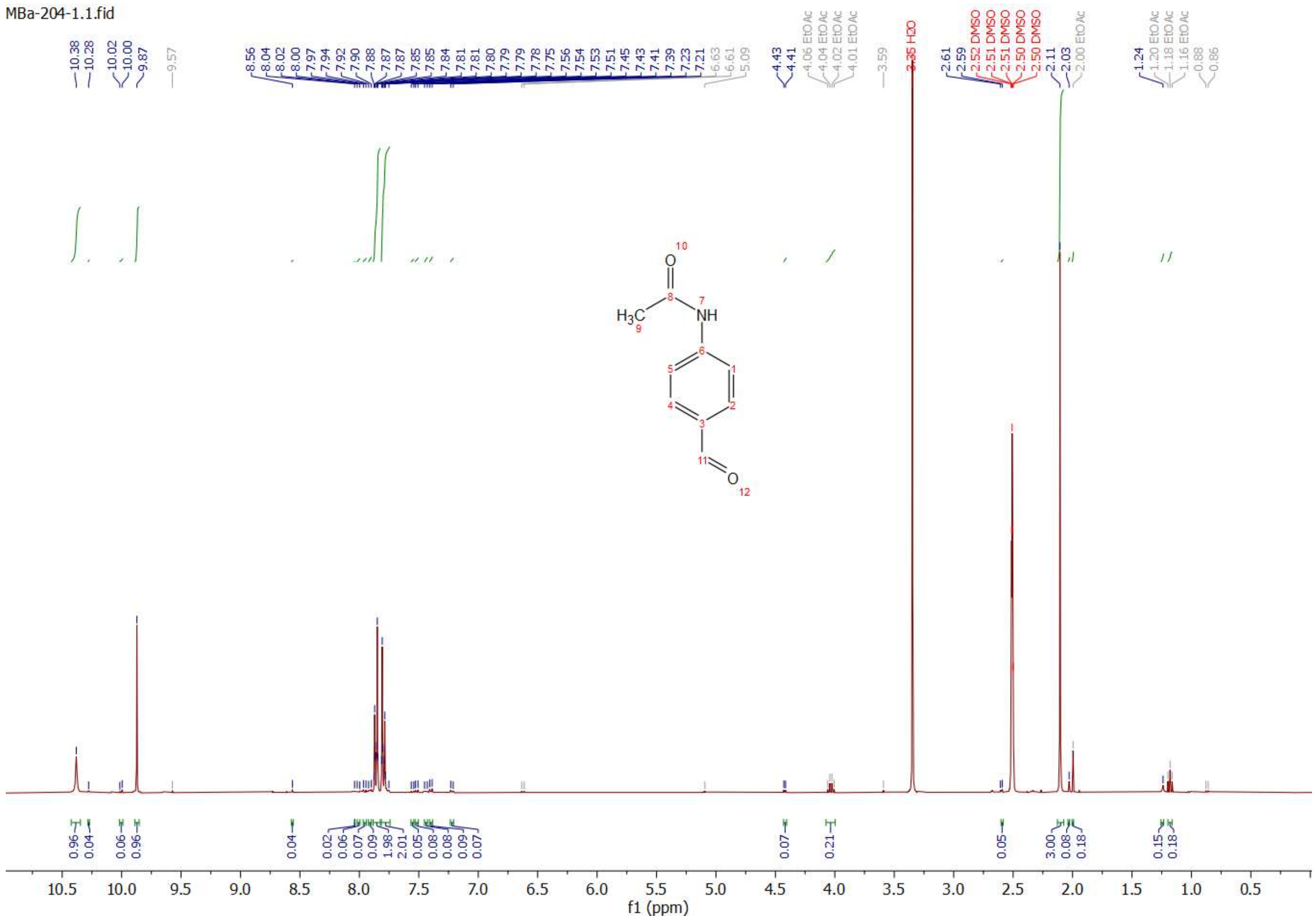

Figure S42 ^{1}H NMR **2kb** in DMSO-*d6*.

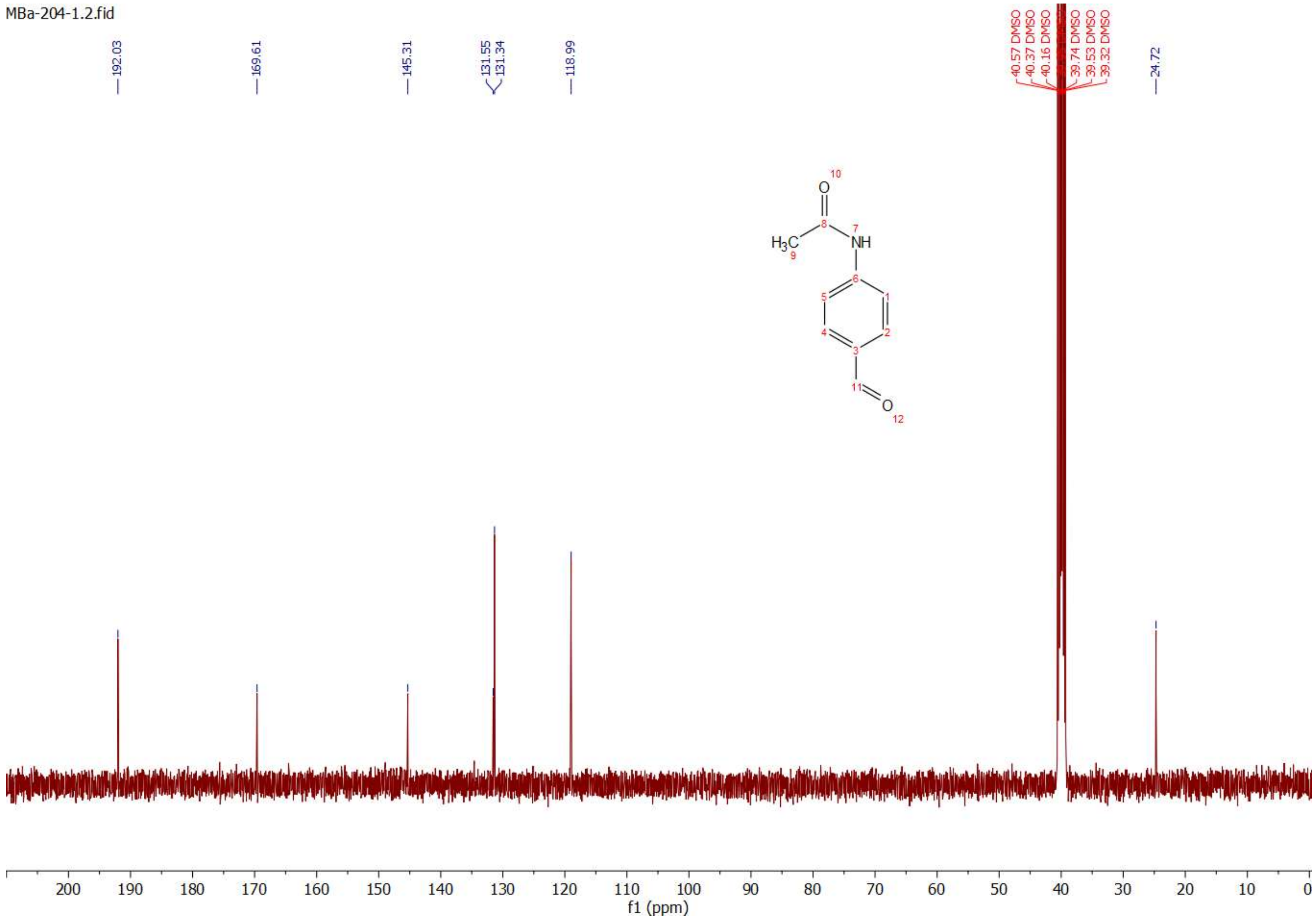

Figure S43 ^{13}C NMR **2kb** in DMSO-*d6*.

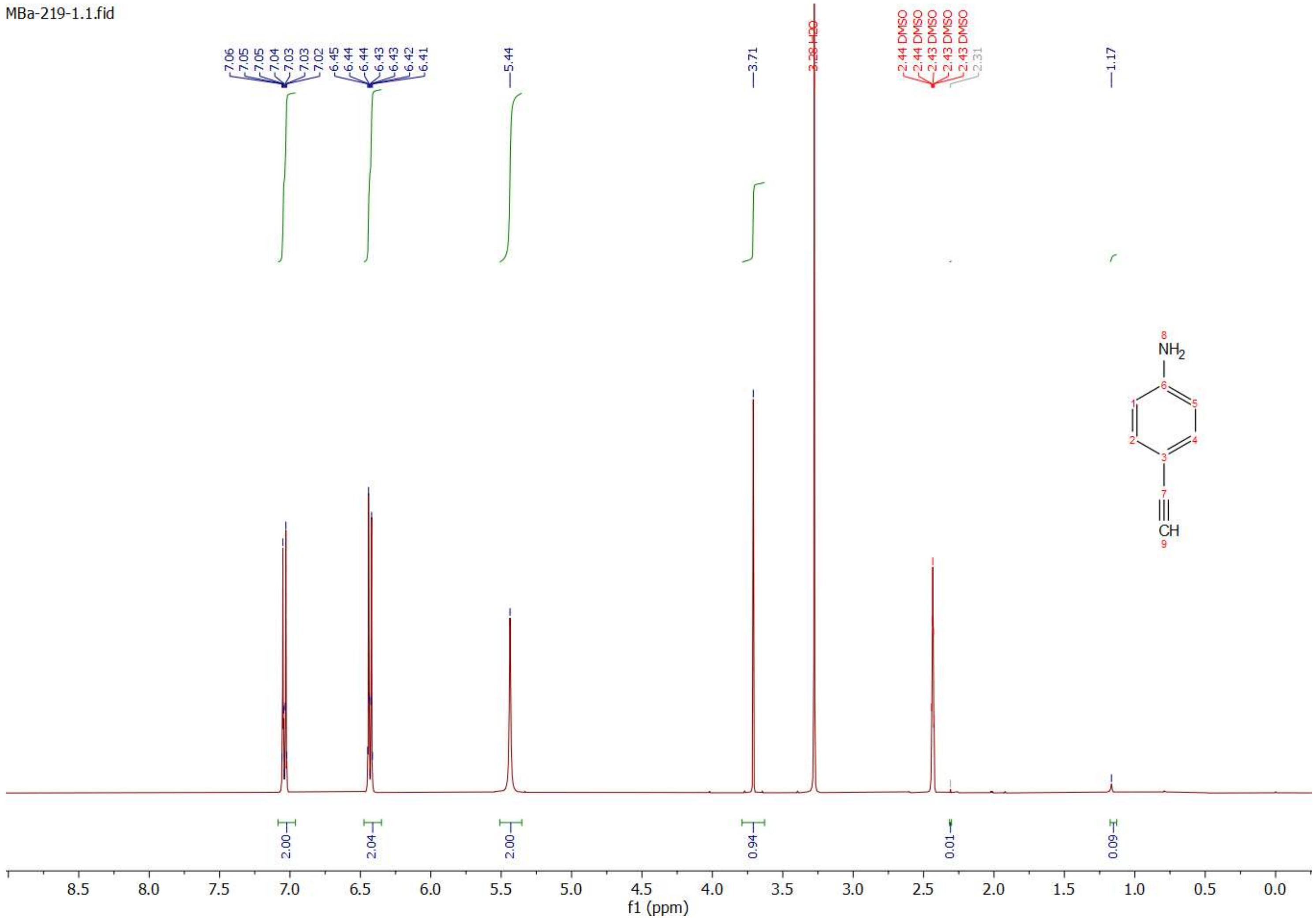

Figure S44 ^{1}H NMR **2l** in DMSO-*d6*.

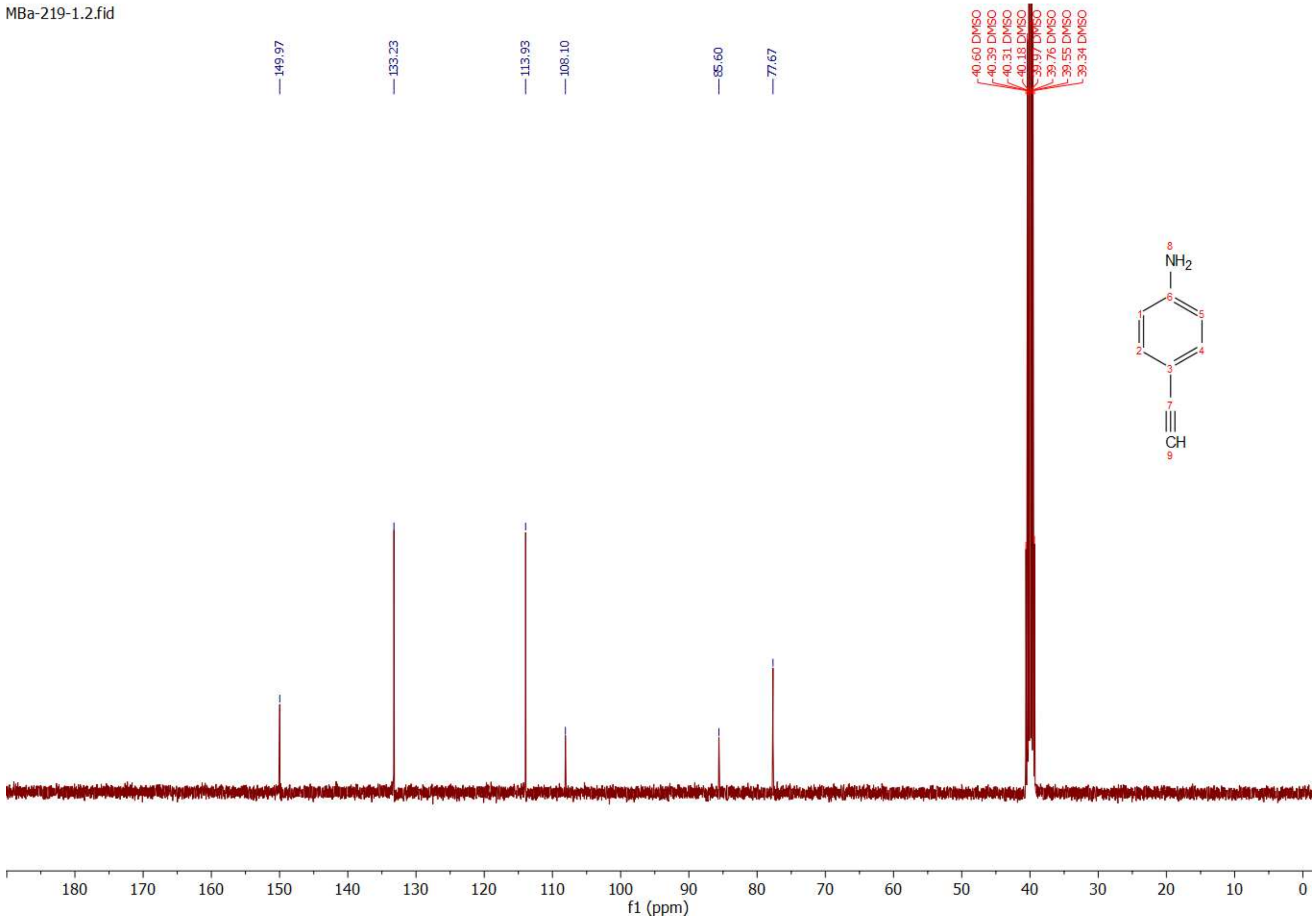

Figure S45 ^{13}C NMR **2l** in DMSO-*d6*.

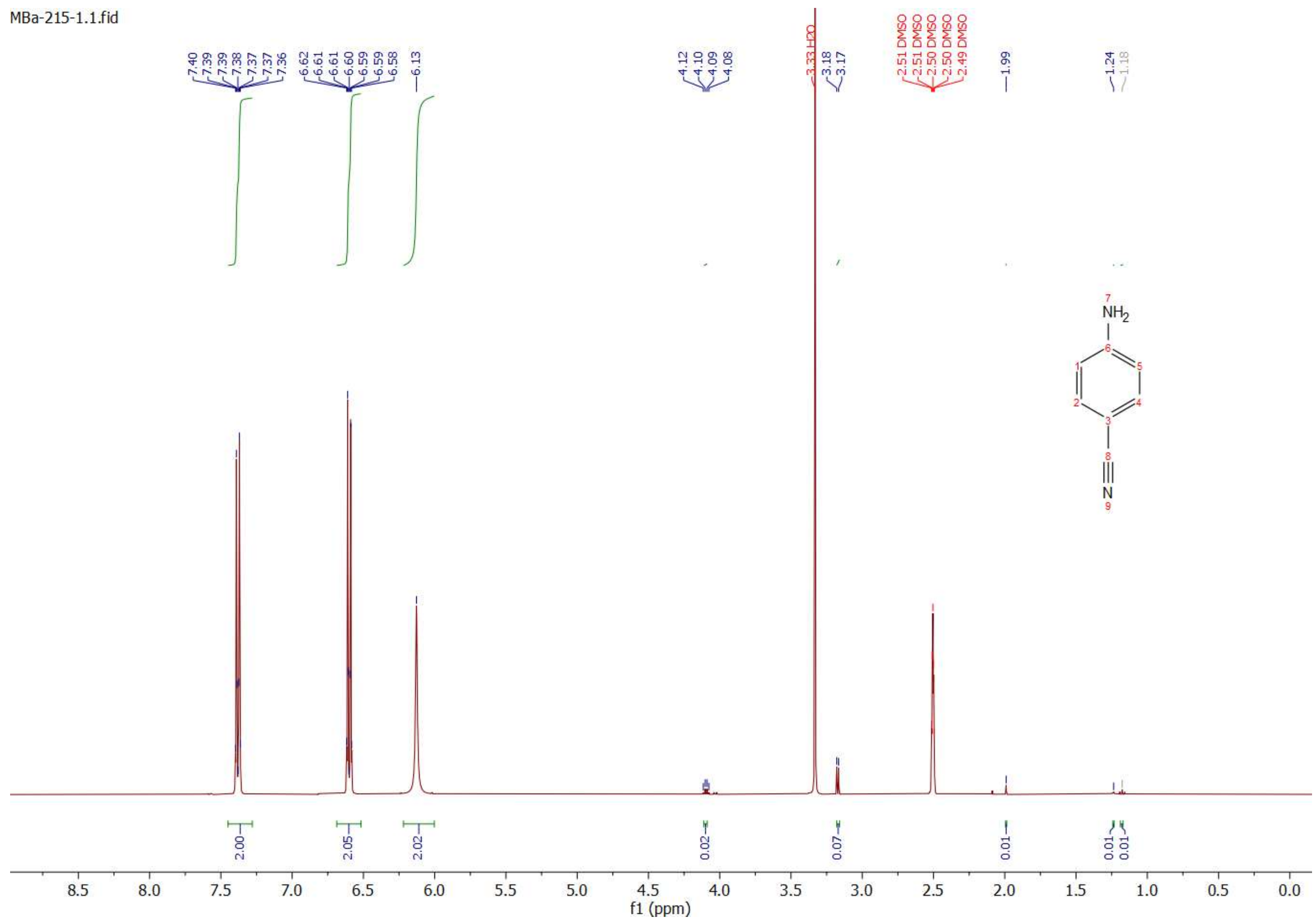

Figure S46 ^{1}H NMR **2m** in DMSO-*d6*.

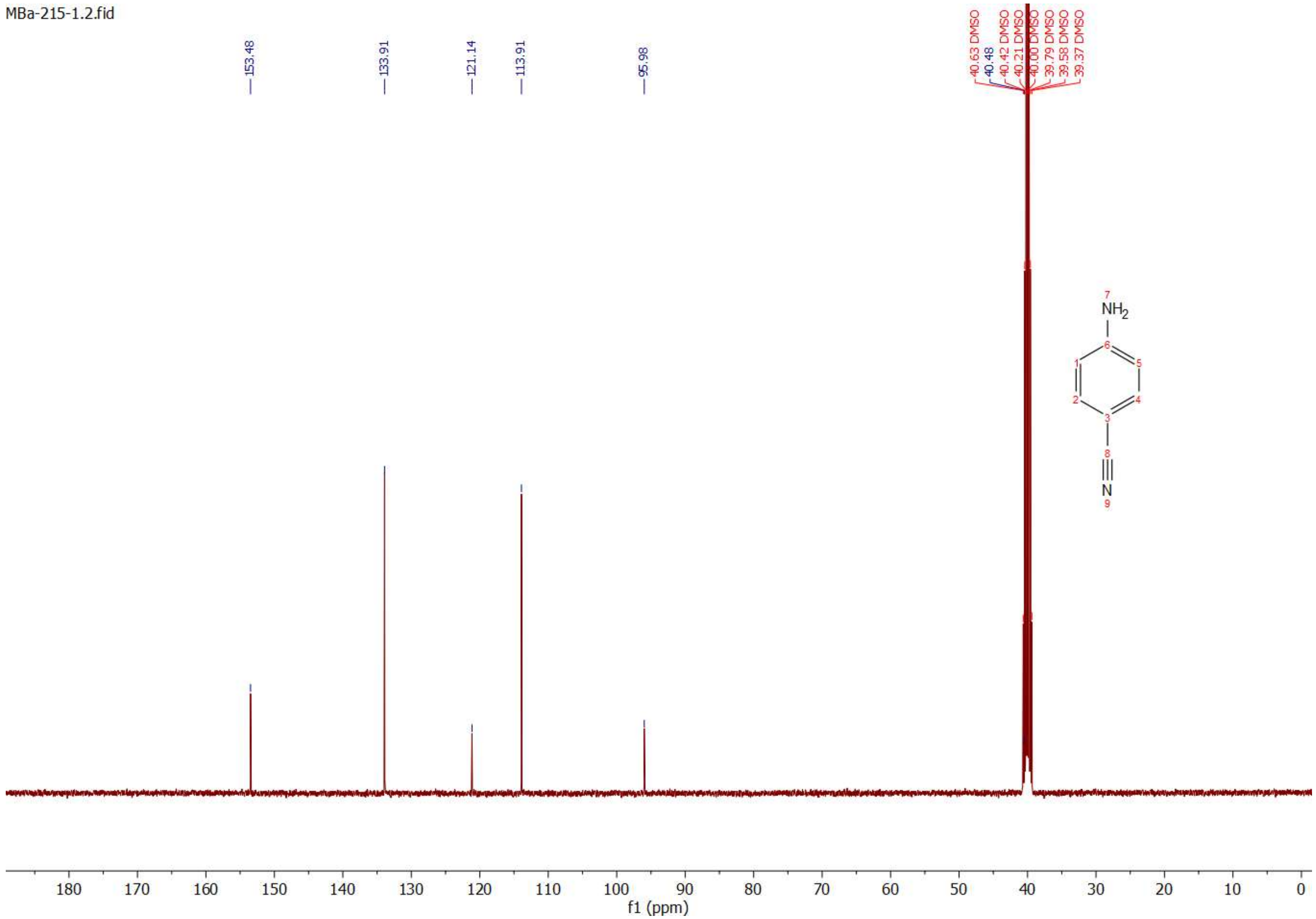

Figure S47 ^{13}C NMR **2m** in DMSO-*d6*.

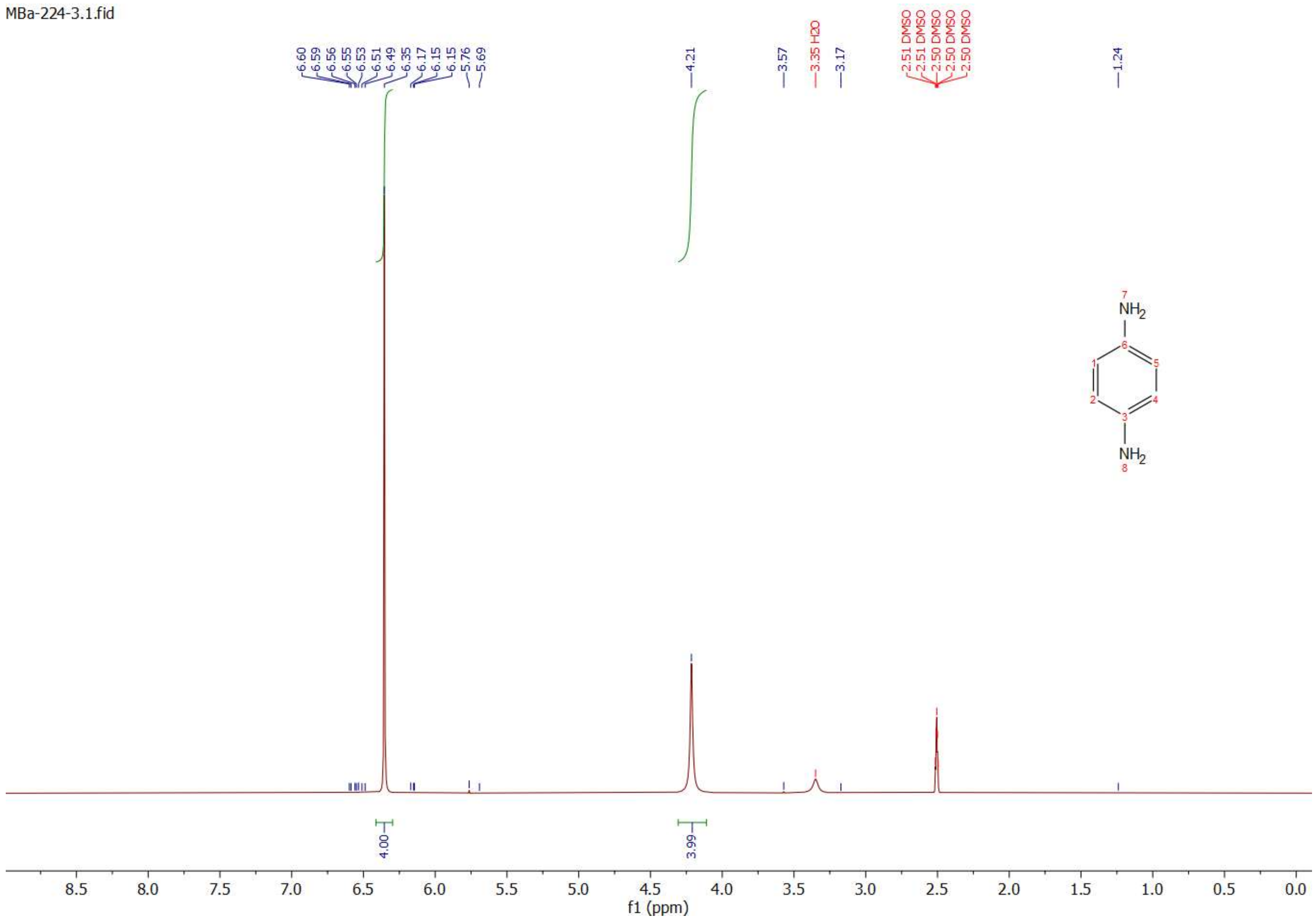

Figure S48 ^{1}H NMR **2n** in DMSO-*d6*.

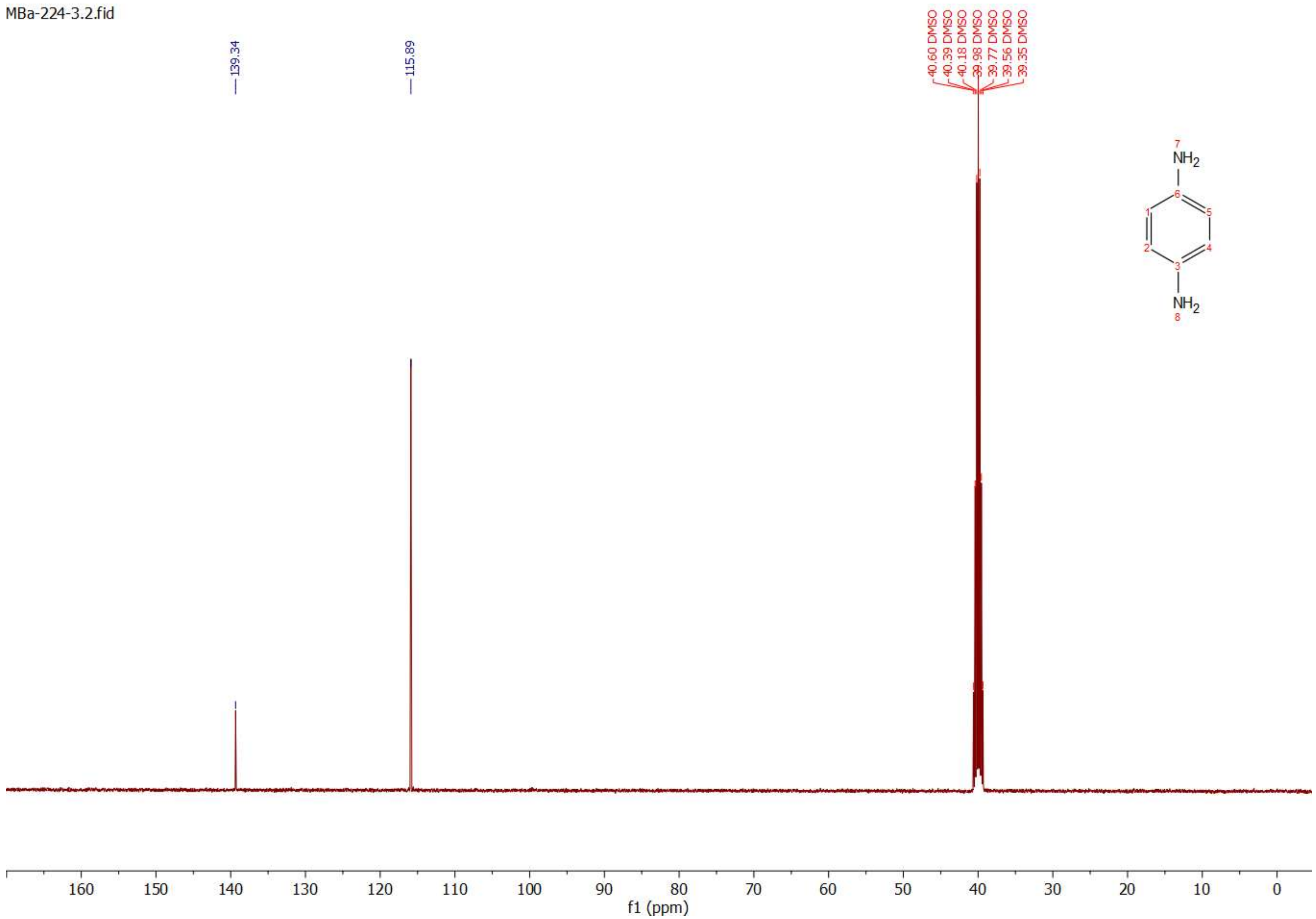

Figure S49 ^{13}C NMR **2n** in DMSO-*d6*.

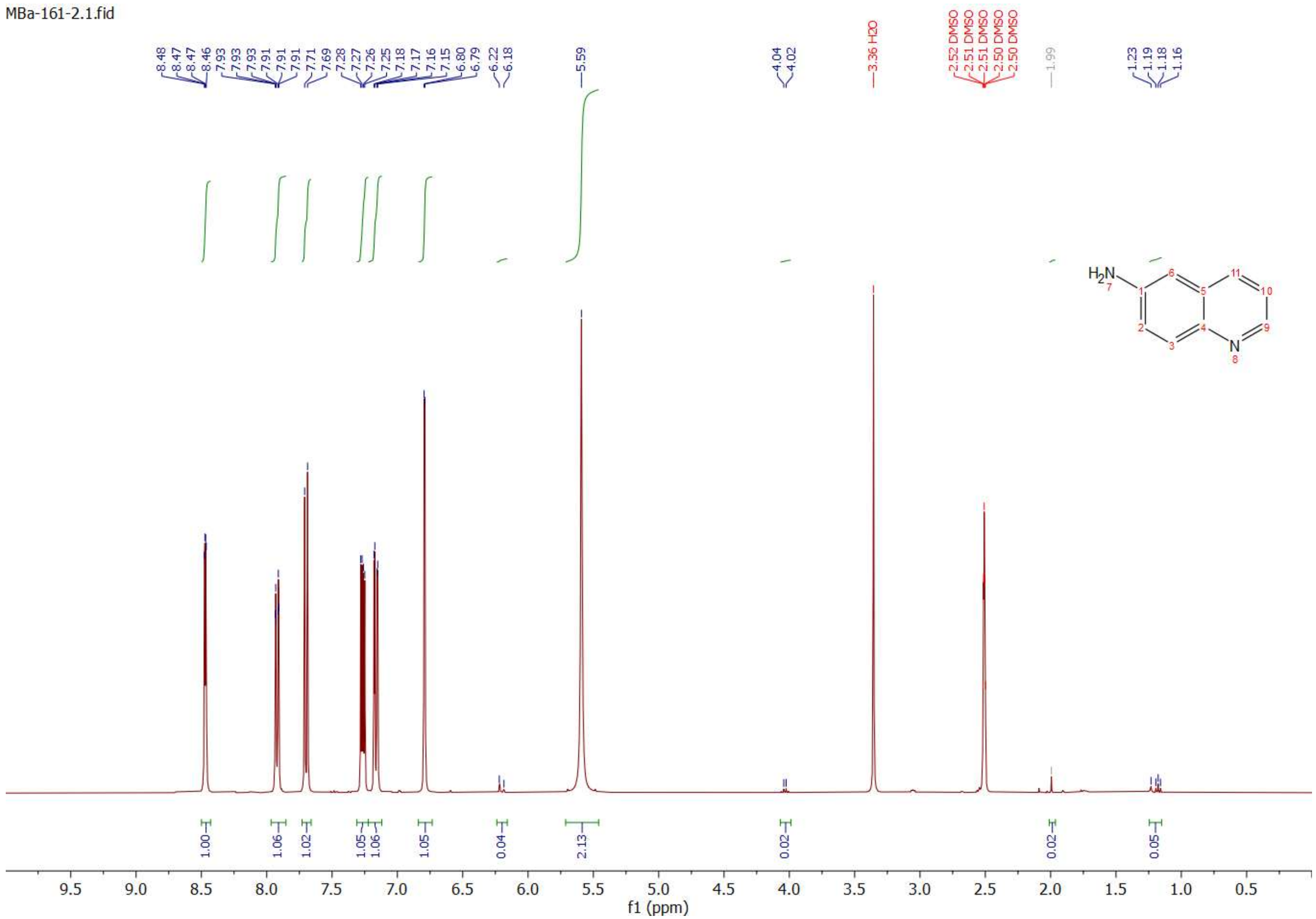

Figure S50 ^{1}H NMR **3aa** in DMSO-*d6*.

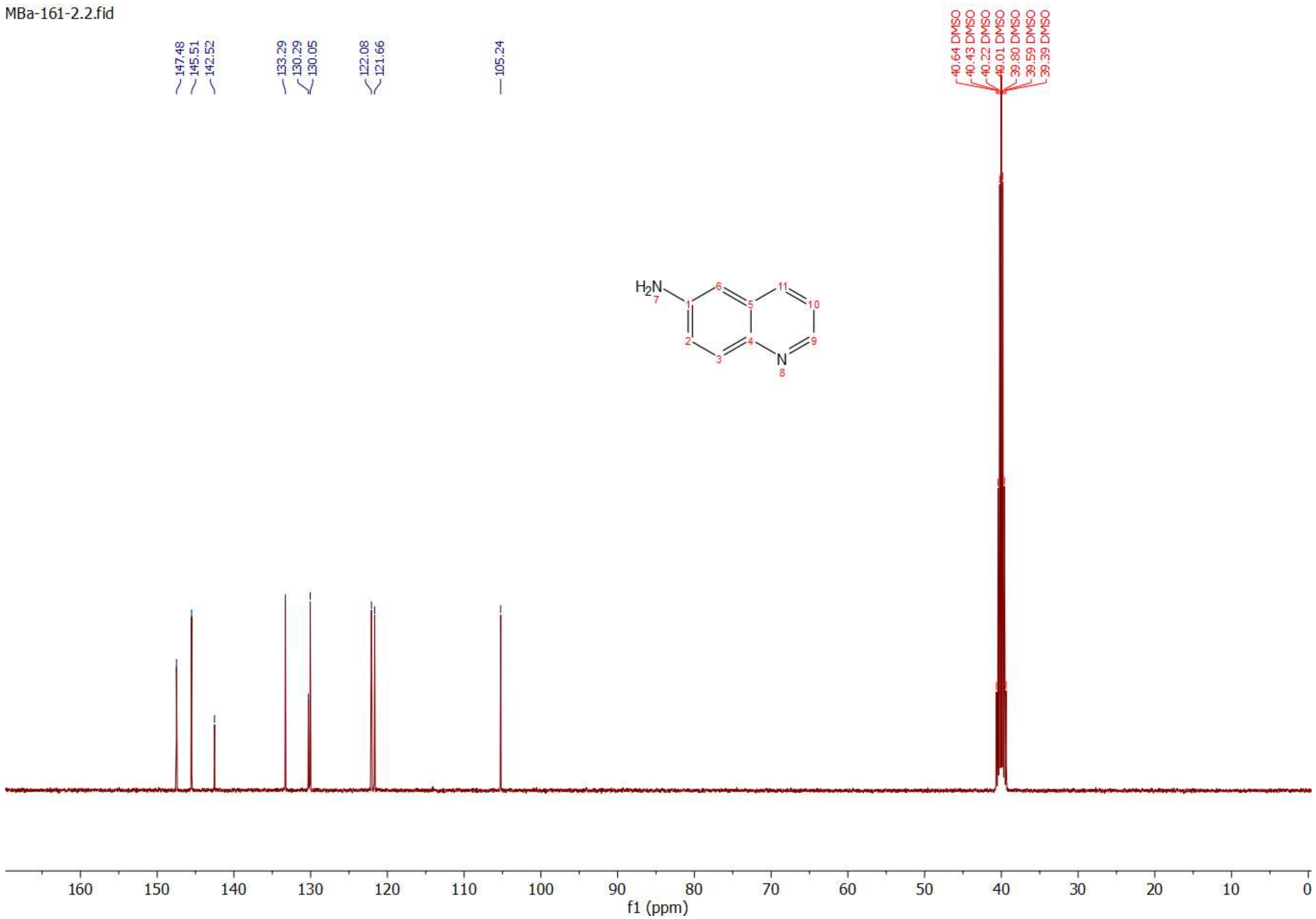

Figure S51 ^{13}C NMR **3aa** in DMSO-*d6*.

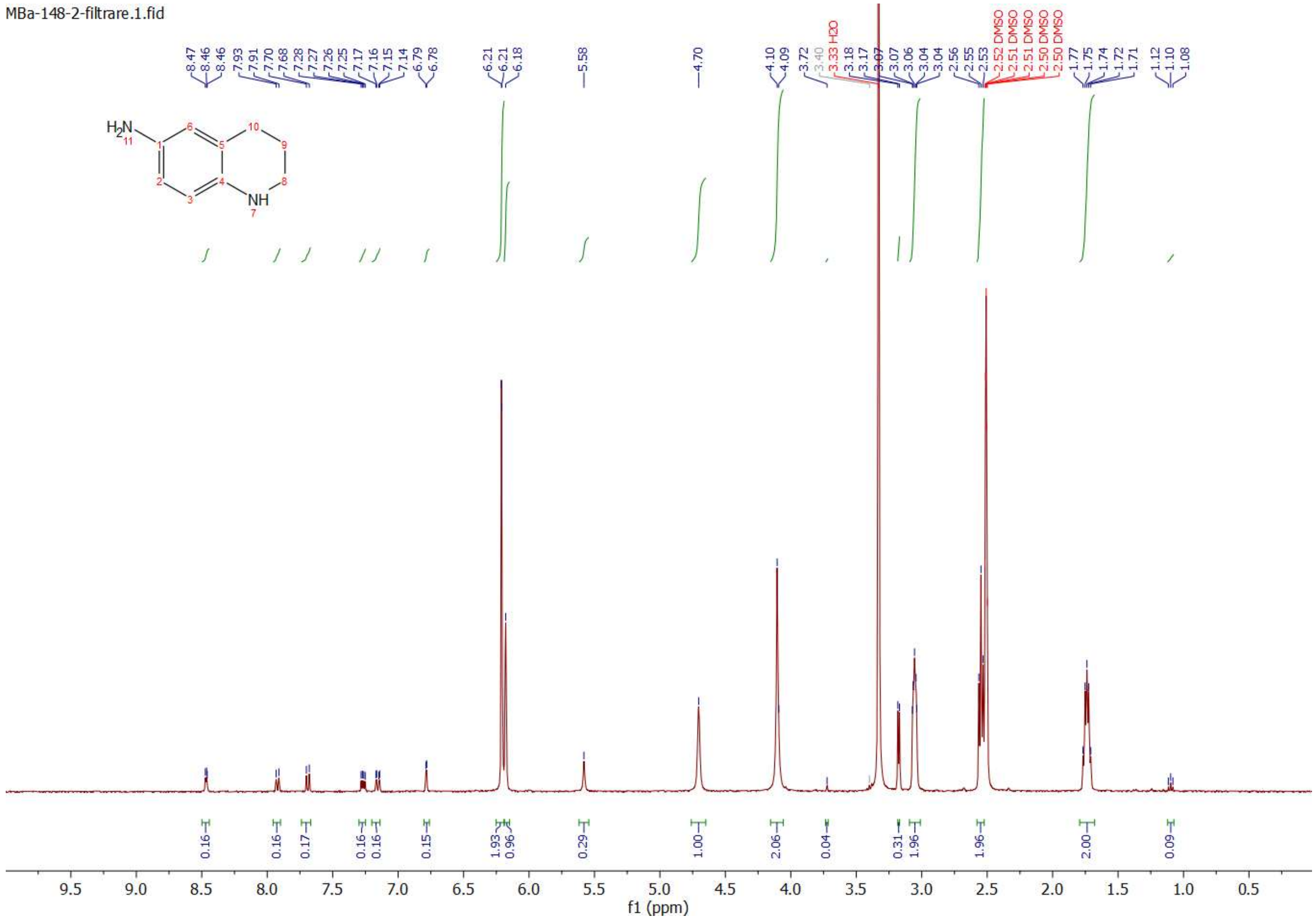

Figure S52 ^{1}H NMR **3ab** in DMSO-*d6*.

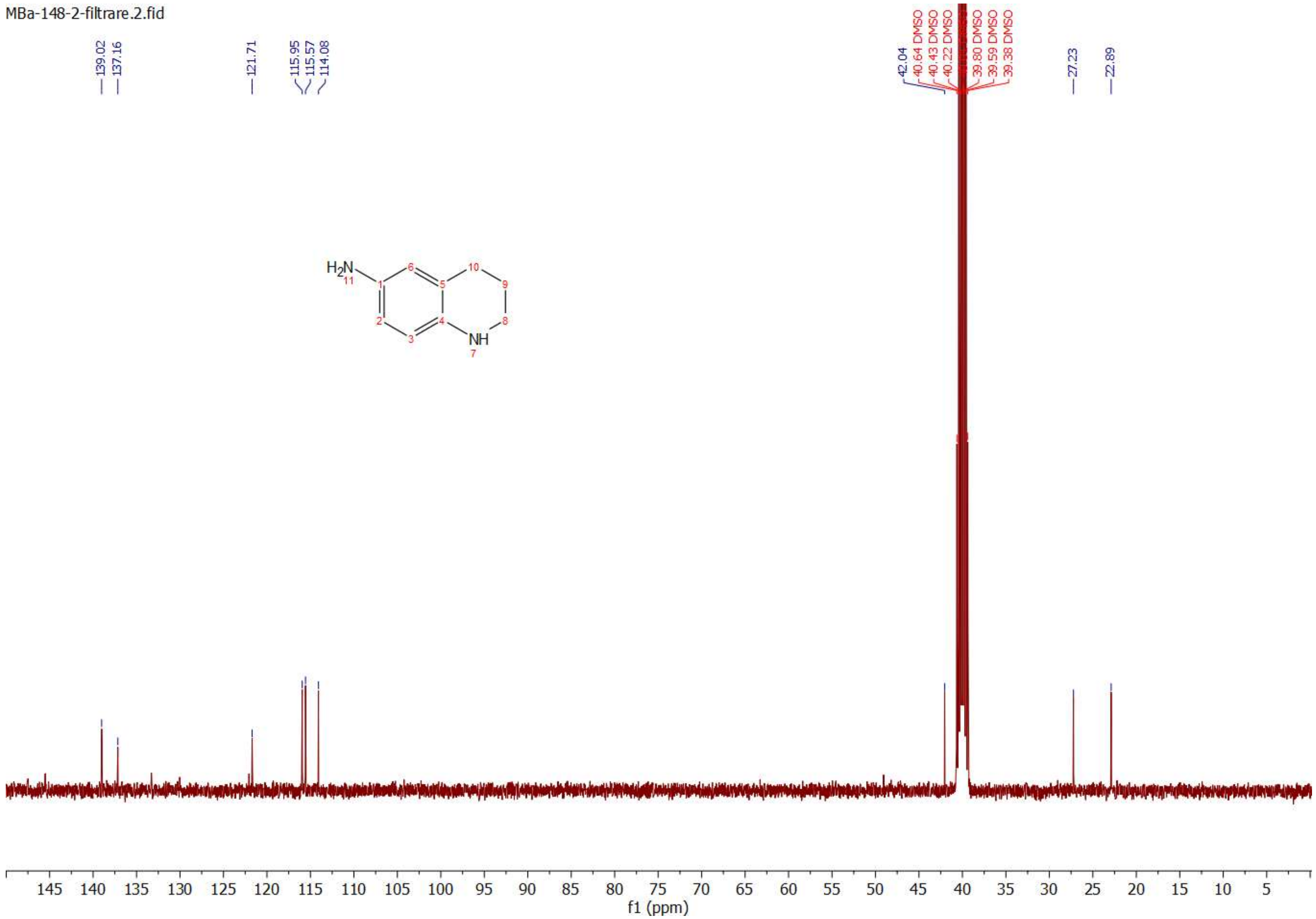

Figure S53 ^{13}C NMR **3ab** in DMSO-*d6*.

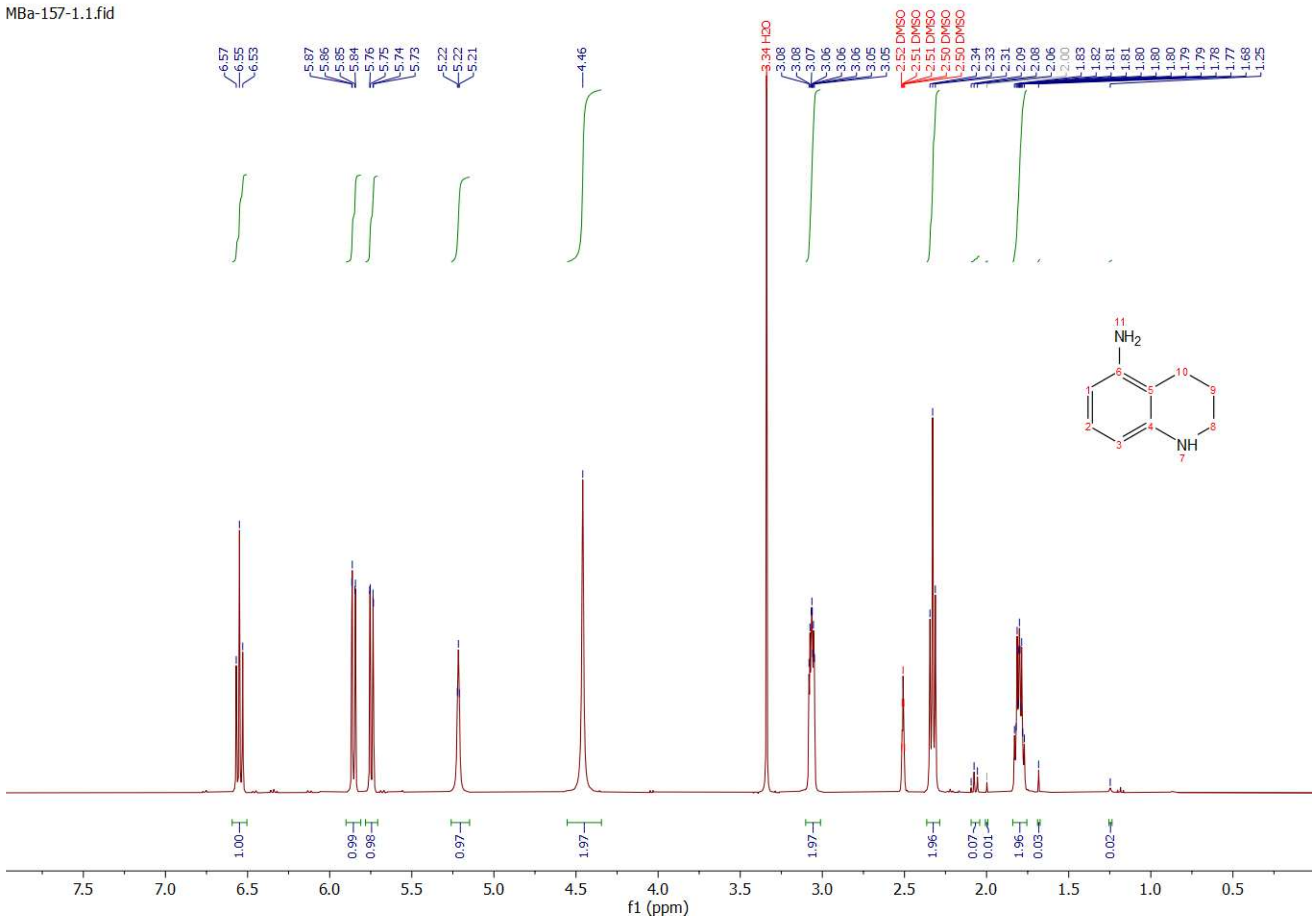

Figure S54 ^{1}H NMR **3b** in DMSO-*d6*.

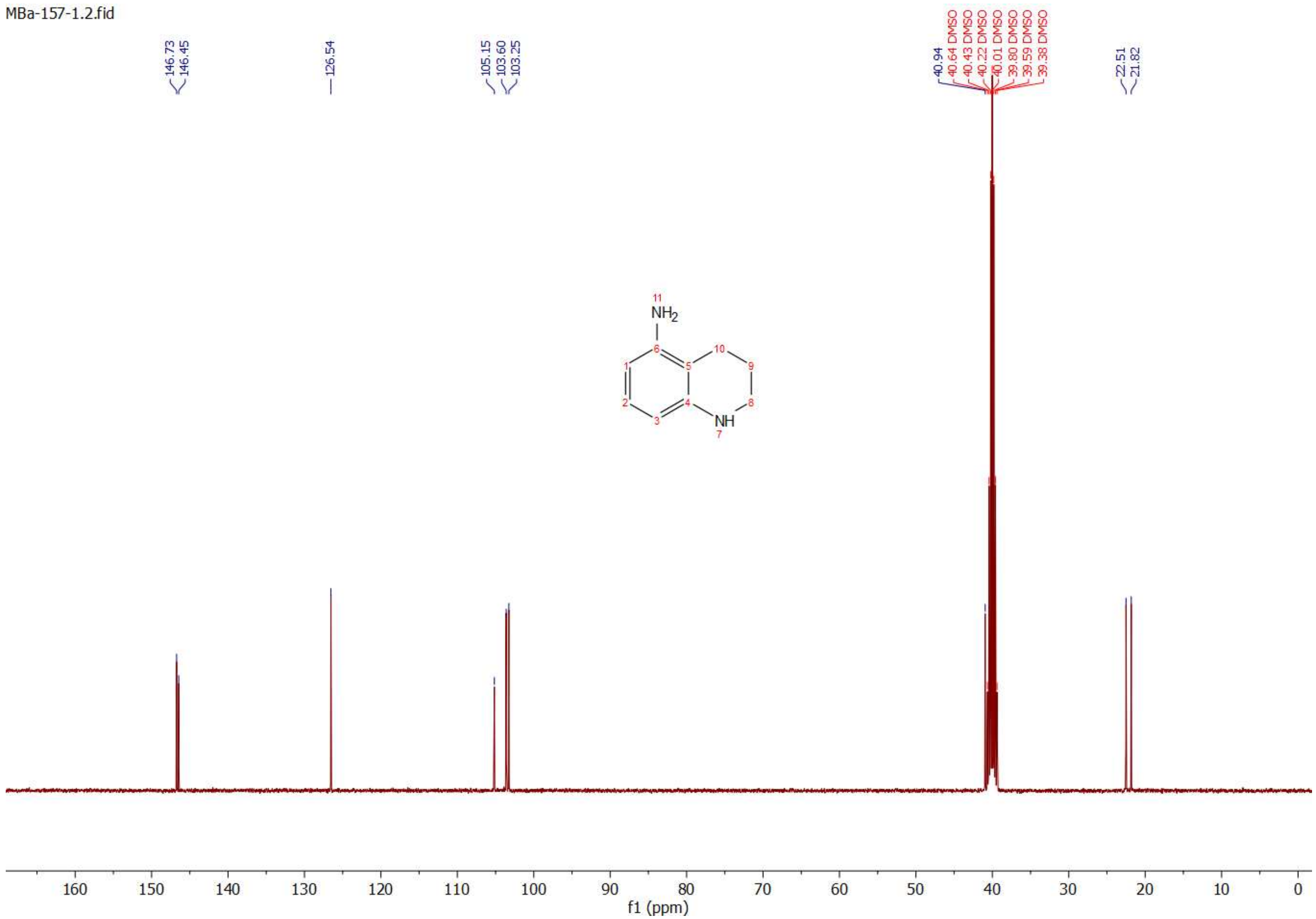

Figure S55 ^{13}C NMR **3b** in DMSO-*d6*.

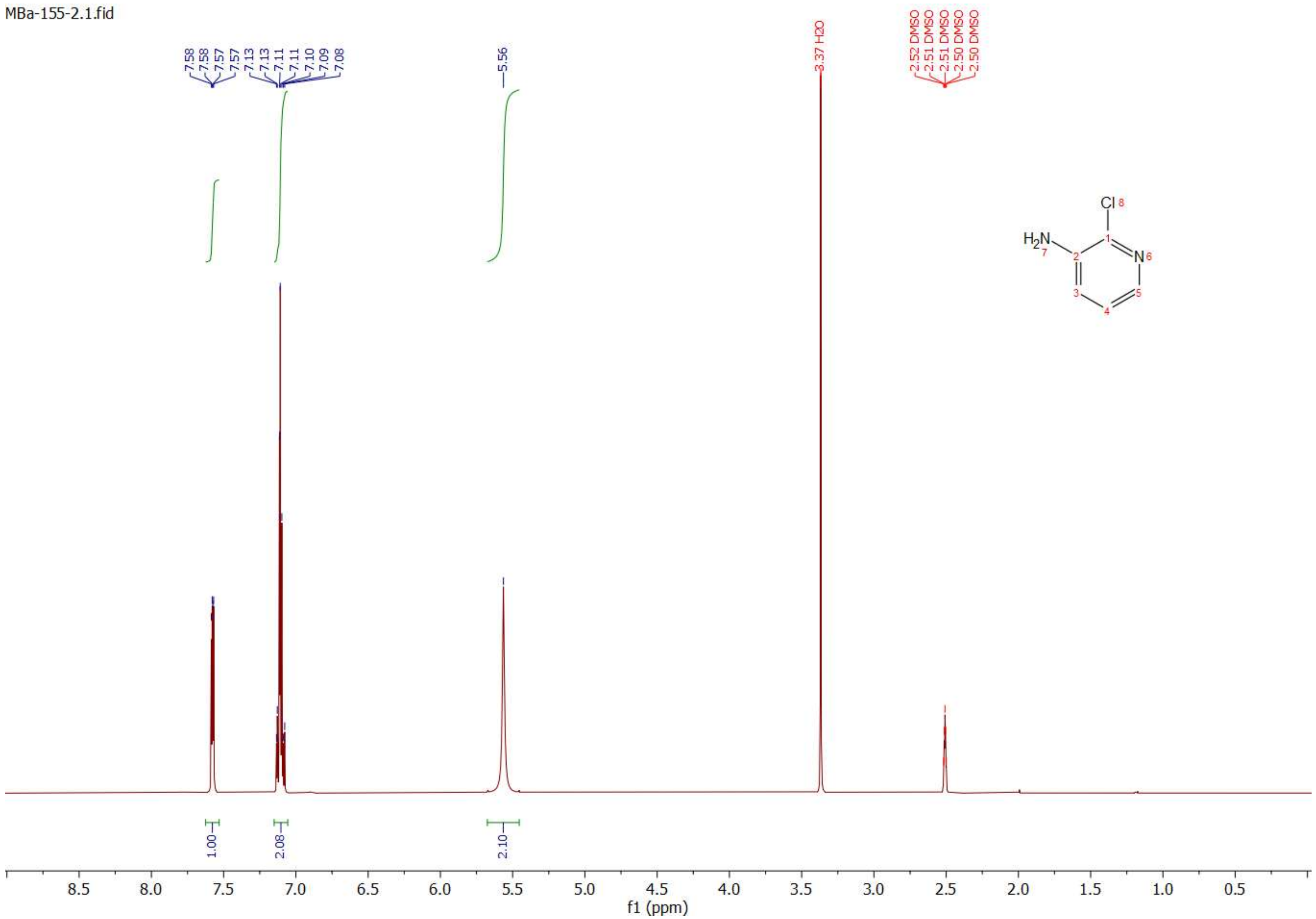

Figure S56 ^{1}H NMR **3d** in DMSO-*d6*.

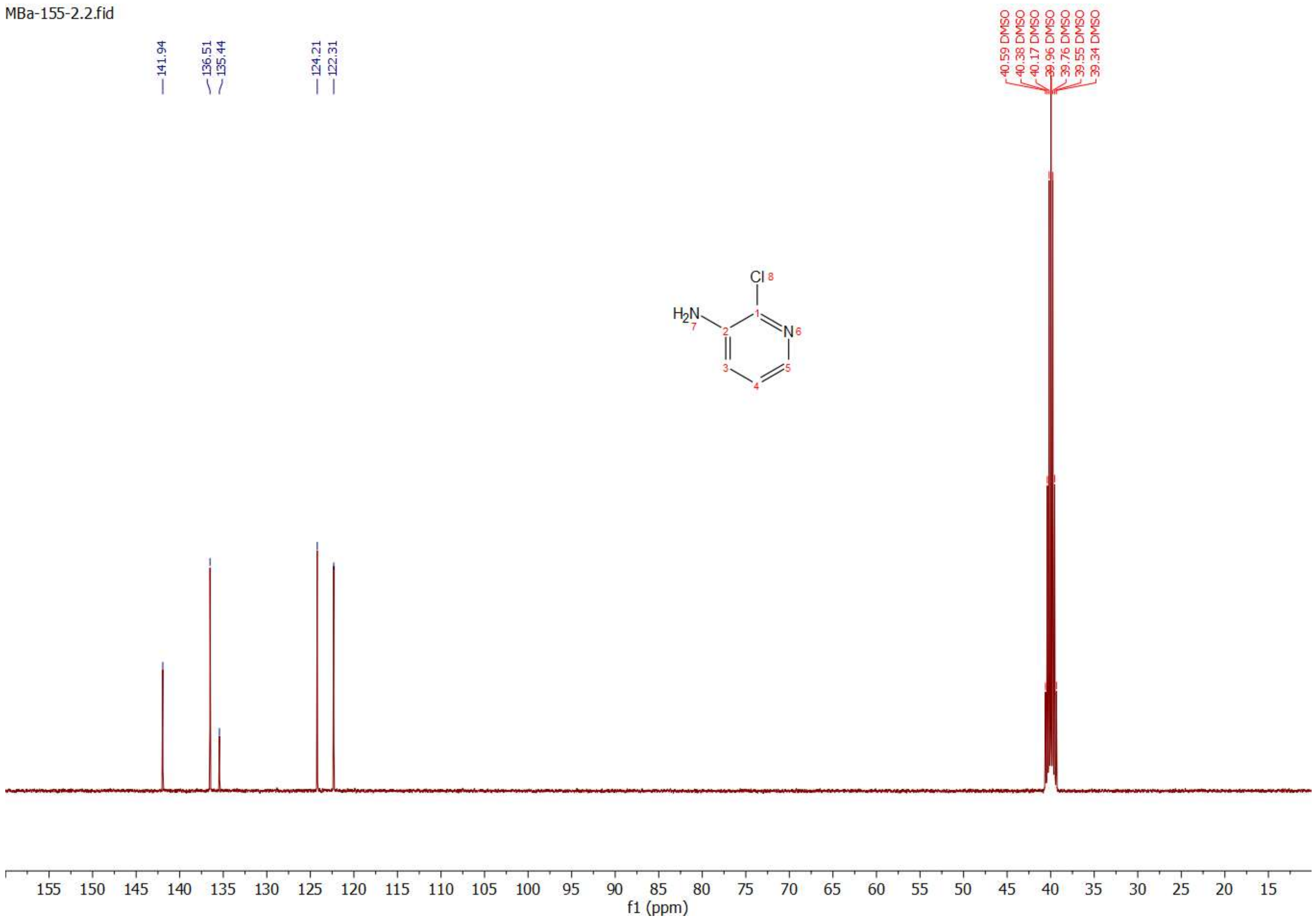

Figure S57 ^{13}C NMR **3d** in DMSO-*d6*.

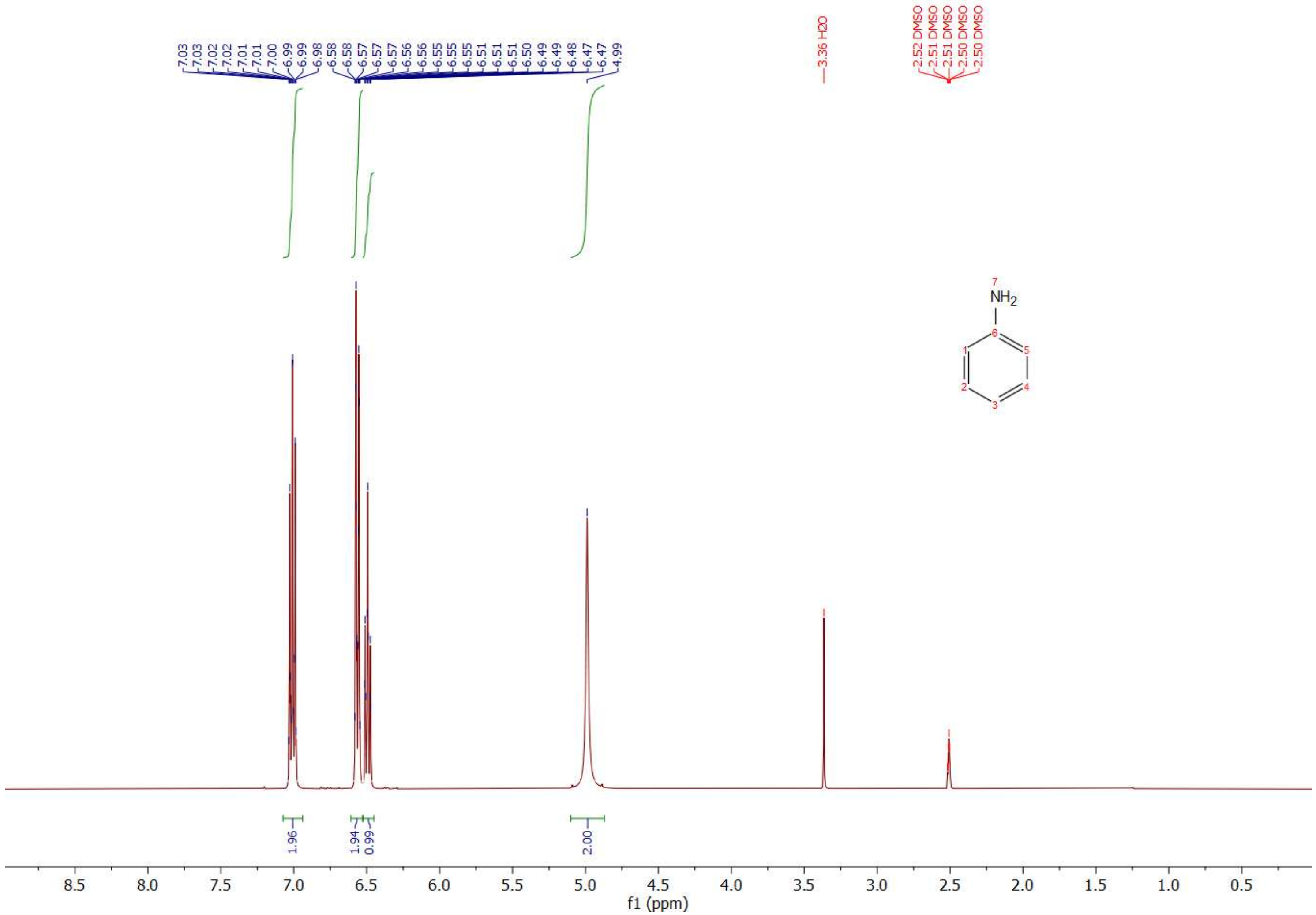

Figure S58 ^{1}H NMR **3e** in DMSO-*d6*.

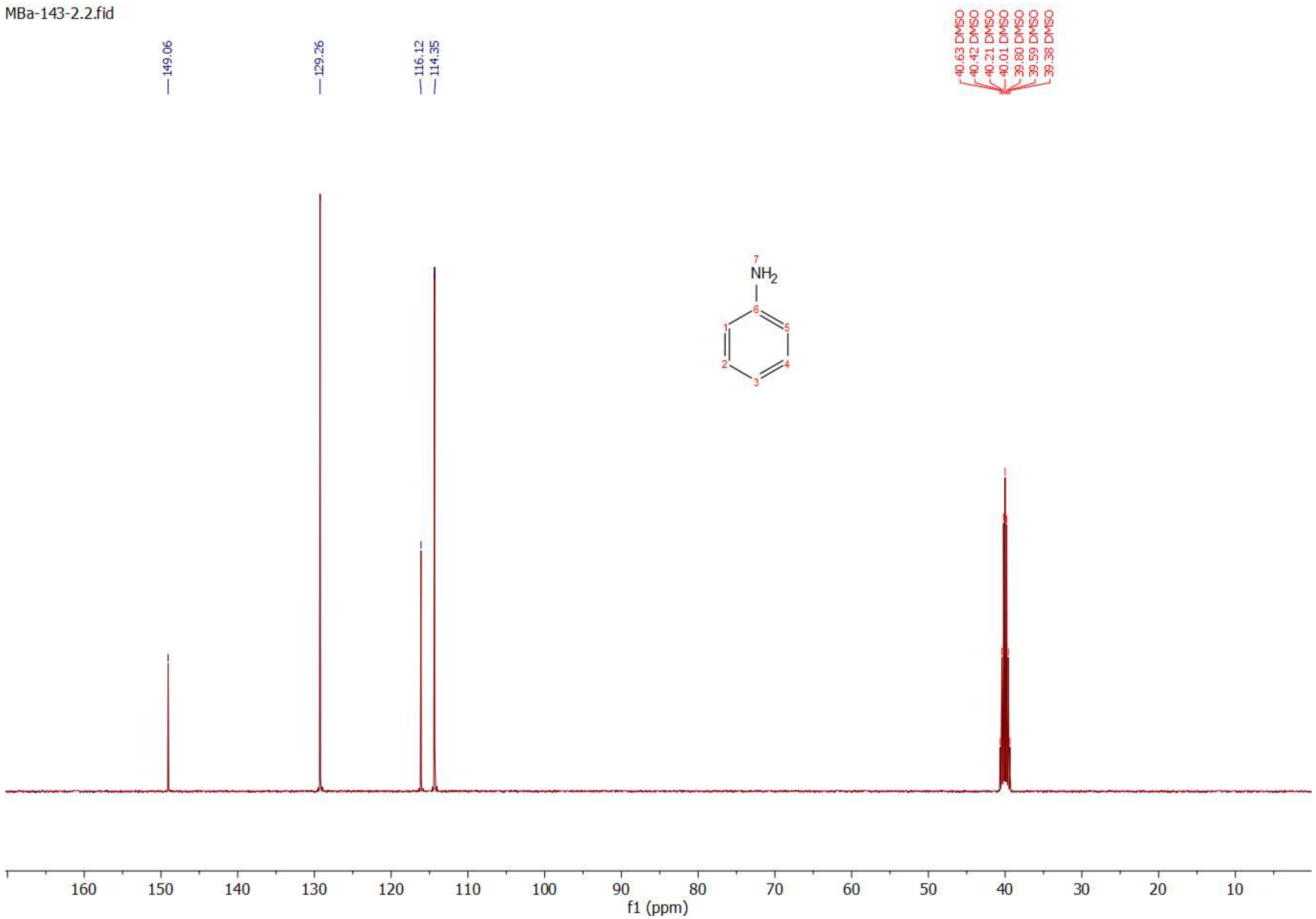

Figure S59 ^{13}C NMR **3e** in DMSO-*d6*.
